# Targeting a Multidrug-Resistant Pathogen: First Generation Antagonists of *Burkholderia cenocepacia*’s BC2L-C Lectin

**DOI:** 10.1021/acschembio.2c00532

**Published:** 2022-09-29

**Authors:** Rafael Bermeo, Kanhaya Lal, Davide Ruggeri, Daniele Lanaro, Sarah Mazzotta, Francesca Vasile, Anne Imberty, Laura Belvisi, Annabelle Varrot, Anna Bernardi

**Affiliations:** † CNRS, CERMAV, Univ. Grenoble Alpes, Grenoble 38000, France; ‡ Dipartimento di Chimica, Università degli Studi di Milano, via Golgi 19, Milano 20133, Italy

## Abstract

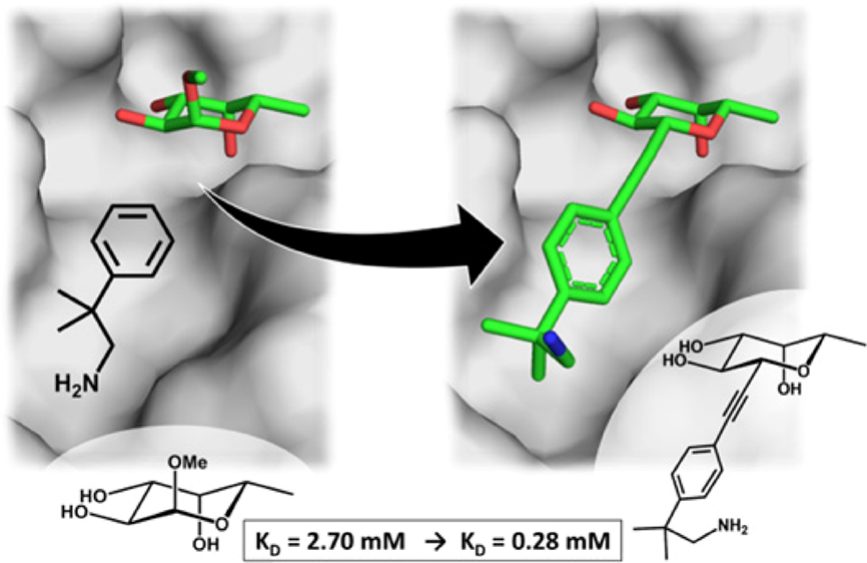

Multidrug-resistant pathogens such as *Burkholderia cenocepacia* have become a hazard in the context of healthcare-associated infections, especially for patients admitted with cystic fibrosis or immuno-compromising conditions. Like other opportunistic Gram-negative bacteria, this pathogen establishes virulence and biofilms through lectin-mediated adhesion. In particular, the *superlectin* BC2L-C is believed to cross-link human epithelial cells to *B. cenocepacia* during pulmonary infections. We aimed to obtain glycomimetic antagonists able to inhibit the interaction between the *N*-terminal domain of BC2L-C (BC2L-C-Nt) and its target fucosylated human oligosaccharides. In a previous study, we identified by fragment virtual screening and validated a small set of molecular fragments that bind BC2L-C-Nt in the vicinity of the fucose binding site. Here, we report the rational design and synthesis of bifunctional *C*- or *N*-fucosides, generated by connecting these fragments to a fucoside core using a panel of rationally selected linkers. A modular route starting from two key fucoside intermediates was implemented for the synthesis, followed by evaluation of the new compounds as BC2L-C-Nt ligands with a range of techniques (surface plasmon resonance, isothermal titration calorimetry, saturation transfer difference NMR, differential scanning calorimetry, and X-ray crystallography). This study resulted in a hit molecule with an order of magnitude gain over the starting methyl fucoside and in two crystal structures of antagonist/lectin complexes.

## Introduction

The rising threat of anti-microbial resistance poses a particular hazard to hospital-bound patients: opportunistic infections from multidrug-resistant (MDR) “superbugs” prey on the weakened systems of patients suffering from immuno-compromising conditions, cystic fibrosis, and other debilitating illnesses. These healthcare-associated infections become ever more worrisome in the current global context and have triggered action plans worldwide.^1,2^

Among the MDR pathogens, *Pseudomonas aeruginosa* and members of the *Burkholderia cepaciacomplex* (BCC) are notorious lung pathogens: they periodically surface in hospitals and hold the ability to form biofilms.^3,4^ Particularly hazardous for cystic fibrosis patients, pulmonary infections by BCC bacteria may lead to “cepacia syndrome”, a rapid decline of respiratory function leading to sepsis and high mortality rate. The main pathogen responsible for cepacia syndrome is *Burkholderia cenocepacia*, a globally spread bacterium.^4^ As for *P. aeruginosa* and many other pathogens, *B. cenocepacia’s* ability to colonize and invade host tissues strictly depends on bacterial adhesion,^5^ which is often mediated by carbohydrate/protein interactions. Indeed, human oligosaccharide antigens, particularly the histo-blood groups, are consistently targeted by microbial lectins that represent virulence factors in the context of infection.^6^ Disrupting bacterial lectin binding to host glycans prevents microbial adhesion, hinders the infective process at its inception, and is expected to lead to better clinical outcomes. This strategy, known as anti-adhesion therapy (AAT), is viable to complement and complete antibiotic therapy. AAT combats infection without killing the pathogen: compared to antibiotics, the lack of selective pressure is expected to reduce the appearance of mutations leading to AAT-resistant strains.^7,8^

Glycomimetics have proven their worth for AAT as reliable disruptors of carbohydrate/lectin interactions. Designed to emulate the carbohydrate structure and/or function, glycomimetics display improved physicochemical and pharmacokinetic properties (improved oral bioavailability, adjusted polarity, and metabolic stability). Such molecules have successfully been used to target both microbial and human carbohydrate-binding proteins known to be vectors of virulence.^9−12^ Nonetheless, the design of glycomimetics directed against specific lectin targets remains a challenging problem, owing to the intrinsically low affinity of carbohydrate–protein interactions and to the structural characteristics of lectin binding sites that are not well-suited for most of the classical tools of structure-based molecular design.^12,13^ Here, we describe the rational design, synthesis, and activity evaluation of fucose-based glycomimetic ligands targeted against a known adhesion factor of *B. cenocepacia*, the BC2L-C lectin.

BC2L-C was discovered through a genetic screening in *B. cenocepacia* for genes resembling the *lecB* gene of *P. aeruginosa*. In the extensively studied *P. aeruginosa*, LecB, encoded by the *lecB* gene, is a fucose-specific lectin involved in bacterial adhesion and biofilm formation. It represents a virulence factor and a major determinant for lung infection.^14−18^ In *B. cenocepacia*, the screening returned a lectin family composed of four proteins BC2L-A-D, all containing a LecB-like *C*-terminal dimeric domain.^19,20^ The operon bclACB coding for the three lectins is regulated by quorum sensing and plays a role in maintaining the structure of biofilm. Lectin-specific knockouts revealed that the lack of any of the three lectins led to defective biofilm.^21^ Additionally, genes coding for BC2L-B and -C were up-regulated across time on a single patient who suffered from chronic infection (cepacia syndrome).^22^ Taken together, these findings support additional investigation of BC2L- lectin targets for AAT. BC2L-C was also found to contain an *N*-terminal trimeric domain, which features specific millimolar affinity for L-fucose and high micromolar affinity for fucosylated histo-blood oligosaccharides.^23^ Thus, BC2L-C presents a double carbohydrate specificity, maintained through a hexameric assembly, which makes it a *superlectin*, hypothesized to crosslink cells by simultaneously binding bacterial mannosides in its *C*-terminal domain and human fucosides in the *N*-terminal domain.^20,23^

The inhibition of BC2L-C and of its crosslinking capacity provides a clear opportunity to employ AAT to reduce the effects of *B. cenocepacia* infections. Since its *C*-terminal domain is related to LecB, which has been extensively targeted and studied,^16−19,24^ we chose to focus on the fucose-specific *N*-terminal domain (BC2L-C-Nt). Our initial work focused on characterizing the recombinant version of BC2L-C-Nt (rBC2L-CN), its carbohydrate binding site, and its interaction with histo-blood group ligands H-type 1 and H-type 3 (Globo H).^25^ Thanks to the structural data obtained, computational work allowed mapping the protein surface for potential ligandable sites. A fragment screening campaign validated by X-ray data provided a small library of molecular entities predicted to bind near the fucose-occupied binding site.^26^ Based on these results, we now have rationally designed and synthesized a panel of first-generation BC2L-C-Nt glycomimetic ligands. We probed these molecules against their target through different biophysical techniques and identified a hit compound. The leading fucoside glycomimetic showed an affinity increase of nearly one order of magnitude over the starting monosaccharide α-Me-L-Fucoside (αMeFuc). Furthermore, we obtained the crystal structures of BC2L-C-Nt in complex with the lead structure and with a second ligand.

## Methods

### Synthesis

When anhydrous conditions were required, the reactions were performed under a nitrogen or argon atmosphere. Anhydrous solvents were purchased from Sigma-Aldrich with a content of water ≤0.005%. Triethylamine (Et_3_N), methanol, and dichloromethane were dried over calcium hydride. THF was dried over sodium/benzophenone and freshly distilled. *N*,*N*-Dimethylformamide (DMF) was dried over 4 Å molecular sieves. Reactions were monitored by analytical thin-layer chromatography (TLC) performed on Silica Gel 60 F_254_ plates (Merck) and TLC Silica gel 60 RP-18 F_254_s (Merck), which were analyzed with UV detection (254 and 365 nm) and/or staining with ammonium molybdate acid solution, potassium permanganate alkaline solution, ninhydrin stain, and Dragendorff stain. Flash column chromatography was performed using silica gel 60 (40–63 μm, Merck). Automated flash chromatography was performed with a Biotage Isolera Prime system, and SNAP ULTRA cartridges were employed. For HPLC purifications, a Waters 600 controller coupled to a Waters 2487 Dual Absorbance Detector (214 and 250 nm) was used at a flow rate of 22.0 mL/min (Varioprep column: 250/21 mm nucleosil 100–7 C_18_). The gradient used was linear from H_2_O (0.1% TFA) to CH_3_CN 9/1 H_2_O (0.1% TFA). NMR experiments were carried out on a Bruker AVANCE 400 MHz instrument at 298 K. The ^13^C-NMR spectra are Attached Proton Test J-modulated spin-echo (APT). Mass spectra were recorded on a Thermo Fischer LCQ apparatus (ESI ionization). High resolution mass spectra were recorded on spectrometers Apex II ICR FTMS (ESI-HRMS) or Thermo Fischer LTQ Orbitrap XL (ESI-HRMS) or a VG AutoSpec M246 (Fisons) spectrometer equipped with EBE geometry and EI source (EI-HRMS). The β-fucosylazide **2** is a known compound and was prepared following the method reported by Palomo et al.^27^ Methyl α-L-fucopyranoside **13** is commercially available and was purchased from Carbosynth.

### Computational Method

All docking calculations were performed using the Schrödinger Suite through a Maestro (version 2018-1) graphical interface.^28^ Atomic coordinates of the crystal structure of BC2L-C-Nt with MeSe-α-L-Fuc (PDB code 2WQ4) were taken from the Protein Data Bank.^29^ The asymmetric unit involves three peptide chains with three identical carbohydrate ligands (MeSe-α-L-Fuc) around a threefold pseudo axis of symmetry. The sugar at the three binding sites displays an identical binding pose; therefore, only one binding site (located between chains A and C) was used for docking calculations. The structural water molecules HOH2195 (W1) and HOH2194 (W2) were retained in the binding site region. The hydrogen atoms were added, and p*K*_a_ was calculated for protein residues using the PROPKA method^30−32^ at pH 7.4. The HIE protonation state was also assigned to histidine (His116) residue. Thereafter, the protein–ligand complex was minimized by applying convergence of heavy atoms to RMSD of 0.3 Å using the OPLS3 force field.^33^ The glycomimetic ligands were prepared for docking using the LigPrep tool.^34^ The protonation states were generated at pH 7 ± 2. The docking grid was prepared without fucose, while retaining the two water molecules (W1 and W2) mentioned above. The fucoside centroid was located in the active site between chain A and chain C in order to define a cubic grid box with dimensions 32 × 32 × 32 Å. The selenium atom in the crystal structure (MeSe-α-L-Fuc) was replaced by oxygen, and the metylfucoside was redocked at the sugar binding site. The protocol reproduced the co-crystallized pose (RMSD 0.1 Å), hence validating the docking protocol using Glide (version 7.8).^35^ The glycomimetic ligands designed using the best fragments from virtual screening were studied using XP and SP approaches in Glide.^35^

### Isothermal Titration Calorimetry

All experiments were performed at 25 °C with an ITC200 isothermal titration calorimeter (Microcal-Malvern Panalytical, Orsay, France). The protein rBC2L-CN2 and its ligands were dissolved in a buffer composed of 20 mM Tris HCl pH 7.0 and 100 mM NaCl. The 200 μL sample cell containing rBC2L-CN (concentrations ranging from 200 to 400 μM) was subjected to injections of ligand solution: 20 to 39 injections of 1 μL or 70 injections of 0.5 μL (5 to 50 mM, chosen depending on the ligand) at intervals of 100, 120, or 200 s, while stirring at 850 rpm. Control experiments were performed by repeating the same protocol, but injecting the ligand into buffer solution. The supplied software Origin 7 or MicroCal PEAQ-ITC was used to fit the experimental data to a theoretical titration curve allowing the determination of affinity.

### Surface Plasmon Resonance

Experiments were performed on a BIACORE X100 instrument (GE Healthcare) at 25 °C in running buffer 10 mM HEPES pH 7.4, 150 mM NaCl, and 0.05% Tween 20, adjusted to include 8% DMSO when required. rBC2L-CN2 was immobilized onto CM5 chips (BIACORE) following the amine coupling procedure detailed in the Supporting Information. The analytes were dissolved in the running buffer at increasing concentrations (range: 3.28–3500 μM) and subjected to multi-cycle affinity studies (300 s association, 300 s dissociation, flow rate 5 μL/min). Injections of compounds at increasing concentrations onto the immobilized rBC2L-CN2 were followed by regeneration of the surface: 10 mM fucose in running buffer and then running buffer at 5 μL/min (100 and 150 s, respectively) after each analyte association/dissociation. For the higher concentrations, regeneration was secured by performing one or more runs replacing analyte by running buffer. Duplicates were performed for all ligands. Binding affinity (*K*_D_) was measured after subtracting the channel 1 reference (no immobilized protein) and subtracting a blank injection (running buffer—zero analyte concentration). Data evaluation and curve fitting were performed using the provided BIACORE X100 evaluation software (version 2.0). The protein-coated chip was stored at 4 °C in running buffer and was functional up to 8 weeks after fabrication, as proven by experimentation.

### Differential Scanning Calorimetry

Experiments were performed on a Microcal PEAQ-DSC instrument (Malvern Panalytical, Orsay, France). A buffer composed of 20 mM Tris HCl pH 7.0 and 100 mM NaCl was used to dilute the protein rBC2L-CN2 and its ligands to concentrations 14.3 and 143 μM, respectively. Samples of 250 μL were loaded, while the reference cell was filled with the matching buffer (aforementioned buffer, ligands when relevant). Each sample was subjected to a gradient of temperature from 20 to 130 °C at a scan rate of 200 °C/h, followed by a second similar gradient, generating a reference thermogram. The data were acquired on “Low” feedback mode. The supplied software MicroCal PEAQ-DSC Software 1.53 was used to fit the experimental data. To obtain the final thermograms, each experiment had its reference thermogram subtracted, and the “Progress” baseline fitting method was used. The profile obtained was fitted with a “NonTwoState” model, accounting for two thermal events. Each experiment was performed in duplicates, and their averages were calculated by the software.

### Saturation Transfer Difference—NMR

^1^H STD-NMR spectra were acquired at 283 K on a Bruker AVANCE 600 MHz spectrometer. The protein and ligand were dissolved in phosphate buffer (Na_2_HPO_4_, KH_2_PO_4_) 20 mM pH 7.4, 100 mM NaCl, and 5% D_2_O in a 3 mm NMR tube (160 μL). Ligand/protein ratios were adjusted to 1000:1 in molar concentration. Water suppression was achieved by using the WATERGATE 3-9-19 pulse sequence. The on-resonance irradiation of the protein was kept at −0.05 and 10 ppm. Off-resonance irradiation was applied at 200 ppm, where no protein signals were visible. Selective pre-saturation of the protein was achieved by a train of Gauss-shaped pulses of 49 ms length each. The experiments were acquired with a saturation time of 2.94 s.

### Crystallization, Data Collection, and Structure Determination

Crystals of rBC2L-CN2 were obtained by 2 μL hanging drops and vapor diffusion using 1.2–1.3 M trisodium citrate at pH 7.0 at 19 °C and the protein at 5 mg/mL in 20 mM Tris–HCl pH 7.0 and 100 mM NaCl, as previously described.^25^ Cocrystals with H-type 1 tetrasaccharide were soaked overnight with 1.25 mM of compound **22a** (stock at 50 mM in protein buffer). Apo crystals were soaked for 5 h with 2 mM of compound **8c** (stock at 50 mM in 100% DMSO). The crystals were then cryoprotected using 2.5 M sodium malonate at pH 5.0 and flash-cooled in liquid nitrogen. Data for BC2L-C-Nt in complex with compounds **22a** and **8c** were collected at the synchrotron SOLEIL, Saint Aubin, France, on beamline Proxima 2 using an Eiger-9 M detector (Dectris, Baden, Switzerland) and on beamline Proxima 1 using an Eiger-16 M detector, respectively (see statistics in Table S4). Data were processed using XDS and XDSME, and then programs of the CCP4 suite were used.^36−38^ The coordinates of protomer A of PDB-ID 2WQ4 were used as a search model to solve all new structures of rBC2L-CN2 by molecular replacement using PHASER.^39^ Refinement was performed by multiple iterations of restrained maximum likelihood refinement and REFMAC 5.8 and manual rebuilding in Coot.^40,41^ 5% of the observations were set aside for cross-validation analysis. Hydrogen atoms were added in their riding positions during refinement. A library for the synthetic molecules was created in the Coot ligand builder. The final model was validated using the wwPDB validation server, https://validate-rcsb-1.wwpdb.org/, and the carbohydrate conformations were checked using Privateer.^42^ The coordinates were deposited in the Protein Data Bank (PDB) under codes 7OLU and 7OLW for structures in complex with **22a** and **8c**, respectively.

## Results and Discussion

### Design of Fucoside BC2L-C-Nt Antagonists

**Figure 1:**
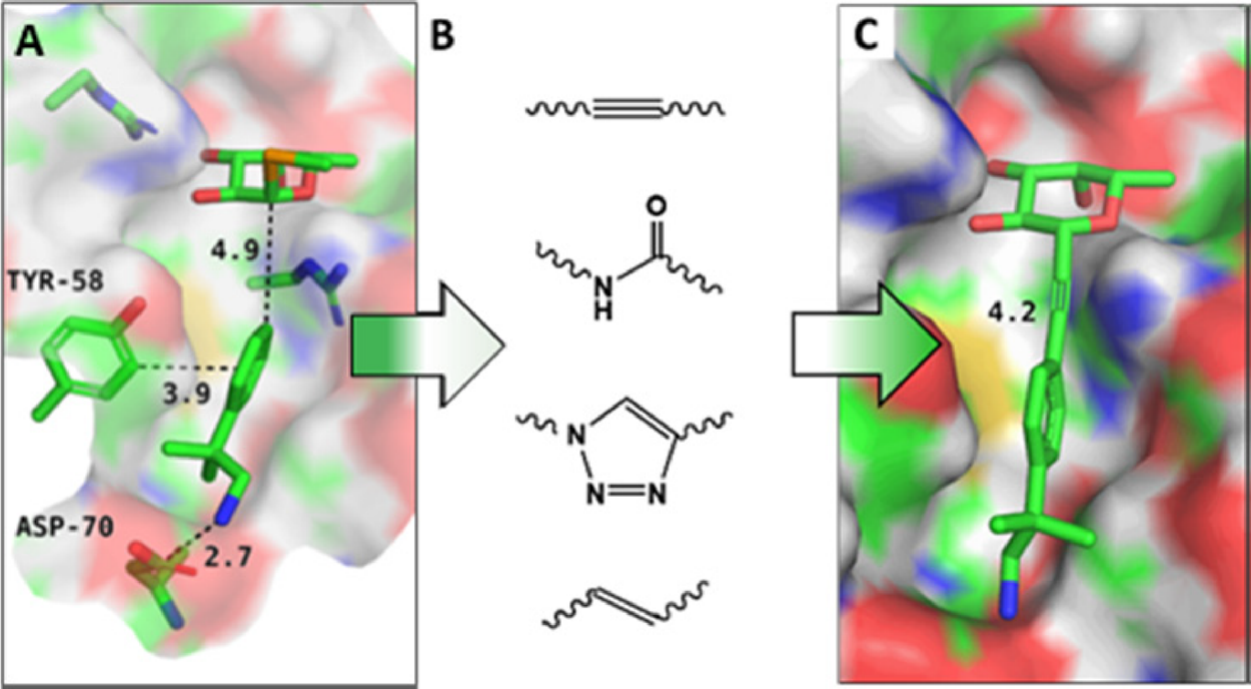
Ligand design strategy: (A) hit from fragment screening, docked in the presence of α-methylselenyl-fucoside, (B) chemical linkages considered, (C) corresponding designed BC2L-C-Nt antagonist is docked, encompassing both sites. Distances (Å, black) from anomeric carbon to closest fragment atom.

We previously described the *in silico* study of BC2L-C-Nt leading to the detection of a “ligandable” site, which consists of a crevice near the lectin’s fucose-binding site (Figure 1A). This area is not occupied by the native oligosaccharide ligands, which extend from the α-face of the fucose ring, but is located at the interface of two protomers in the BC2L-C-Nt trimer. Virtual screening of a fragment library in the BC2L-C-Nt complex with α-methylselenyl-fucoside (PDB 2WQ4) resulted in the selection of molecular fragments predicted to bind the vicinal site, which were validated by biophysical techniques.^26^ Selected fragments are used, here, for the design of new bifunctional molecules, generated by connecting the fragment to a fucoside core. Generally, the fragments employed are constituted by an aromatic moiety, predicted to interact in the newly detected binding site through edge-to-face interactions with residue Tyr58. Additionally, some fragments contain a terminal amino group predicted by the virtual screening to participate in an ionic or polar interaction with residue Asp70 at the bottom of the crevice (Figure 1A). The structures of the fragment moieties used can be deduced from Table 1. Their full structure and additional comments on their selection are collected in Section S2.1 and Figure S1.

**Table 1:**
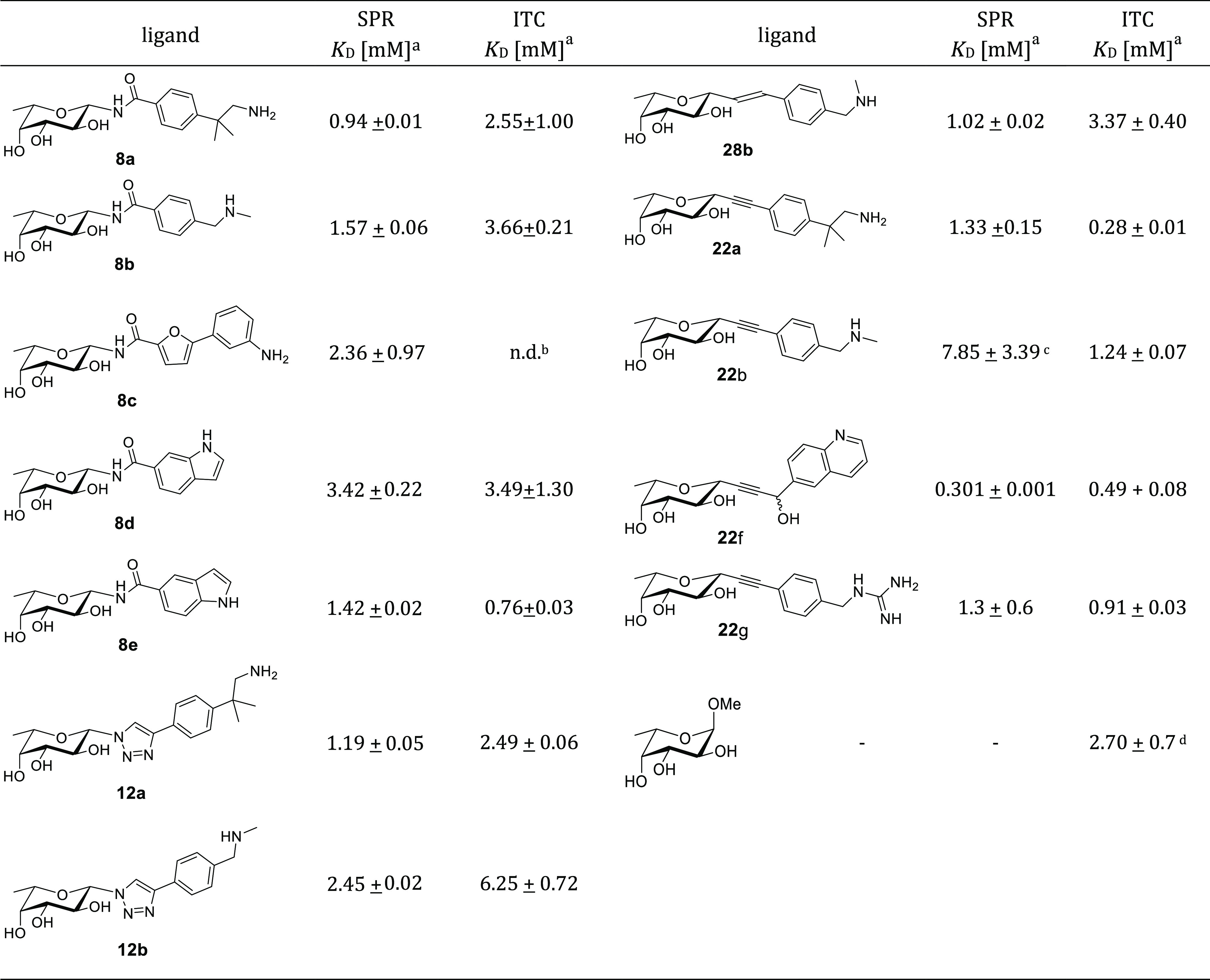
Panel of β-*N*- and β-*C*-Fucosides Synthesized; Affinity Evaluation by SPR and ITC

a Standard deviations from duplicates.

b Could not be determined due to low solubility of **8c**.

c Aspecific interaction with SPR chip observed.

d From Šulák and co-workers.^23^

Figure 1 describes the ligand design strategy: (A) the hits from fragment screening were found in close proximity (4–6 Å) to the fucoside’s anomeric position, in the direction of the β-substituent. This provided a clear synthetic strategy involving functionalization of the anomeric carbon, which was selected as the linking point. We note again here that the natural oligosaccharide ligands of the histo blood group, such as Globo H, are all α-fucosides. (B) A panel of connectors were considered to link the fragments to the anomeric carbon, generating (C) β-fucosides capable of engaging both the sugar-binding site and its neighboring site.

Concerning the linker, a single “fit-all” function was not possible, as the structures, orientations, and distances to the anomeric position varied from fragment to fragment.^26^ Instead, a set of linkers were explored, with different characteristics in terms of bridging length, angle, flexibility, bulkiness, polarity, and metabolic stability. Among these, the alkyne function was particularly interesting as a connector: a β-fucosylalkyne had an appropriate orientation and acceptable length and could be accessed through versatile chemistry. Alternatively, an amide bond was more easily synthesized and offered polar surfaces to interact with a nearby crystallographic conserved water molecule. Other linkages that seemed viable at this point for some of the fragments were a *E*-alkene bond and a 1,4-triazole ring (Figure 1B). To validate the design, the bifunctional ligands were screened *in silico* by docking, as described in Section S2. The first generation of antagonists was thus designed as a panel of β-*C*- and β-*N*-fucoside glycomimetic bifunctional molecules, simultaneously targeting BC2L-C-Nt’s carbohydrate binding site and its neighboring site (Figure 1C). Although the designed set of antagonists originally encompassed many more linker + fragment combinations, synthetic feasibility granted access to only a subset of those structures, listed in the next section (Table 1).

### Modular Synthesis of β-*C*- and β-*N*-Fucosides

**Scheme 1:**
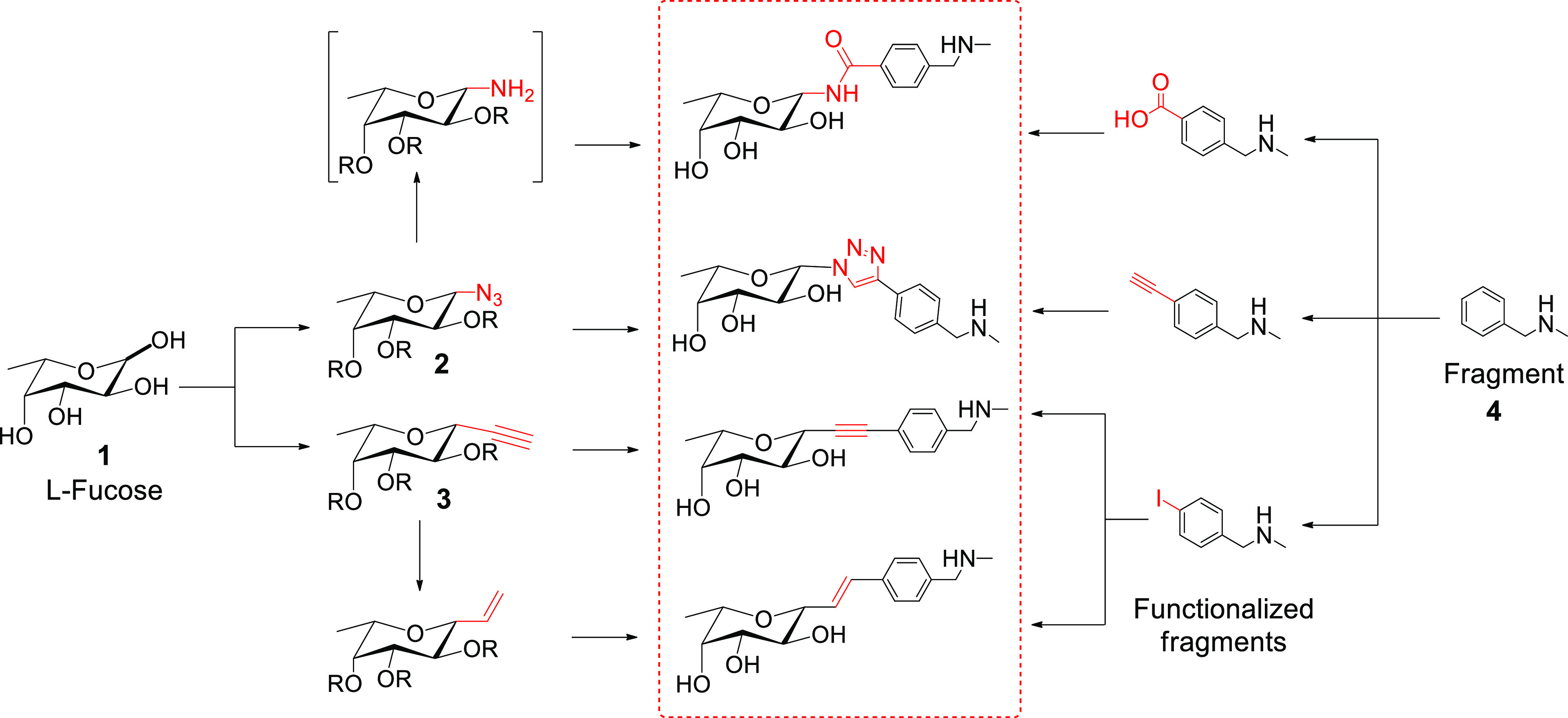
Modular Synthesis toward β-*C*- and β-*N*-Fucosides Exemplified for Fragment **4**

The synthetic route toward the designed fucosides was drafted to satisfy two requirements: (1) to be modular, allowing for all final molecules to be synthesized from the same building blocks; (2) to feature robust and reliable coupling procedures, in order to accommodate a range of fragment structures that could be expanded in the future. Scheme 1 summarizes how this approach was implemented starting from fucose through two key intermediates, the β-azidofucoside **2**, and the β-fucosylacetylene **3**. They could be coupled either directly or after minimal manipulation to the appropriately functionalized fragments (exemplified by **4** and its derivatives in Scheme 1), affording the full set of designed connections.

**Scheme 2:**
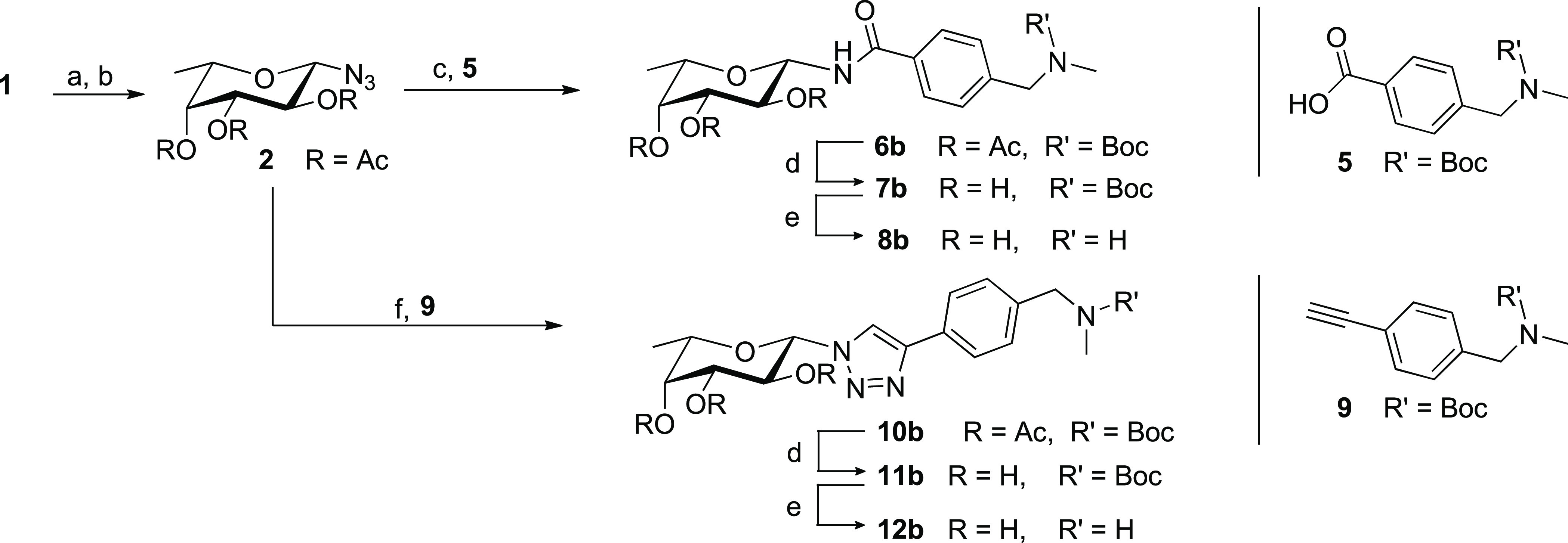
Synthesis of β-*N*-Fucosides Exemplified for One Fragment^*a*^ Reagents and conditions: (a) Ac_2_O, Pyr, rt.; (b) TMS-N_3_, SnCl_4_, DCM, 0 °C, 67% (over 2 steps); (c) PMe_3_, DCM, rt.; then: carboxylic acid **5**, HATU, DIPEA, DCM, rt., 45%; **or:** H_2_, Pd/C, MeOH, rt.; then: carboxylic acid **5**, HATU, DIPEA, DCM, rt.; (d) MeONa, MeOH, rt., 83%; **or:** NH_2_Me, EtOH, rt.; (e) TFA, DCM, 0 °C, quant.; (f) CuSO_4_·H_2_O, Na-Ascorbate, alkyne **9**, MeOH, rt., quant.

In detail, acetylation of L-fucose **1** and reaction with TMSN_3_ promoted by SnCl_4_ provided the azido intermediate **2** in good yields (67% over two steps) and selectivity (α/β ratio 9:91) (Scheme 2). Coupling of **2** with carboxylic partners, exemplified by **5** in Scheme 2, under Staudinger conditions, followed by protecting group removal steps as needed, led to amides **8a**–**e** (Table 1). Reaction of **2** with alkyne partners, such as **9**, under CuAAC conditions (Scheme 2) afforded triazole-linked bifunctional molecules that were deprotected giving **12a**–**b** (Table 1).

**Scheme 3:**
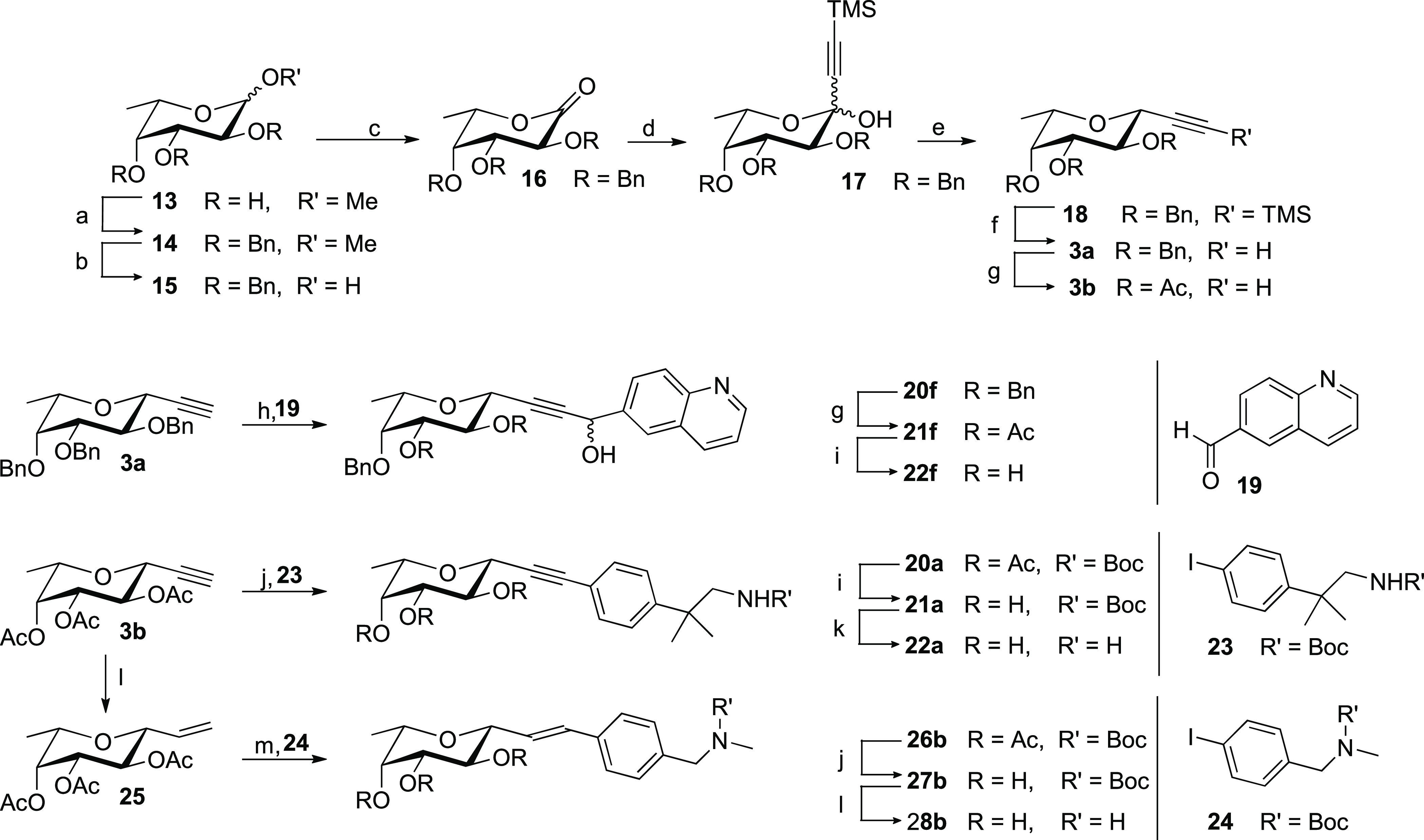
Synthesis of β-*C*-Fucosides Exemplified for Three Fragments^a^ Reagents and conditions: (a) BnBr, KOH, Tol, 111 °C, 80%; (b) HCl, AcOH, 118 °C, 78%; (c) I_2_, K_2_CO_3_, DCM, rt., 75%; (d) TMS-acetylene, nBuLi, CeCl_3_, THF, −78 °C, 87%; (e) Et_3_SiH, BF_3_·Et_2_O, CH_3_CN/DCM, −10 °C, 86%; (f) NaOH, MeOH/DCM, rt., 99%; (g) TMSOTf, Ac_2_O, rt., 61%; (h) LDA, aldehyde 19, THF, −20 °C, 72%; (i) MeONa, MeOH, rt.; (j) Sonogashira: Pd(PPh_3_)_4_, CuI, fragment 23, Piperidine, 80 °C; (k) TFA, DCM, 0 °C; (l) Lindlar’s Pd catalyst, H_2_, MeOH, 89%; (m) Heck: Pd(OAc)_2_, KCl, TBAB, K_2_CO_3_, AgNO_3_, fragment 24, DMF, 100 °C, 81%. Omitted yields are reported in Table S3.

The key β-fucosylacetylene **3** was synthesized adapting a methodology established for β-galactosylacetylenes (Scheme 3).^43,44^ This slightly adapted route is, to the best of our knowledge, the first report of stereoselective synthesis of β-fucosylacetylene as a building block. Starting from methyl α-L-fucopyranoside **13**, a protection/deprotection scheme (**13**–**15** in Scheme 3) followed by oxidation of the anomeric carbon (I_2_, 75%) led to the fuconolactone **16**. An organocerium reaction was used to install the acetylene moiety, resulting in **17** (87%) as an anomeric mixture, which was deoxygenated (Et_3_SiH, BF_3_·Et_2_O) to afford the β-fucosyl-trimethylsilylacetylene **18** with complete stereoselectivity. TMS removal under basic conditions (NaOH) produced the terminal alkyne **3a**. A permutation of protecting groups by acetolysis provided **3b**.

The *O*-benzyl intermediate **3a** was used to generate bifunctional molecules featuring a propargylic alcohol moiety. Indeed, some of the fragment screening hits bore a hydroxyl group directed toward the binding site and predicted to replace a crystallographic conserved water molecule.^26^ Entropically speaking, successfully replacing an ordered water molecule while maintaining its interactions can translate into a considerable affinity gain. Consequently, we aimed to validate this synthetic route with at least one structure (Scheme 3). Starting from **3a**, an organolithium reaction mediated by LDA allowed nucleophilic attack of the fucosylalkyne onto the aldehyde-bearing fragment **19**, resulting in **20f**. This reaction afforded a 1:1 mixture of stereoisomers, as observed by ^1^H-NMR spectroscopy of the crude reaction mixture (600 MHz). Acetolysis and de-acetylation afforded the deprotected diastereomeric mixture **22f** (81% over two steps), which could not be chromatographically resolved and was tested as such.

The alkyne **3b** was used for Sonogashira coupling with iodinated fragments, as exemplified by **23** and **24** in Scheme 3 to afford alkynes **20a** and **20b** (94 and 85%, respectively). Subsequent deprotections led to bifunctional ligands **22a** and **22b** (quantitative yields, see Table S3). The guanidine containing ligand **22g** (Table 1) was obtained by Goodman guanidinylation of **20b**,^45^ as described in the Supporting Information. Additionally, **3b** was transformed in the corresponding alkene **25** (Lindlar’s Pd catalyst), which underwent Heck coupling [Pd(OAc)_2_, KCl, TBAB, K_2_CO_3_, AgNO_3_, DMF, 100 °C] with **24** to selectively afford the *E*-product **26b**. Subsequent deprotections afforded the final alkene **28b** (quantitative yield, see Table S3).

To recap, synthetic routes toward the β-fucosylazide **2** and the β-fucosylacetylene **3** intermediates were validated. Coupling these intermediates to appropriately functionalized fragments led to four families of *N*- or *C*-fucosides. Thus, a modular synthesis framework allowing for rapid and stereoselective synthesis of β-*C-* and β-*N*-fucosides was drafted and validated, resulting in a panel of potential BC2L-C-Nt ligands to be screened against the protein target. Table 1 collects the molecules synthesized through this framework: amides **8a–e**, triazoles **12a–b**, alkynes **22a,b,f,g**, and alkene **28b**.

The remaining part of the work involved the synthesis of the functionalized fragments, which is detailed in Section S1.3.

### Biophysical Evaluation of the Glycomimetics

**Figure 2:**
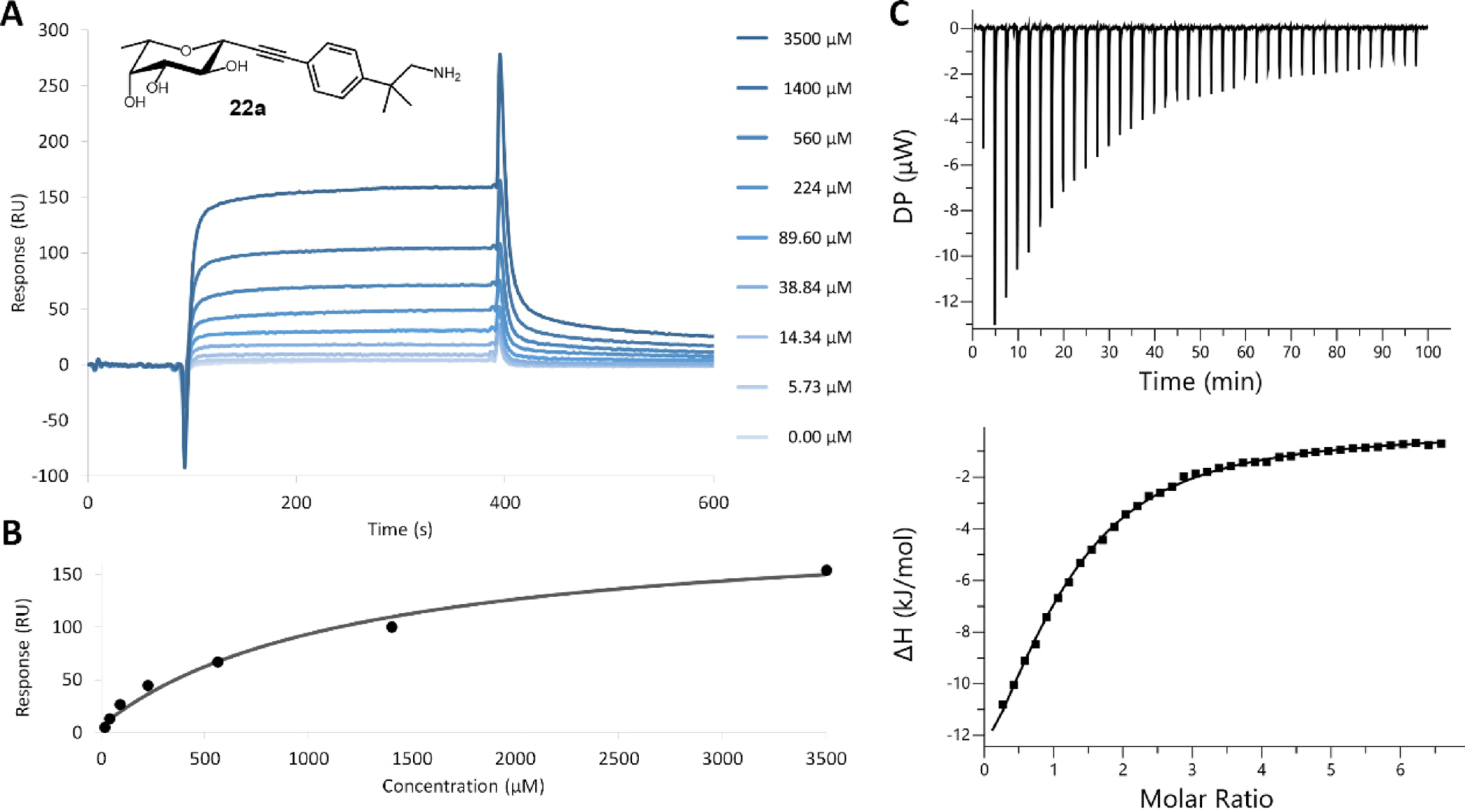
ITC and SPR experiments: (A, B) SPR analysis of **22a** binding to BC2L-C-Nt, multi-cycle sensogram and affinity analysis. (C) ITC titration of BC2L-C-Nt by **22a** (stoichiometry fitted to *N* = 1).

In order to evaluate the affinity of the newly synthesized structures for their target BC2L-C-Nt, surface plasmon resonance (SPR) and isothermal titration calorimetry (ITC) were employed. SPR evaluation of the synthetic structures against a BC2L-C-Nt-coated chip resulted in low millimolar affinities (Table 1, Figure 2A,B). These values are comparable with the affinity established for αMeFuc (ITC: 2.7 mM).^23^ With *K*_D_ values in the range of [0.3–3.4 mM], most ligands could be considered equivalent. Nevertheless, some of the better-performing compounds on SPR produced key results in ITC (**22a**, **22f–g**) or crystallography (**8c**). Additional limitations in the SPR experiments included the non-specific interactions observed between molecule **22b** and the chip, which explains the outlying affinity and big standard deviation recorded (7.85 + 3.39 mM). Moreover, a degree of variability was observed for the values measured on different protein chips. Thus, SPR allowed for a material-economic early ligand screening, resulting in a preliminary ranking of structures and in the identification of outliers and potential hits, fit for further assaying with different techniques.

ITC evaluation returned *K*_D_ values spread in a wider range than SPR: [0.28–6.25 mM]. This allowed a better sense of which compounds performed worse than the natural monosaccharide and which could become hits (Table 1). Of the evaluated panel, one amide (**8e**) and all four alkynes (**22a–g**) displayed a better activity than methyl-fucoside **13**. In particular, alkyne **22f** with a *K*_D_ of 490 μM confirmed the good affinity observed by SPR. However, this molecule was tested as a mixture of inseparable stereoisomers, and further studies are required to obtain a stereoselective synthesis and assess the activity of the single epimers. Alternatively, **22a** with a *K*_D_ of 281 μM was identified as the main hit with suitable values in both SPR and ITC (see Figure 2C) and a nearly 10-fold affinity increase from αMeFuc. Importantly, this simple ligand is also only one order of magnitude less active than the Globo H hexasaccharide (Fucα1-2Galβ1-3GalNAcβ1-3Galα1-4Galβ1-4Glc), which is the strongest known natural ligand of BC2L-C-Nt (*K*_D_ 26.05 μM ± 1.7 by ITC).^24^ This promising result validates the ligand design by fragment screening, as well as the choice of the acetylene moiety as a linker. Finally, the very low solubility of amide **8c** prevented affinity evaluation by ITC. However, both **22a** and **8c** went on to provide crystal structures in complex with the protein (see the following section).

**Figure 3:**
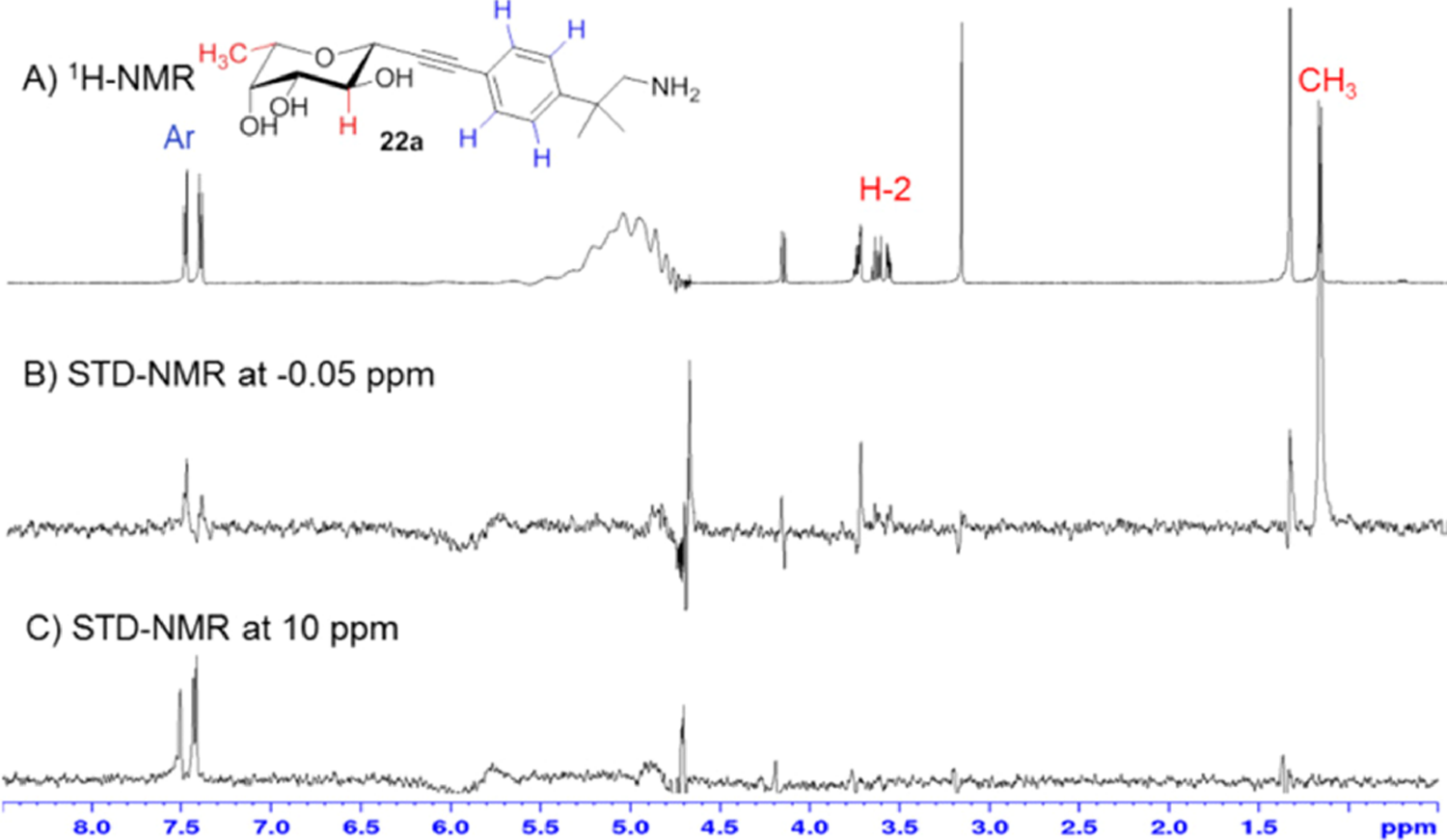
^1^H-NMR (A) and saturation transfer difference NMR experiments for **22a**, acquired using −0.05 ppm (for the irradiation of the aliphatic residues of the protein, (B) and 10 ppm (for the irradiation of aromatic amino acids, (C). The signals of aromatic protons are highlighted in blue and the protons of fucose in red.

Saturation transfer difference NMR (STD-NMR) experiments allowed us to further characterize the interaction between BC2L-C-Nt and hit **22a** (Figure 3A–C). STD data were used both to map the ligand epitope and to obtain information about the residues of the protein which are in contact with the ligand. This was obtained by examining the difference in ligand epitope maps observed when STDs are acquired at different saturating frequencies. Accordingly, two STD experiments were performed, irradiating either aliphatic (−0.05 ppm, Figure 3B) or aromatic (10 ppm, Figure 3C) residues of the protein. When the aliphatic residues were irradiated (Figure 3B), the strongest signal was observed for the methyl group in position 6 of the fucoside (1.26 ppm), indicating close contact to the protein, as observed for the monosaccharide in earlier work.^26^ Weaker STD signals were also observed for the H2 of the fucoside ring (3.74 ppm) and the other protons of the fucoside ring. This effect is expected, since the interactions between the fucoside moiety and the protein are mediated by H-bonds and the spectrum was obtained in D_2_O: deuterium exchange reduces the transfer of saturation, and ligand protons contacting polar residues will show a relative lower STD intensity compared to the interactions mediated by hydrophobic contacts.^46^ Also, the aromatic protons of the fragment moiety (7.44, 7.52 ppm) were involved in the epitope when the aliphatic residues were irradiated (Figure 3B), but produced a much stronger signal upon irradiation at 10 ppm (Figure 3C), hinting at a close contact to aromatic residues of the protein.

These results confirmed that **22a** adopts the known fucoside binding mode, in which the main protons exposed to the protein belong to C2 and C6. This, added to the aromatic signals observed for the fragment moiety, supports the expected binding mode of the ligand as designed, which was indeed later confirmed by crystallography (see below, Figure 5).

**Figure 4:**
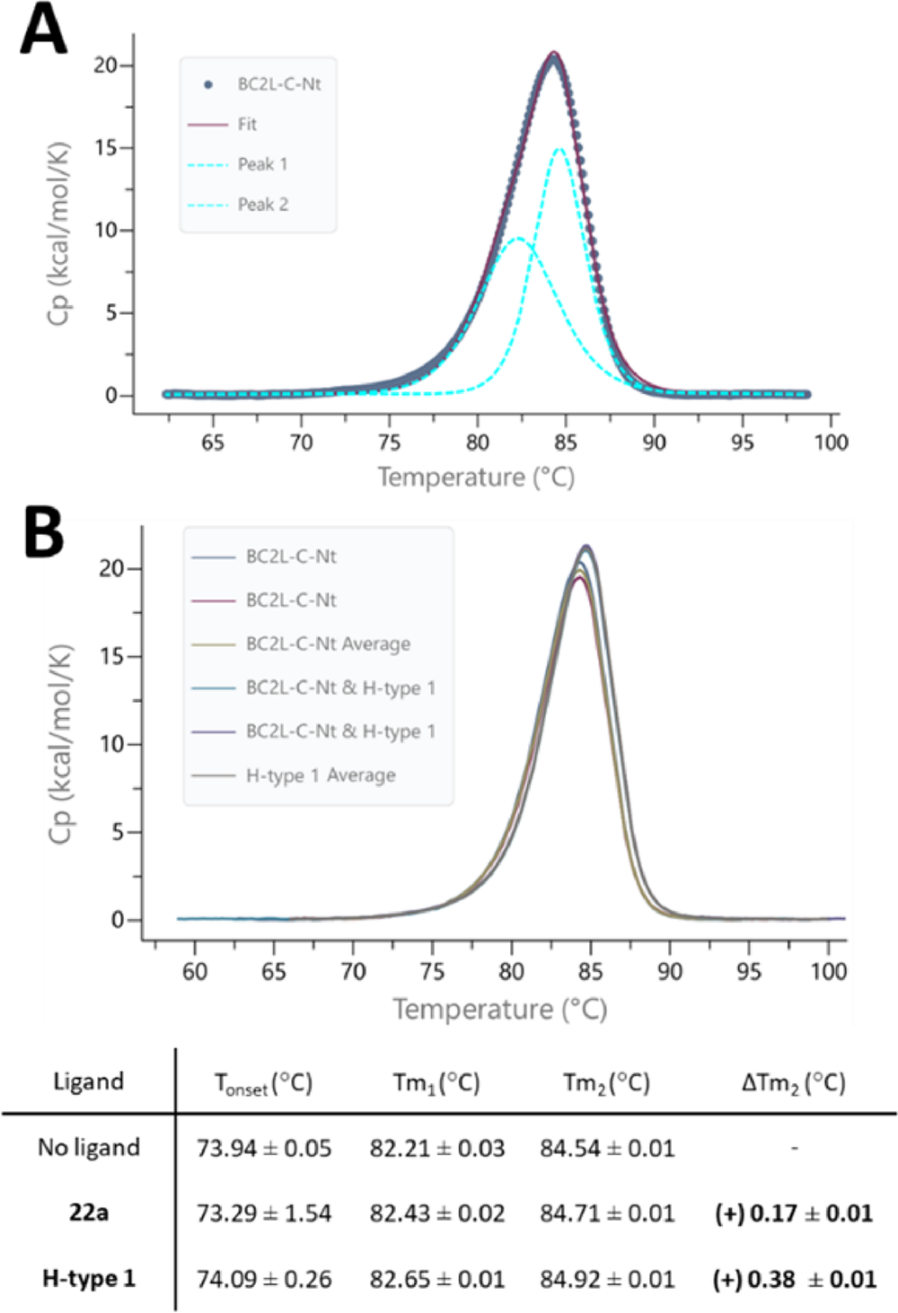
Differential scanning calorimetry: (A) Fitting with two thermal events. (B) Representative experiment comparing presence and absence of ligand H-type 1. Standard deviations from duplicates.

Since both the fucoside binding site and its vicinal site are located at the interface between monomers, it was interesting to evaluate the effect of the binding event on protein stability. For this, differential scanning calorimetry (DSC) experiments were performed. First, a reference experiment allowed us to define the protein’s unfolding profile upon a rising temperature gradient (Figure 4A). Thus, the experimental data were modeled into two sequential thermal events fitted as peaks *T*_m1_ and *T*_m2_, which could be attributed to the separation of monomers followed by their unfolding. The melting temperatures recorded were *T*_m1_ = 82.2 °C and *T*_m2_ = 84.5 °C. The addition of ligands to the protein could either increase (stabilizing) or decrease (destabilizing) these melting temperatures. Although stabilization of the protein is usually observed for complexes, destabilization would be observed if the synthetic ligand **22a** disrupts the protomeric interface. For comparison, the same experiment was performed for a known oligosaccharide ligand, the H-type 1 trisaccharide (Fucα(1–2)Galβ(1–3)GlcNAc), which binds to BC2L-C-Nt with micromolar affinity.^25^ The results obtained are summarized in Figure 4. As predicted for a stabilized complex, the H-type 1 trisaccharide provided a positive Δ*T*_m2_ value of +0.38 °C for the main thermal event (Figure 4B). To a lesser degree, synthetic ligand **22a** also stabilized the protein with a Δ*T*_m2_ value of +0.17 °C. The first thermal event was similarly shifted for both ligands (Δ*T*_m1_ = 0.44 and 0.21, respectively). These results confirm no detrimental effect to the stability of the BC2L-C-Nt trimer by either type of ligand.

### Structures of BC2L-C-Nt in Complex with Antagonists

**Figure 5:**
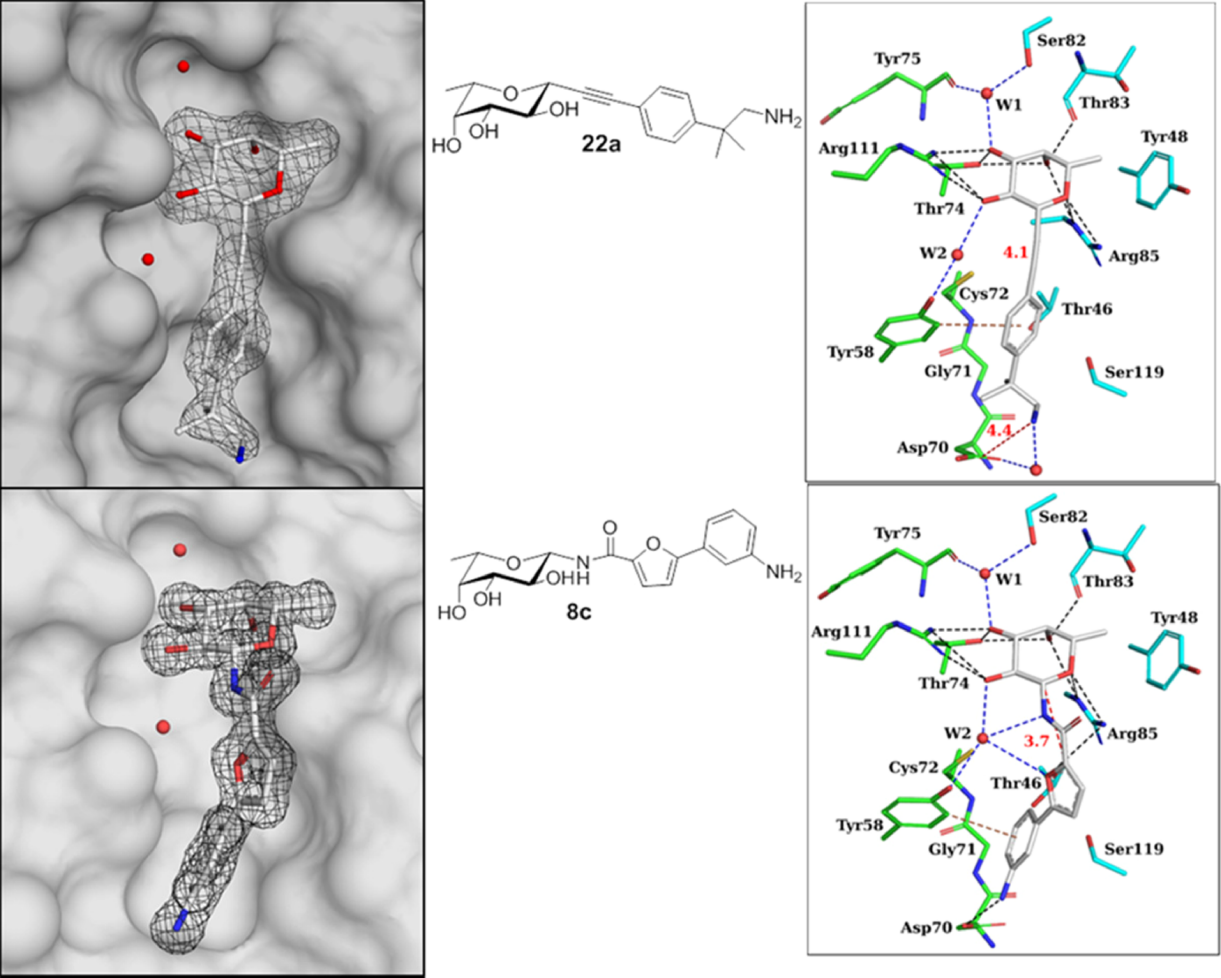
Left: Electronic density for synthetic ligands **22a** (top, 1.79 Å) and **8c** (bottom, 1.32 Å) in complex with BC2L-C-Nt. Water molecules **W1** and **W2** are depicted as red spheres, protein surface in transparent gray. Right: Deposited crystal structures (PDB: 7OLU, top and 7OLW, bottom). Direct protein/ligand interactions are shown in black (hydrophobic) or brown (edge-to-face π/π); water-mediated contacts are shown in blue. Distances (Å) from anomeric carbon to fragment or from amino group to Asp70 carboxylic centroid are depicted in red.

We solved two new crystal structures featuring complexes of BC2L-C-Nt with synthetic ligands **22a** and **8c**, allowing further rationalization of the affinity observed. The structures resulted from soaking BC2L-C-Nt crystals for 24 h in a 1.25 mM solution of each ligand and were solved to 1.79 and 1.32 Å resolution by molecular replacement. The relevant statistics can be found in Section S3 and Table S4. Inspection of the protein/ligand interactions as seen in Figure 5 confirmed the known fucoside binding mode from prior crystal structures: in both complexes, the sugar moiety establishes H-bonds with residues Thr74, Thr83, Arg85, and Arg111, as well as water-mediated contacts with Tyr75, Ser82, and Tyr58 (crystallographic waters **W1** and **W2**, respectively). A hydrophobic interaction between the C6 methyl group of the fucose moiety and the aromatic ring of Tyr48 is also observed.^23,25^

In the structure featuring ligand **22a**, the alkyne linker is 4.1 Å long and does not significantly dislocate the nearby crystallographic water **W2** (Figure 5, top). The fragment’s aromatic moiety engages in the predicted π/π T-shaped interaction with Tyr58, with an edge-to-face distance of 3.9 Å between its centroid and the closest C-atom of Tyr58 side chain, albeit the angle is 58°, rather than 90° (Figure 5, top). Lastly, the salt bridge between amino group and Asp70 carboxyl side chain predicted in the docked pose of the corresponding fragment (Section S2 and Figure S2B) is not observed. Possibly because the alkyne linker is shorter than the optimal fucose–fragment distance suggested by docking (4.1 Å vs 4.9 Å), thus locating the fragment moiety closer to the monosaccharide and further away from Asp70. Instead of the salt bridge predicted by docking, a water-mediated contact between Asp70 and the amino group is observed (Figure 5, top). Other characteristics of this interaction include the shape complementarity of hydrophobic patches: both methyl groups of the fragment are in close proximity with the otherwise exposed hydrophobic surface generated by the main chain of Gly71 and side chain of Tyr58. This hydrophobic complementarity carries on to match with residues Cys72, Thr46, and Ser119.

In the complex with ligand **8c**, the ligand features an amide linker, positioning the fragment moiety 3.7 Å away from the anomeric carbon (Figure 5, bottom). Designed to either replace or interact with crystallographic water **W2**, the amide bond interacts through its nitrogen atom, while the carbonyl points toward the solvent. The experimental structure perfectly matches the previously generated docking pose, including a π/π T-shaped interaction with Tyr58 (3.6 Å) and H-bonding interactions between the side chain of Asp70 and the aniline moiety. The furan moiety is located within the H-bonding distance to water **W2**, as predicted (Section S2 and Figure S3). In terms of shape complementarity, this ligand is more solvent exposed than the former, except for its aniline moiety, which matches the aforementioned hydrophobic patch composed of Gly71, Tyr58, and Thr46.

It is worth noting that in previous BC2L-C-Nt structures, at least two water molecules consistently resided in the space now filled by the fragment moieties, with variable positions across the different crystal structures. Release to the bulk of these loosely bound water molecules may translate into entropic gains. Altogether, the data presented confirm the compatibility of β-oriented substituents and the known fucoside binding mode. The alkyne and amide linkers are appropriate for this design. The structures also validate the binding poses predicted for the ligand or fragment structures (Section S3 and Figures S2 and S3), with the length of the linker being a limit for the latter. Additionally, we can rationalize the affinity gain observed for hit structure **22a** as the result of three factors: (1) the T-shaped π/π interaction, (2) the shape complementarity between hydrophobic surfaces, and (3) the thermodynamically advantageous entropic factor. Finally, the results with structure **8c** motivate the need to modulate its poor solubility in a future second generation design.

## Conclusions

We have developed a campaign of characterization and probing of a new biological target: the *superlectin* BC2L-C from *Burkholderia cenocepacia*. We have focused on the study of its *N*-terminal lectin domain and of the carbohydrate binding site it features. With the acquired data, we have designed the first generation of antagonists for this lectin, which consists of bifunctional β-*C*- or β-*N*-fucosides. Each of these bifunctional molecules bears a fragment moiety selected by *in silico* screening and aims to simultaneously target the sugar binding site and a vicinal region at the interface of two protomers. To ensure access to the designed structures, we conceived and validated a modular synthetic framework, which allows the straightforward synthesis of both β-*N*- and β-*C*-fucosides and can be widely applied for the synthesis of amide-, triazole-, alkyne-, and alkene-bound glycomimetics. Thus, we generated a panel of BC2L-C-Nt antagonists. The synthesized molecules were probed against their target by a range of techniques. In particular, STD-NMR and DSC showed definite responses, albeit with the low signal-to-noise ratio expected for the achieved affinity range. We envision that further experimentation with the next iteration of ligands will validate the potential of these techniques as early screens for future generations of antagonists. Finally, ITC allowed us to unambiguously claim two successful hits with improved affinity compared to the monosaccharide parent structure. The current leading antagonist **22a** presented a 10-fold affinity gain and validated our strategy, as well as the use of alkyne linkers in glycomimetic ligand design. In this context, it is important to stress that, while no general strategies exist for the rational design of glycomimetic lectin ligands, many of the reported hits contain a natural monosaccharide, meant to act as an anchor and to direct the ligand to the lectin carbohydrate recognition domain. Supplementary fragments capable of establishing additional interactions with the protein target in the vicinity of the carbohydrate binding site are then connected to the sugar core, possibly using non-glycosidic linkages. The nature of these fragments is often defined by trial and error. The work we report here provides experimental validation to earlier work^26^ describing the virtual screening of fragment libraries in the monosaccharide-lectin complex and thus shows that virtual screening of fragment libraries in the lectin complex of monosaccharides is an appropriate tool for fragment selection and rational design of glycomimetic structures.

Finally, we have solved the first crystal structures of BC2L-C-Nt complexes with synthetic ligands, validating our computational and experimental work so far, as well as the choice of amide linkers. Altogether, this campaign represents a first successful step in the search for viable antagonists of BC2L-C. On the one hand, a clear synthetic avenue leads to a class of validated structures. On the other hand, the campaign has allowed for preliminary SAR, which will be useful for the design of a second generation of antagonists.

## Supporting Information Available

Experimental procedures, ^1^H and ^13^C NMR spectra and characterization of the compounds, data collection, and refinement statistics (PDF)
