# Supplementary material for: Targeting a Multidrug-Resistant Pathogen: First Generation Antagonists of Burkholderia cenocepacia’s BC2L-C Lectin
Source: ACS Chem Biol. 2022 Sep 29;17(10):2899–910. doi: 10.1021/acschembio.2c00532 (PMC9594048; doi:10.1021/acschembio.2c00532)

Supporting Information

Targeting a Multidrug-Resistant Pathogen: first generation antagonists of *Burkholderia cenocepacia*'s BC2L-C lectin

Rafael Bermeo†‡, Kanhaya Lal‡†, Davide Ruggeri‡, Daniele Lanaro‡, Sarah Mazzotta‡, Francesca Vasile‡, Anne Imberty†, Laura Belvisi‡, Annabelle Varrot†*, Anna Bernardi‡*

† Univ. Grenoble Alpes, CNRS, CERMAV, 38000 Grenoble, France

‡ Università degli Studi di Milano, Dipartimento di Chimica, via Golgi 19, 20133 Milano, Italy

* Corresponding authors: anna.bernardi@unimi.it, annabelle.varrot@cermav.cnrs.fr

Contents

1. Synthetic procedures and chemical characterization S2
   1.1. General S2
   1.2. Synthesis of key intermediates for β-C-Fucosides: intermediates **3a-b** and **25** S3
   1.3. Synthesis of the functionalized fragments S6
   1.4. Synthesis of amides **6a-e** from azide **2** S11
   1.5. Synthesis of triazoles **10a-b** from azide **2** S13
   1.6. Synthesis of alkynes **20a-b** by Sonogashira coupling from **3b** S15
   1.7. Synthesis of 1-(quinolin-6-yl)-3-(β-L-fucopyranosyl)prop-2-yn-1-ol **21f** from **3a** S16
   1.8. Synthesis of **26b** by Heck reaction S17
   1.9. Removal of protecting groups and synthesis of ligands **8a-e**, **12a-b**, **28b**, **22a-f** S18
      1.9.1. General procedure **A** for deacetylation (Zemplén deacetylation) S18
      1.9.2. General procedure **B** for deacetylation (deacetylation with NH_2Me,) S18
      1.9.3 General procedure **C** for Boc-removal S18
   1.10. Syntesis of the guanidine derivative **22g** S24
2. Ligand design S26
   2.1. Ligand structure and selection S26
   2.2. Virtual screening of fragments and glycomimetic ligands S26
   2.3. Protein preparation S26
   2.4. Ligand preparation S27
   2.5. Receptor grid for docking study S27
   2.6. Ligand docking S27
3. Ligand evaluation materials and methods S29

3.1. Isothermal Titration Calorimetry S29
3.2. Surface plasmon Resonance S29
3.3. Differential Scanning Calorimetry S29
3.4. Saturation Transfer Difference - NMR S30
3.5. Crystallization, Data Collection, and Structure Determination S30
4. References S32
5. NMR spectra S35

1. Synthetic procedures and chemical characterization

1.1. General

Chemicals were purchased from commercial sources and used without further purification, unless otherwise indicated. When anhydrous conditions were required, the reactions were performed under nitrogen or argon atmosphere. Anhydrous solvents were purchased from Sigma-Aldrich® with a content of water ≤0.005%. Triethylamine (Et_3N), methanol and dichloromethane were dried over calcium hydride, THF was dried over sodium/benzophenone and freshly distilled. N,N-Dimethylformamide (DMF) was dried over 4 Å molecular sieves. Washing solutions used in the work-up procedures were saturated, unless stated otherwise. Reactions were monitored by analytical thin-layer chromatography (TLC) performed on Silica Gel 60 F_{254} plates (Merck) and TLC Silica gel 60 RP-18 F_{254}s (Merck) were analysed with UV detection (254 nm and 365 nm) and/or staining with: ammonium molybdate acid solution, potassium permanganate alkaline solution, ninhydrin stain, Dragendorff stain. Flash column chromatography was performed using silica gel 60 (40–63 μm, Merck). Automated flash chromatography was performed with Biotage Isolera Prime system and SNAP ULTRA cartridges were employed. For HPLC purifications, a Waters 600 controller coupled to a Waters 2487 Dual Absorbance Detector (214 and 250 nm) were used at a flow rate of 22.0 mL/min (Varioprep column: 250/21 mm nucleosil 100-7 C_{18}). The gradient used was linear from H_2O (0.1% TFA) to CH_3CN 9/1 H_2O (0.1% TFA). NMR experiments were recorded on a Bruker AVANCE 400 MHz instrument at 298 K. Chemical shifts (δ) are reported in ppm. The ^1H and ^{13}C NMR resonances of compounds were assigned with the assistance of COSY and HSQC experiments. Multiplicities are assigned as s (singlet), d (doublet), t (triplet), q (quartet), m (multiplet), mult. (for multiplets encompassing more than one proton). Broad peaks are denoted by *b*. Aromatic and heteroaromatic protons and carbons are denoted as *Ar*, or *hAr* when ambiguous. The ^{13}C-NMR spectra are APT. Mass spectra were recorded on a Thermo Fischer LCQ apparatus (ESI ionization). High resolution mass spectra were recorded on spectrometers Apex II ICR FTMS (ESI ionization-HRMS) or Thermo Fischer LTQ Orbitrap XL (ESI ionization-HRMS) or from a VG AutoSpec M246 (Fisons) spectrometer equipped with EBE geometry and EI source (EI-HRMS). Specific optical rotation values were measured using either a Perkin-Elmer 241 or an ADP410 polarimeter at 589 nm with a 1.0 or 0.5 dm cell, respectively.

The β-fucosylazide **2** is a known compound and was prepared following the method of Palomo et al.[1] Methyl α-L-fucopyranoside **13** is commercially available and was purchased from Carbosynth.

1.2. Synthesis of key intermediates for β-C-Fucosides: intermediates 3a-b and 25

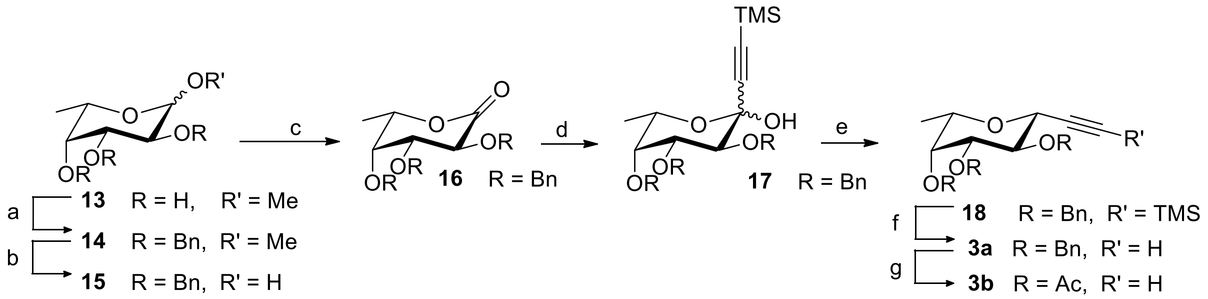

Scheme S1. [a]Reagents and conditions: **a.** BnBr, KOH, Tol, 111°C, 80%; **b.** HCl, AcOH, 118°C, 78%; **c.** I_2, K_2CO_3, CH_2Cl_2, rt, 75%; **d.** TMS-acetylene, nBuLi, $CeCl_3$, THF, -78°C, 87%; **e.** Et_3SiH, $BF_3{\cdot}Et_2O$, CH_3CN/ CH_2Cl_2, -10°C, 86%; **f.** NaOH, MeOH/ CH_2Cl_2, rt, 99%; **g.** TMSOTf, Ac_2O, rt, 61%.

1.2.1. Synthesis of **methyl 2,3,4-tri-*O*-benzyl L-fucopyranoside 14**

To a solution of **methyl α-L-fucopyranoside 13** (1.0 g, 5.61 mmol, 1 eq) in dry Toluene (30 mL) BnBr (8.0 mL, 67.26 mmol, 12 eq) was added, followed by ground KOH (3.15 g, 5.14 mmol, 10 eq). The reaction mixture was heated to reflux (111 °C) under N_2 atmosphere and stirred for 43 h, before returning to room temperature. The mixture was quenched with ice-cold water and extracted with EtOAc. The organic phase was washed with ice-cold water and dried over $MgSO_4$. The crude was purified by automatic chromatography (Biotage SNAP 100: nHex/EtOAc 5 % isocratic, then gradient to 50 %) affording product **14** (2.02 g, 4.51 mmol, y = 80 %) as an oil. TLC R_f (nHex/EtOAc: 7/3): 0.64. MS (ESI) calculated for $C_{28}H_{32}O_5$ $[M + Na]^+$ *m/z*: 471.21; found: 471.41. ^{1}H NMR (400 MHz, $CDCl_3$): δ = 7.41 - 7.26 (mult., 15H, *Ar*), 5.00-4.65 (mult., 6H, *CH_2-OBn*), 4.66 (d, $J_{1\text{-}2}$= 3.6 Hz, 1H, *H-1*), 4.04 (dd, $J_{2\text{-}1}$ = 3.6 Hz, $J_{2\text{-}3}$ = 10.1 Hz, 1H, *H-2*), 3.93 (dd, $J_{3\text{-}2}$ = 10.1 Hz, $J_{3\text{-}4}$ = 2.8 Hz, 1H, *H-3*), 3.84 (dq, $J_{5\text{-}4}$ = 1.3 Hz, $J_{5\text{-}CH3}$ = 6.5, 1H, *H-5*), 3.64 (dd, $J_{4\text{-}3}$ = 2.9 Hz, $J_{4\text{-}5}$ = 1.3 Hz, 1H, *H-4*), 3.36 (s, 3H, *OCH_3*), 1.12 (d, $J_{CH3\text{-}5}$ = 6.5 Hz, 3H, *CH_3*). In accordance with published data.[2] ^{13}C chemical shifts were extrapolated from the HSQC experiment: δ = 128.3-127.4 (*CH Ar*), 98.7 (*C-1*), 79.5 (*C-3*), 77.9 *C-4*), 76.4 (*C-2*), 74.8-73.4 (*CH_2-OBn*), 66.1 (*C-5*), 55.3 (*OCH_3*), 16.6 (*CH_3*).

1.2.2. Synthesis of **2,3,4-tri-*O*-benzyl L-fucopyranose 15** following the procedure of Nishi and co-workers [2]

To a solution of **methyl 2,3,4-tri-*O*-benzyl L-fucopyranoside 14** (5.6 g, 12.48 mmol, 1 eq) in acetic acid (100 mL) was added 1M HCl (25 mL, 25 mmol, 2.0 eq). The reaction mixture was heated to reflux (118 °C) and stirred until TLC showed completion (3 h), before returning to room temperature. The mixture was quenched with ice-cold water and extracted with CH_2Cl_2. The organic phase was washed with $NaHCO_3$ aqueous solution and brine, then dried over Na_2SO_4. Recrystallization was achieved by suspending the crude in a Hex/Et_2O solution (40/13 mL: *ca.* 110 mg/mL), dissolving by heating to 45 °C, cooling to -16 °C, then filtering white crystals of the anomeric mixture **15** (4.24 g, 9.76 mmol, y = 78 %). TLC R_f (nHex/EtOAc: 7/3): 0.34. ^{1}H NMR (400 MHz, $CDCl_3$): anomeric mixture α/β (ratio 2:1)α-anomer: δ = 7.36 - 7.27 (mult., 15H, *Ar*), 5.26 (d, $J_{1\text{-}2}$= 3.6 Hz, 1H, *H-1*), 5.01-4.65 (mult., 6H, *CH_2-OBn*), 4.10 (q, $J_{5\text{-}CH3}$ = 6.5 Hz, 1H, *H-5*), 4.04 (dd, $J_{2\text{-}1}$=3.7 Hz, $J_{2\text{-}3}$=9.9 Hz, 1H, *H-2*), 3.90 (dd, $J_{3\text{-}2}$ = 9.9 Hz, $J_{3\text{-}4}$ = 2.8 Hz, 1H, *H-3*), 3.67 (dd, $J_{4\text{-}3}$ = 2.8 Hz, $J_{4\text{-}5}$ = 1.1 Hz, 1H, *H-4*), 2.89 (bs, 1H, *OH*), 1.14 (d, $J_{CH3\text{-}5}$ = 6.5 Hz, 3H, *CH_3*). In accordance with published data.[2] β-

anomer: δ = 7.36 - 7.27 (mult., 15H, *Ar*), 5.01-4.65 (mult., 6H, *CH_2-OBn*), 4.63 (d, 1H, *H-1*), 3.74 (t, 1H, *H-2*), 3.59 (d, 1H, *H-4*), 3.55 (mult., 2H, *H-3* + *H-5*), 3.08 (bs, 1H, *OH*), 1.20 (d, 3H, *CH_3*).

1.2.3. Synthesis of **2,3,4-tri-*O*-benzyl L-fuconolactone 16** following the procedure of Fusaro and co-workers[3]

To a solution of **2,3,4-tri-*O*-benzyl L-fucopyranoside 15** (4.08 g, 9.39 mmol, 1 eq) in CH_2Cl_2 (60 mL), I_2 (8.33 g, 32.82 mmol, 3.5 eq) was added, followed by ground K_2CO_3 (4.54 g, 32.85 mmol, 3.5 eq). The brown reaction mixture was stirred at room temperature for 23 h until TLC showed completion, before being quenched with ice-cold water and extracted with CH_2Cl_2. The organic phase was washed with $Na_2S_2O_3$ aqueous solution, becoming clear, and with brine and then dried over Na_2SO_4. The crude was purified by automatic chromatography (Biotage SNAP 100: nHex/EtOAc gradient from 2% to 30%) affording product **16** (3.00 g, 6.94 mmol, y = 74 %). TLC R_f (nHex/EtOAc: 7/3): 0.64. ^{1}H NMR (400 MHz, $CDCl_3$): δ = 7.44 - 7.31 (mult., 15H, *Ar*), 5.21 (d, J= 11.0 Hz, 1H, *CH_2-OBn*), 4.98 (d, J'= 11.5 Hz, 1H, *CH_2-OBn*), 4.80 (m, J'''= 11.9 Hz, J'= 11.0 Hz, 2H, *CH_2-OBn*), 4.69 (m, J'''= 12.0 Hz, J'= 11.5 Hz, 2H, *CH_2-OBn*), 4.47 (d, J_{2-3}= 9.6 Hz, 1H, *H-2*), 4.33 (dq, J_{5-4} = 1.5 Hz, J_{5-CH3} = 6.5 Hz, 1H, *H-5*), 3.89 (dd, J_{3-2} = 9.6 Hz, J_{3-4} = 2.2 Hz, 1H, *H-3*), 3.80 (dd, J_{4-3} = 2.3 Hz, J_{4-5} = 1.6 Hz, 1H, *H-4*), 1.34 (d, J_{CH3-5} = 6.5 Hz, 3H, *CH_3*). In agreement with published data[4]

1.2.4. Synthesis of **(1-hydroxy-2,3,4-tri-*O*-benzyl-L-fucopyranosyl) trimethylsilyl acetylene 17** following the procedure of Lowary and co-workers [5]

Oven-dried glassware was used for this procedure. $CeCl_3$ (1.230 g, 4.99 mmol, 2.6 eq) was ground and heated (120 - 140 °C) under high vacuum for 35min. After returning to room temperature, it was flushed with Argon, cooled to 0 °C, and suspended in freshly distilled THF (14 mL). The flask was cooled to -78 °C and left to stir for 2h until the next addition. Simultaneously, a flask under Ar atmosphere was cooled to -78 °C before adding a 0.9 M solution of TMS-acetylene in dry THF (8.5 mL, 7.65 mmol, 4.0 eq), then a 2.5 M solution of n-BuLi in hexanes (3.4 mL, 8.5 mmol, 4.4 eq). The reaction mixture was stirred at -78 °C for 1h45 before being added to the -78 °C $CeCl_3$ suspension via cannula. The opaque white reaction suspension was stirred at -78 °C for 50 min until the next addition. Simultaneously, **2,3,4-tri-*O*-benzyl L-fuconolactone 16** (830 mg, 1.92 mmol, 1 eq) was flushed with Argon, cooled to -78 °C, and dissolved in freshly distilled THF (14 mL). The solution was then added to the -78 °C reaction mixture via cannula, producing a slight peach coloration in the opaque white suspension. The resulting reaction mixture was stirred for 2 h at -78 °C then was allowed to return to room temperature. The reaction was quenched with a 0.1 M HCl aqueous solution and extracted with EtOAc. The organic phase was washed with brine, then dried over Na_2SO_4. The crude was purified by automatic chromatography (Biotage SNAP 100: nHex/tBuOMe gradient from 2% to 40%), separating anomer products **17** (135 mg, 0.25 mmol, y = 87%). TLC R_f (nHex/tBuOMe: 7/3): 0.15 (**16**), 0.30 (**17**). ^{1}H NMR (400 MHz, $CDCl_3$): major-anomer: δ = 7.43 - 7.24 (mult., 15H, *Ar*), 5.03 - 4.68 (mult., 6H, *CH_2-OBn*), 4.15 (d, J_{2-3}= 9.8 Hz, 1H, *H-2*), 4.07 (dq, J_{5-4} = 1.3 Hz, J_{5-CH3} = 6.5 Hz, 1H, *H-5*), 3.78 (dd, J_{3-2} = 9.7 Hz, J_{3-4} = 2.9 Hz, 1H, *H-3*), 3.62 (dd, J_{4-3} = 2.9 Hz, J_{4-5} = 1.3 Hz, 1H, *H-4*), 3.27 (bs, 1H, *OH*), 1.22 (d, J_{CH3-5} = 6.5 Hz, 3H, *CH_3*), 0.21 - 0.18 (m, 9H, *Si-CH_3*). minor-anomer: δ = 7.43 - 7.24 (mult., 15H, *Ar*), 5.03 - 4.38 (mult., 6H, *CH_2-OBn*), 3.88 (mult., J_{2-3}= 10.0 Hz, J_{5-4} = 1.2 Hz, J_{5-CH3} = 6.5 Hz, 2H, *H-2* + *H-5*), 3.69 (dd, J_{3-2} = 10.0 Hz, J_{3-4} = 2.9 Hz, 1H, *H-3*), 3.56 (dd, J_{4-3} = 2.9 Hz, J_{4-5} = 1.3 Hz, 1H, *H-4*), 3.18 (bs, 1H, *OH*), 1.24 (d, J_{CH3-5} = 6.5 Hz, 3H, *CH_3*), 0.21 - 0.18 (m, 9H, *Si-CH_3*). ^{13}C NMR (100 MHz, $CDCl_3$), some shifts were extrapolated from the HSQC experiment: major-anomer: δ = 138.7, 138.3 (*C Ar*), 128.6 - 127.7 (*CH Ar*), 104.4 (*C1*), 92.0 (*C-Si*), 88.5 (-*C*≡), 80.7 (*C2*), 80.0 (*C3*), 77.1 (*C4*), 76.3, 74.8, 73.3 (*CH_2 Ar*), 68.4 (*C5*), 17.0 (*C6*), -0.2

(CH_3-*Si*). minor-anomer: δ = 81.2 (*C2* + *C3*), 77.1 (*C4*), 70.6 (*C5*), 74.6, 74.3, 73.4 (CH_2 *Ar*), 17.0 (*C6*), -0.1 (CH_3-*Si*).

1.2.5. Synthesis of **(2,3,4-tri-*O*-benzyl β-L-fucopyranosyl) trimethylsilyl acetylene 18** following the procedure of Lowary and co-workers [5]

A solution of **1-hydroxy-2,3,4-tri-*O*-benzyl L-fucopyranosyl) trimethylsilyl acetylene 17** (213 mg, 0.40 mmol, 1 eq) dissolved in CH_3CN/ CH_2Cl_2 (ratio 2:1 - 5.35 mL, 2.67 mL) was cooled to -10 °C under Ar atmosphere. Et_3SiH (256 μL, 1.61 mmol, 4.0 eq), then $BF_3{\cdot}Et_2O$ (248 μL, 2.01 mmol, 5.0 eq) were added to the solution and left to stir at -10 °C for 1h, before returning to room temperature. The reaction mixture was quenched with a few drops of Et_3N and diluted in EtOAc. The organic phase was washed with water and brine, then was dried over Na_2SO_4. The crude product was purified by automatic chromatography (Biotage SNAP 10: nHex/tBuOMe from 0 % to 50 %) affording product **18** (178 mg, 0.35 mmol, y = 86 %). TLC R_f (nHex/tBuOMe: 7/3): 0.70. ^{1}H NMR (400 MHz, $CDCl_3$): δ = 7.40 - 7.29 (mult., 15H, *Ar*), 5.02 - 4.99 (m, 2H, CH_2-*OBn*), 4.87 (d, 1H, CH_2-*OBn*), 4.81 - 4.70 (mult., 3H, CH_2-*OBn*), 4.02 - 4.00 (mult., 2H, *H-1* + *H-2*), 3.60 (dd, $J_{4\text{-}3}$ = 2.9 Hz, $J_{4\text{-}5}$ = 1.1 Hz, 1H, *H-4*), 3.49 (m, 1H, *H-3*), 3.45 (dq, $J_{5\text{-}4}$ = 1.1 Hz, $J_{5\text{-}CH3}$ = 6.4 Hz, 1H, *H-5*), 1.21 (d, $J_{CH3\text{-}5}$ = 6.4 Hz, 3H, CH_3), 0.18 (m, 9H, *Si*-CH_3). ^{13}C NMR (100 MHz, $CDCl_3$), some shifts were extrapolated from the HSQC experiment: δ = 138.6, 138.5 (*C Ar*), 128.6 – 127.6 (*CH Ar*), 103.0 (-*C*≡), 90.4 (*C-Si*), 83.8 (*C3*), 79.1 (*C2*), 76.8 (*C4*), 75.8, 74.9, 73.1 (CH_2 *Ar*), 74.9 (*C5*), 70.7 (*C1*), 17.4 (*C6*), -0.1 (CH_3-*Si*).

1.2.6. Synthesis of **(2,3,4-tri-*O*-benzyl β-L-fucopyranosyl) acetylene 3a**, following the procedure of Dondoni and co-workers [6]

To a solution of **(2,3,4-tri-*O*-benzyl β-L-fucopyranosyl) trimethylsilyl acetylene 18** (122 mg, 0.24 mmol, 1 eq) dissolved in MeOH/CH_2Cl_2 (ratio 5:1 - 7.9 mL) was added a 1M solution of NaOH (415 μL, 42 mmol, 1.75 eq). The reaction mixture was stirred at room temperature for 40 min until TLC showed completion, before it was quenched with a few drops of a 2 N HCl solution. The solvents were evaporated and the resulting crude was dissolved in water and extracted with EtOAc. The organic phase was washed with water and dried over Na_2SO_4. The crude product was purified by flash chromatography (nHex/tBuOMe 7:3) affording product **3a** (104 mg, 0.24 mmol, y = 99 %). TLC R_f (nHex/tBuOMe: 7/3): 0.54. $[\alpha]_D^{19}$ = -2.5 ($CHCl_3$, c 0.9). ^{1}H NMR (400 MHz, $CDCl_3$): δ = 7.42 - 7.28 (mult., 15H, *Ar*), 5.03 - 4.98 (m, 2H, CH_2-*OBn*), 4.90 (d, 1H, CH_2-*OBn*), 4.81 - 4.70 (mult., 3H, CH_2-*OBn*), 4.02 (mult., 2H, *H-1* + *H-2*), 3.63 (dd, $J_{4\text{-}3}$ = 2.9 Hz, $J_{4\text{-}5}$ = 1.1 Hz, 1H, *H-4*), 3.52 (dd, $J_{3\text{-}2}$ = 8.4 Hz, $J_{3\text{-}4}$ = 2.9 Hz, 1H, *H-3*), 3.48 (dq, $J_{5\text{-}4}$ = 1.1 Hz, $J_{5\text{-}CH3}$ = 6.4 Hz, 1H, *H-5*), 2.51 (d, J = 1.90 Hz, ≡*CH*), 1.22 (d, $J_{CH3\text{-}5}$ = 6.4 Hz, 3H, CH_3). ^{13}C NMR (100 MHz, $CDCl_3$), some shifts were extrapolated from the HSQC experiment: δ = 138.6, 138.5, 138.4 (*C Ar*), 128.6 – 127.7 (*CH Ar*), 83.9 (*C3*), 81.5 (-*C*≡), 78.8 (*C2*), 76.7 (*C4*), 75.8, 74.8, 73.0 (CH_2 *Ar*), 74.9 (*C5*), 73.7 (≡*CH*), 70.0 (*C1*), 17.4 (*C6*).

1.2.7. Synthesis of **(2,3,4-tri-*O*-acetyl β-L-fucopyranosyl) acetylene 3b** following the procedure of Alzeer and co-workers [7]

To a solution of **(2,3,4-tri-*O*-benzyl β-L-fucopyranosyl) acetylene 3a** (200 mg, 0.45 mmol, 1 eq) dissolved in Ac_2O (9 mL) TMSOTf (0.73 mL, 4.05 mmol, 9 eq) was added under Ar atmosphere. The brown reaction mixture was stirred at room temperature for 24h until TLC showed completion, before it was diluted with water and carefully quenched with a saturated aqueous solution of $NaHCO_3$. The mixture was extracted with EtOAc, washed with $NaHCO_3$ (3x), water and brine, then dried over

Na_2SO_4. The crude product was purified by automatic chromatography (Biotage SNAP 100: nHex/EtOAc from 5 % to 70 %) affording product **3b** (100 mg, y = 76%). TLC R_f (nHex/EtOAc: 7/3): 0.31. $[\alpha]_D^{19}$ = -30 ($CHCl_3$, c 1). MS (ESI) calculated for $C_{14}H_{18}O_7$ $[M + H]^+$ *m/z*: 299.11; found: 299.05. ^1H NMR (400 MHz, $CDCl_3$): δ = 5.38 (dd, , $J_{2\text{-}1}$ = $J_{2\text{-}3}$ = 10.0 Hz, 1H, *H-2*), 5.26 (dd, $J_{4\text{-}3}$ = 3.4 Hz, $J_{4\text{-}5}$ = 1.2 Hz, 1H, *H-4*), 4.99 (dd, $J_{3\text{-}2}$ = 10.2 Hz, $J_{3\text{-}4}$ = 3.4 Hz, 1H, *H-3*), 4.15 (dd, $J_{1\text{-}2}$ = 9.9 Hz, J_{alkyne} = 2.2 Hz, 1H, *H-1*), 3.79 (dq, $J_{5\text{-}4}$ = 1.2 Hz, $J_{5\text{-}CH3}$ = 6.4 Hz, 1H, *H-5*), 2.48 (d, J_{alkyne} = 2.2 Hz, 1H, *≡CH*), 2.18 (s, 3H, *OAc*), 2.07 (s, 3H, *OAc*), 1.98 (s, 3H, *OAc*), 1.21 (d, $J_{CH3\text{-}5}$ = 6.4 Hz, 3H, CH_3). ^{13}C NMR (100 MHz, $CDCl_3$), some shifts were extrapolated from the HSQC experiment: δ = 170.9, 170.4, 169.7 (*C=O*), 78.6 (*-C≡*), 75.0 (*≡CH*), 73.6 (*C5*), 72.1 (*C3*), 70.6 (*C4*), 68.9 (*C1*), 68.6 (*C2*), 21.0, 20.9, 20.9 (*CH_3 OAc*), 16.6 (*C6*).

1.2.8. Synthesis of **(2,3,4-tri-*O*-acetyl β-L-fucopyranosyl) ethene 25** following the procedure of Rouzier and co-workers [8]

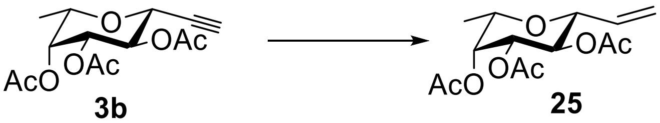

3b **25**

To a solution of **(2,3,4-tri-*O*-acetyl β-L-fucopyranosyl) acetylene 3b** (54 mg, 0.181 mmol, 1 eq) dissolved in MeOH (1.8 mL) was added Lindlar's catalyst (4mg, 0.019mmol, 0.2 eq). The mixture was put under H_2 atmosphere (1atm) and stirred a room temperature for 3 h before filtering and concentrating. The crude product **25** (48 mg, 0.160 mmol, y = 89%) was used for the next step without further purification. TLC R_f (nHex/EtOAc: 7/3): 0.45. MS (ESI) calculated for $C_{14}H_{20}O_7$ $[M + Na]^+$ *m/z*: 323.11; found: 323.27. ^1H NMR (400 MHz, $CDCl_3$): δ = 5.78 (ddd, J_{trans} = 17.5 Hz, J_{cis} = 10.4 Hz, $J_{CH\text{-}1}$ = 7.4 Hz, 1H, *<u>CH</u>=CH_2*), 5.29 (dd, J_{trans} = 17.2 Hz, J_{gem} = 1.2 Hz, 2H, *CH=<u>CH_2</u>*), 5.27 (dd, $J_{4\text{-}3}$ = 3.4 Hz, $J_{4\text{-}5}$ = 1.2 Hz, 1H,*H-4*), 5.12 (dd, $J_{2\text{-}1}$ = 9.4 Hz, $J_{2\text{-}3}$ = 10.2 Hz, 1H, *H-2*), 5.05 (dd, $J_{3\text{-}2}$ = 10.2 Hz, $J_{3\text{-}4}$ = 3.4 Hz, 1H, *H-3*), 3.81 (mult., $J_{5\text{-}4}$ = 1.2 Hz, $J_{5\text{-}CH3}$ = 6.5 Hz, $J_{1\text{-}2}$ = 9.4 Hz, $J_{1\text{-}CH}$ = 7.4 Hz, 2H, *H-4* + *H-1*), 2.17 (s, 3H, *OAc*), 1.98 (s, 3H, *OAc*), 1.97 (s, 3H, *OAc*), 1.18 (d, $J_{CH3\text{-}5}$ = 6.5 Hz, 3H, CH_3). ^{13}C NMR (100 MHz, $CDCl_3$), some shifts were extrapolated from the HSQC experiment: δ = 170.8, 170.4, 169.9 (*C=O*), 134.0 (*<u>CH</u>=CH_2*), 119.9(*CH=<u>CH_2</u>*), 80.1 (*C1*), 72.8 (*C5*), 72.3 (*C3*), 71.0 (*C4*), 68.7 (*C2*), 21.0, 20.9, 20.8 (*CH_3 OAc*), 16.6 (*C6*).

1.3. Synthesis of the functionalized fragments

In order to achieve a modular approach to the synthesis of the fragments (**Scheme 1, Table S1**), we selected to use the aryl iodides **23** and **24** (**Scheme 3**) as common intermediates, from which the alkynes and carboxylic acids were synthesized as exemplified in **Scheme S2** for **24** and in **Scheme S3** for **23**.

Thus, the iodide **24** (**Scheme S2**) was synthesized starting from commercially available 4-iodio-benzylbromide **29** upon reaction with 33% w/w $MeNH_2$ in Et_2O solution, followed by Boc protection. This was found to be superior to iodination of N-methylbenzylamine, which was not fully regioselective. Sonogashira reaction of **24** with TMS-acetylene followed by TMS removal (NaOH) afforded the alkyne **9**, which could be converted in acid **5** by $KMnO_4$ oxidation.

The iodide **23** (**Scheme S3**) was synthesized starting from commercially available 4-iodiophenylacetonitrile **30** by double methylation (tBuONa, MeI, THF/NMP) and BH_3 reduction (BH_3·THF in THF) affording amine **31** which was Boc-protected (Boc_2O) to provide **23**. Sonogashira reaction of **23** with TMS-acetylene followed by TMS removal (TBAF) afforded the alkyne **32**, which could be converted in acid **33** by $KMnO_4$ oxidation.

The quinoline-6-carbaldehyde **19** was synthesized from commercially available 6-quinolinylmethanol **34** (Section 1.3.8.)

Table S1. Panel of the functionalized fragments used for the synthesis of the bifunctional ligands

	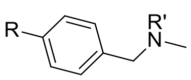	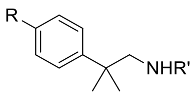	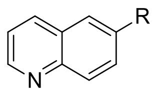
R = I<br>R' = H	-	**31**[b]	-
R = I<br>R' = Boc	**24**[a]	**23**	-
R = C≡CH<br>R' = Boc	**9**	**32**	-
R = COOH<br>R' = Boc	**5**	**33**	-
R=CH_2OH	-	-	**34**[c]
R=CHO	-	-	**19**

a. From commercially available **29** as described below; b. From commercially available **30**, as described below; c. Commercially available

All the other fragments used for the synthesis of amides **8c-e** were commercially available carboxylic acids (**Table S2**) and were purchased from abcr, Fluorochem or Thermo Fisher.

Scheme S2. Strategy for fragment functionalization. Synthesis of **24**, **9** and **5.**

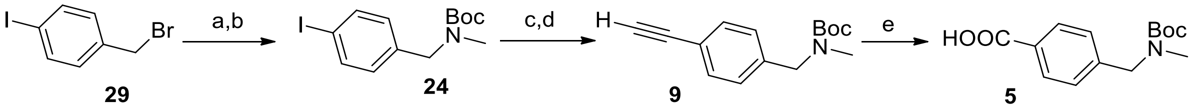

Reagents and conditions: **a.** $MeNH_2$ **b.** Boc_2O, CH_2Cl_2, rt; **c**. Sonogashira: TMS-acetylene, $Pd(PPh_3)_4$, CuI, NEt_3, Toluene, rt; **d.** NaOH, MeOH/ CH_2Cl_2, rt; **e**. $KMnO_4$, $NaHCO_3$, tBuOH/H_2O, rt. Yields reported in Table S3.

1.3.1. Synthesis of ***tert*-butyl (4-iodobenzyl)-*N*-(methyl)carbamate 24**

4-Iodobenzyl bromide 29 (2.0 g, 6.74 mmol, 1 eq) was dissolved in CH_2Cl_2 under N_2 atmosphere. The solution was transferred to a dropping funnel and added to a stirring $MeNH_2$ 33% w/w Et_2O solution (12.5 mL, 100 mmol, 14.8 eq) over 30 min. The reaction was stirred at room temperature for an additional 40 min until TLC showed completion, before concentrating. The crude was dissolved in CH_2Cl_2 and washed with a NaOH aqueous solution. The organic phase was dried over Na_2SO_4 and concentrated to afford the crude amine (1.56 g), used for the next step without purification. TLC R_f (nHex/EtOAc: 9/1): 0.60. ^{1}H NMR (400 MHz, $CDCl_3$): δ = 7.64 (d, J = 8.3 Hz, 2H, *CH-C-I*), 7.07 (d, J = 8.2 Hz, 2H, *CH Ar*), 3.69 (s, 2H, *CH_2*), 2.43 (s, 3H, *CH_3*).

Crude ***N*-methyl-(4-iodobenzyl)amine** (2.01 g, 8.14 mmol, 1 eq) was dissolved in CH_2Cl_2 (81 mL) and stirred at room temperature under N_2 atmosphere. A solution of Boc_2O (2.47 g, 11.32 mmol, 1.4 eq) in CH_2Cl_2 was added and the reaction mixture was stirred for 3h until TLC showed completion, before the solvent was concentrated. The crude product was purified by automatic chromatography (Biotage Sfär 100: nHex/EtOAc 10% isocratic) affording product **24** (2.293 g, 6.60 mmol, y = 81 % over 2 steps). TLC R_f (nHex/EtOAc: 9/1): 0.25. 1H NMR (400 MHz, CDCl3): δ = 7.65 (d, J = 8.2 Hz, 2H, CH-C-I), 6.97 (bd, 2H, CH Ar), 4.35 (s, 2H, CH2), 2.80 (bd, 3H, CH3), 1.47 (bs, 9H, tBu). In accordance with published data.[9] ^{13}C NMR shifts extrapolated from the HSQC experiment: δ = 137.6 (*CH-C-I Ar*), 129.4 (*CH Ar*), 51.7 (*CH_2*), 33.9 (*CH_3*), 28.4 (*tBu*).

1.3.2. Synthesis of ***tert*-butyl(4-((ethynyl)benzyl)-*N*-(methyl)carbamate 9** following the procedure of Decréau and co-workers[10]

***tert*-butyl (4-iodobenzyl)(methyl)carbamate 24** (197 mg, 0.57 mmol, 1 eq), $Pd(Ph_3)_4$ (24 mg, 0.02 mmol, 0.05 eq) and CuI (16 mg, 0.09 mmol, 0.2 eq) were dissolved in toluene (1.5 mL), under Ar atmosphere. TMS-acetylene (100 µL, 0.70 mmol, 1.2 eq) and Et_3N (100 µL, 0.72 mmol, 1.3 eq) were added and the reaction mixture was stirred at room temperature for 1h45 until TLC showed completion, before being concentrated. The crude was purified by automatic chromatography (Biotage Sfär 25: nHex/EtOAc gradient 0 % to 25 %) affording the TMS-alkyne (184 mg, quantitative yield). TLC R_f (nHex/EtOAc: 8/2): 0.70. MS (ESI) calculated for $C_{18}H_{27}NO_2Si$ $[M + Na]^+$ *m/z*: 340.17; found: 340.09. ^1H NMR (400 MHz, $CDCl_3$): δ = 7.42 (d, J = 8.3 Hz, 2H, *≡C-C-<u>CH</u> Ar*), 7.14 (d, J = 6.9 Hz, 2H, *CH Ar*), 4.39 (bs, 2H, *CH_2*), 2.83 - 2.76 (bd, 3H, *CH_3*), 1.47 (bs, 9H, *tBu*), 0.24 (s, 9H, Si-*CH_3*). ^{13}C NMR (100 MHz, $CDCl_3$), some shifts were extrapolated from the HSQC experiment: δ = 138.8 (*CH_2-<u>C</u> Ar*), 132.3 (*≡C-C-<u>CH</u> Ar*), 127.7 - 127.1 (*CH Ar*), 122.1 (*≡C-<u>C</u> Ar*), 105.0 (*≡<u>C</u>-C Ar*), 94.3 (*≡<u>C</u>-Si*), 52.7 - 51.9 (*CH_2*), 34.2 (*CH_3*), 28.6 (*tBu*), 0.1 (*Si-CH_3*).

To ***tert*-butyl methyl(4-((trimethylsilyl)ethynyl)benzyl)carbamate** (70 mg, 0.22 mmol, 1 eq) in THF (500 µL) was added a 1 M solution of TBAF in THF (250 µL, 0.25 mmol, 1.1 eq). The brown reaction mixture was stirred at room temperature for 10 min until TLC showed completion, before being concentrated. The crude was dissolved in CH_2Cl_2 and washed with a 1 M aqueous HCl solution. The organic phase was dried over Na_2SO_4 and concentrated to crude ***tert*-butyl methyl(4-((ethynyl)benzyl)carbamate 9** (55 mg, quantitative yield), used for the next step without purification. TLC R_f (nHex/EtOAc: 8/2): 0.56. MS (ESI) calculated for $C_{15}H_{19}NO_2$ $[M + Na]^+$ *m/z*: 268.13; found: 268.11. ^1H NMR (400 MHz, $CDCl_3$): δ = 7.46 (d, J = 8.1 Hz, 2H, *≡C-C-<u>CH</u> Ar*), 7.17 (d, J = 6.9 Hz, 2H, *CH Ar*), 4.41 (bs, 2H, *CH_2*), 3.06 (s, 1H, *≡CH*), 2.84 - 2.79 (bd, 3H, *CH_3*), 1.46 (bs, 9H, *tBu*). ^{13}C NMR (100 MHz, $CDCl_3$), some shifts were extrapolated from the HSQC experiment: δ = 132.3 (*≡C-C-<u>CH</u> Ar*), 127.8 - 127.2 (*CH Ar*), 121.1 (*≡C-<u>C</u> Ar*), 77.7 (*≡CH*), 52.2 (*CH_2*), 33.8 (*CH_3*), 28.6 (*tBu*).

1.3.3. Synthesis of **4-(((tert-butoxycarbonyl)(methyl)amino)methyl) benzoic acid 5**

***tert*-butyl (4-iodobenzyl)(methyl)carbamate 9** (82 mg, 0.33 mmol, 1 eq) was dissolved in tBuOH (11 mL). Separately, $KMnO_4$ (158 mg, 1.00 mmol, 3 eq) was dissolved in water (11 mL). Separately, $NaHCO_3$ (87 mg, 1.04 mmol, 3 eq) was dissolved in water (11 mL). The $NaHCO_3$ solution, followed by the $KMnO_4$ solution were added to the dissolved alkyne and stirred at 30 °C for 1 h until TLC showed completion. The reaction mixture was quenched by stirring with MeOH, then was filtered and concentrated. The crude was redissolved in CH_2Cl_2 and acidified water (to pH 2, using 1M HCl). The organic phase was dried over Na_2SO_4 and concentrated. The crude was purified by automatic chromatography (Biotage Sfär 10: nHex/EtOAc gradient 0 % to 60 %; 0.01% formic acid) affording **5** (43 mg, 0.16 mmol, y = 50 %). TLC R_f (nHex/EtOAc: 7/3): 0.73. MS (ESI) calculated for $C_{14}H_{19}NO_4$

[M + Na]$^+$ *m/z*: 288.12; found: 288.14. ^{1}H NMR (400 MHz, $CDCl_3$): δ = 8.08 (d, J = 8.2 Hz, 2H, O=*C-C-CH Ar*), 7.32 (d, J = 7.7 Hz, 2H, *CH Ar*), 4.49 (bs, 2H, *CH_2*), 2.89 - 2.83 (bd, 3H, *CH_3*), 1.46 (bs, 9H, *tBu*). ^{13}C NMR (100 MHz, $CDCl_3$): δ = 171.3 (*COOH*), 144.6 (*CH_2-C Ar*), 130.7 (O=*C-C-CH Ar*), 128.4 (O=*C-C Ar*), 127.7 - 127.2 (*CH Ar*), 80.2 (*C tBu*), 53.6 - 52.0 (*CH_2*), 34.4 (*CH_3*), 28.6 (*CH_3 tBu*).

Scheme S3. Strategy for fragment functionalization. Synthesis of **23**, **32** and **33**

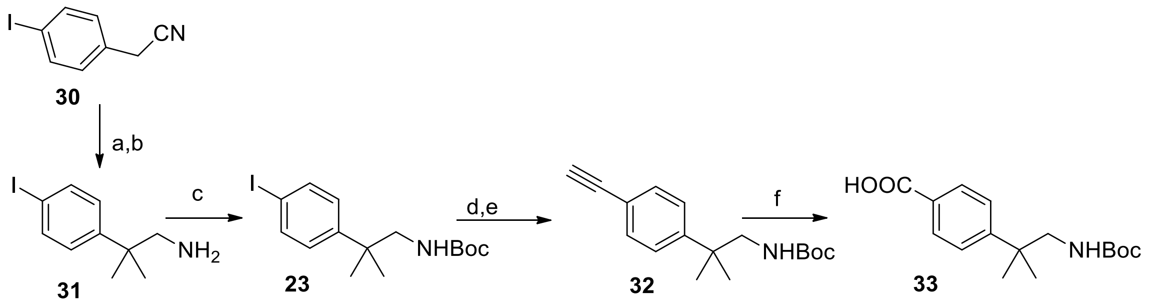

Reagents and conditions: **a**. tBuONa, MeI, THF/NMP; **b**. BH_3·THF in THF **c.** Boc_2O, CH_2Cl_2, rt; **d.** Sonogashira: $Pd(PPh_3)_4$, CuI, NEt_3, Toluene, rt; **e.** TBAF in THF, rt; **f.** $KMnO_4$, $NaHCO_3$, tBuOH/H_2O, rt. Yields reported in **Table S3**.

1.3.4. Synthesis of **2-(4-iodophenyl)-2-methylpropan-1-amine 31**

tBuONa (780 mg, 8.2 mmol, 4 eq) was suspended in dry THF/NMP (1:1, 4mL) and the mixture was cooled to 0 °C under nitrogen atmosphere. In another flask 4-iodophenylacetonitrile **30** (520 mg, 2.0 mmol, 1 eq) was dissolved in THF/NMP (1:1, 4mL) and MeI (500 µL, 8.2 mmol, 4 eq) was added. The solution was slowly added in the first flask (the temperature should not increase above 10 °C, final concentration 0.25 M). After the addition, the reaction was warmed to room temperature and stirred overnight until TLC revealed completion. The reaction was quenched with HCl 2M aqueous solution and extracted with toluene (3x). The combined organic phases were washed with $NaHCO_3$ saturated aqueous solution, brine and $NaHSO_3$. The organic layer was dried over Na_2SO_4 and concentrated under reduced pressure to afford the crude di-methylated species (630 mg, quantitative yield), used as such for the following step. TLC Rf (nHex/EtOAc: 92/8): 0.43. MS (ESI) calculated for $C_{10}H_{10}IN$ [M + Na]$^+$ m/z: 271.99; found 271.70. ^{1}H NMR (400 MHz, $CDCl_3$): δ = 7.74 (d, J = 8.4 Hz, 2H, *CH Ar*), 7.24 (d, J = 8.6 Hz, 2H, *CH Ar*), 1.72 (s, 6H, *CH_3*). ^{13}C NMR (400 MHz, $CDCl_3$): δ = 141.2 (*C Ar*), 138.0 (*CH Ar*), 127.1 (*CH Ar*), 124,4 (*C Ar*), 93.4 (*CN*), 36.9 (*C-2CH_3*) 29.0 (*CH_3*).

The crude (240 mg, 0.890 mmol, 1 eq) was dissolved in dry THF (2.67 mL) and the solution was cooled to 0°C. 1M BH_3· THF in THF (4.45 mL, 4.45 mmol, 5 eq) was added and the reaction mixture was refluxed for 24 h until completion. The solution was cooled to 0°C and aqueous 6M HCl was added, then the reaction was stirred for 30 min. After this time, 1M NaOH was added to the mixture until basic pH and the aqueous layer was extracted with CH_2Cl_2 (x3). The organic phase was washed with water, brine, and dried over Na_2SO_4. The solvent was evaporated under reduced pressure to afford the crude amine **31**, used without further purification (212 mg, y = 86%). TLC (CH_2Cl_2/MeOH: 9/1): 0.55. MS (ESI) calculated for $C_{10}H_{14}IN$ [M + Na]$^+$ m/z: 297.99; found 298.37. ^{1}H NMR (400 MHz, $CDCl_3$): δ = 7.65 (d, J= 8 Hz, 2H, *CH Ar*), 7.05 (d, J= 8 Hz, 2H, *CH Ar*), 2.77 (s, 2H, *CH_2*), 1.27 (s, 6H, *2x CH_3*). ^{13}C NMR shifts extrapolated from the HSQC experiment: δ = 137.4 (*CH Ar*), 128.4 (*CH Ar*), 51.7 (*CH_2*), 26.3 (*CH_3*).

1.3.5 Synthesis of ***tert*-butyl (2-(4-iodophenyl)-2-methylpropyl) carbamate 23**

The free amine **31** (210 mg, 0.76 mmol, 1 eq.) was dissolved in dry THF (7.6 mL) and then TEA (160 µL, 1.140 mmol, 1.5 eq) was added. After 5 min Boc_2O (248 mg, 1.140 mmol, 1.5 eq.) was added to the mixture and the reaction was stirred at room temperature under nitrogen atmosphere for 2 h. The reaction was washed with 1M HCl aqueous solution and water. The organic phase was dried over Na_2SO_4, filtered and concentred under reduced pressure. The crude product was purified by automatic chromatography (nHex : EtOAc, gradient from 0 to 20% in 10 CV) to afford the product **23** (180 mg, y = 63%). TLC Rf (nHex/EtOAc: 9/1): 0.37. MS (ESI) for $C_{15}H_{22}INO_2$ $[M + Na]^+$ *m/z*: 398.06 found 398.37. ^1H NMR (400 MHz, $CDCl_3$): δ = 7.63 (d, J = 8.5 Hz, 2H, *CH Ar*), 7.10 (d, J = 8.4 Hz, 2H, *CH Ar*), 4.28 (bs, 1H, *NH*), 3.28 (d, J = 6.3 Hz, 2H, *CH_2*), 1.38 (br s, 9H, *tBu*), 1.28 (s, 6H, *2xCH_3*). ^{13}C NMR shifts extrapolated from the HSQC experiment: δ = 137.4 (*CH Ar*), 128.4 (*CH Ar*), 51.7 (*CH_2*), 28.3 (*tBu*), 26.3 (*CH_3*).

1.3.6 Synthesis of ***tert*-butyl (2-(4-ethynylphenyl)-2-methylpropyl)carbamate 32**

Compound **23** (123 mg, 0.33 mmol, 1 eq), $Pd(Ph_3)_4$ (16 mg, 0.02 mmol, 0.05 eq) and CuI (13 mg, 0.07 mmol, 0.2 eq) were dissolved in toluene (850 µL), under Argon atmosphere. TMS-acetylene (60 µL, 0.42 mmol, 1.3 eq) and Et_3N (60 µL, 0.43 mmol, 1.3 eq) were added and the reaction mixture was stirred at room temperature until TLC showed completion (1h30), before being concentrated. The crude was purified by automatic chromatography (Biotage Sfär 25: nHex/EtOAc gradient 0 % to 20 %) affording the TMS-alkyne intermediate (87 mg, 0.25 mmol, y = 76 %). TLC R_f (nHex/EtOAc: 8/2): 0.67. MS (ESI) calculated for $C_{20}H_{31}NO_2Si$ $[M + Na]^+$ *m/z*: 368.20; found: 368.20. ^1H NMR (400 MHz, $CDCl_3$):δ = 7.43 (d, J = 8.4 Hz, 2H, *≡C-C-<u>CH</u> Ar*), 7.27 (mult., 4H, *CH Ar*), 4.22 (bs, 1H, *NH*), 3.30 (d, 2H, *CH_2*), 1.39 (bs, 9H, *tBu*), 1.28 (s, 6H, *CH_3*), , 0.24(s, 9H, *Si-CH_3*).^{13}C NMR shifts extrapolated from the HSQC experiment: δ = 132.0 (*<u>CH</u>-C-C≡ Ar*), 129.8, 129.6, 126.0 (*CH Ar*), 51.9 (*CH_2*), 28.3 (*tBu*), 26.3 (*CH_3*), 0.2 (Si-*CH_3*).

To a solution of the TMS-alkyne (60 mg, 0.17 mmol, 1 eq) in THF (500 µL), 1 M solution of TBAF in THF (200 µL, 0.20 mmol, 1.2 eq) was added. The reaction mixture was stirred at room temperature for 5 min until TLC showed completion, before being concentrated. The crude was dissolved in CH_2Cl_2 and washed with a 1 M HCl aqueous solution. The organic phase was dried over Na_2SO_4 and concentrated to obtain the crude compound **32** (43 mg, yield: 90 %), used for the next step without further purification. TLC R_f (nHex/EtOAc: 8/2): 0.59. MS (ESI) calculated for $C_{17}H_{23}NO_2$ $[M + Na]^+$ *m/z*: 296.16; found: 296.10.^1H NMR (400 MHz, $CDCl_3$): δ = 7.45 (d, J = 8.4 Hz, 2H, *<u>CH</u>-C-C≡ Ar*), 7.35 - 7.26 (mult., 5H, 2 *CH Ar*), 4.27 (bs, 1H, *NH*), 3.30 (d, J = 6.2 Hz, 2H, *CH_2*), 3.04 (s, 1H, ≡*CH*), 1.38 (bs, 9H, *tBu*), 1.29 (s, 6H, *CH_3*). ^{13}C NMR shifts extrapolated from the HSQC experiment: δ = 132.1 (*<u>CH</u>-C-C≡ Ar*), 129.9, 129.9, 126.1 (*CH Ar*), 77.3 (≡*CH*), 51.7 (*CH_2*), 28.3 (*tBu*), 26.2 (*CH_3*).

1.3.7. Synthesis of **4-(1-((tert-butoxycarbonyl)amino)-2-methylpropan-2-yl) benzoic acid 33**

Compound **32** (246 mg, 0.90 mmol, 1 eq) was dissolved in tBuOH (30 mL). An aqueous solution of $NaHCO_3$ (250 mg, 3.0 mmol, 3.3 eq in 30 mL of water) and an acqueous solution of $KMnO_4$ (426 mg, 2.70 mmol, 3 eq in 30 mL of water) were added to the dissolved alkyne and stirred at 40 °C for 1 h. The reaction mixture was quenched by stirring with MeOH, then was filtered and concentrated. The crude was redissolved in CH_2Cl_2 and acidified to pH 2, using 1M HCl aqueous solution. The organic phase was washed with brine, dried over Na_2SO_4, filtered and concentrated under reduced pressure. The crude product was purified by automatic chromatography (Biotage Sfär 10: nHex/EtOAc gradient 0 % to 60 %; 0.01% formic acid) affording acid **33** (177 mg, 0.60 mmol, y = 67 %). TLC R_f (nHex/EtOAc: 8/2; 0.01% formic acid): 0.16. MS (ESI) calculated for $C_{16}H_{23}NO_4$ $[M + Na]^+$ *m/z*:

316.15; found: 316.31. ^{1}H NMR (400 MHz, $CDCl_3$): δ = 8.06 (d, 2H, *CH-C-C=O Ar*), 7.46 (m, 2H, *CH Ar*), 4.33 (bs, 1H, *NH*), 3.37 (d, J = 6.1 Hz, 2H, *CH_2*), 3.29 (d, 2H, *CH_2*), 1.39 (bs, 9H, *tBu*), 1.36 (s, 6H, *CH_3*). ^{13}C NMR shifts extrapolated from the HSQC experiment: δ = 131.1 (*CH-C-I Ar*), 127.3 (*CH Ar*), 52.7 (*CH_2*), 29.3 (*tBu*), 27.4 (*CH_3*).

1.3.8. Synthesis of **quinoline-6-carbaldehyde 19**

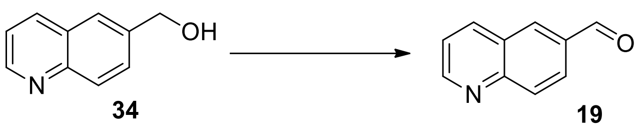

6-quinolinylmethanol **34** (71 mg, 0.44 mmol, 1 eq) was dissolved in dry CH_2Cl_2 (2.4 mL) under nitrogen. Dess-Martin Periodinane (1.8 mL, 0.3 M in CH_2Cl_2) was added (final concentration = 0.1 M) and the reaction mixture was stirred until TLC revealed completion (3 h). The reaction was diluted with CH_2Cl_2 and the organic layer was washed with NaOH 1 M aqueous solution (2x) and brine, that dried over Na_2SO_4 and concentrated under reduced pressure to afford the the crude product **19** (63 mg y = 88 %), which was used without further purification. TLC R_f (nHex/EtOAc: 6/4): 0.24. MS (ESI) calculated for $C_{10}H_7NO$ $[M + H]^+$ *m/z*: 158.06; found: 158.00. ^{1}H NMR (400 MHz, $CDCl_3$): δ = 10.21 (s, 1H, *H-10*), 9.05 (d, 1H, *H-1*), 8.34 (mult., 2H, *H-3* + *H-5*), 8.21 (mult., 2H, *H-7* + *H-8*), 7.52 (m, 1H, *H-2*). In accordance with published data[11]

1.4. Synthesis of amides 6a-e from azide 2

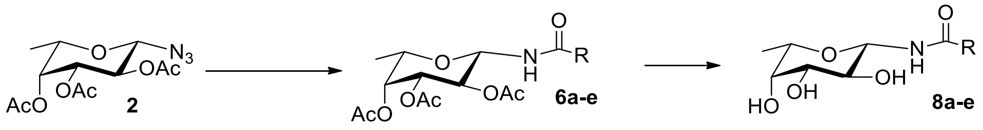

General procedure for **amide bond formation** through Staudinger ligation, adapted from Bianchi and co-workers [12]

To a solution of **(2,3,4-tri-*O*-acetyl β-L-fucopyranosyl) azide 2** (1.1 eq) in dry CH_2Cl_2 (concentration = 0.15 M) 1 M solution of PMe_3 in toluene (1.5 eq) was added under nitrogen atmosphere. The reaction mixture was stirred at room temperature for 0.5-1 h, until TLC showed the full reduction, before being concentrated. On a second flask, the carboxylic acid (**Table S2,** 1.0 eq) was dissolved in dry DMF (concentration = 0.15 M) under nitrogen atmosphere and N,N-Diisopropylethylamine (DIPEA, 2.2 eq) and Hexafluorophosphate Azabenzotriazole Tetramethyl Uronium (HATU, 1.1 eq) were added. This mixture was stirred for 1 h before being added to the reduced fucoside, adding DMF (concentration: 0.10 M). The resulting solution was stirred overnight, then was concentrated, redissolved in CH_2Cl_2, and washed with a 1 M HCl aqueous solution and saturated $NaHCO_3$ aqueous solution. The organic phase was dried over Na_2SO_4, filtered and concentred under reduced pressure. The crude product was purified by automatic chromatography (nHex/EtOAc).

Table S2. Acids used for the synthesis of amides from fucosylazide **2.**

Product	Acid	Product	Acid
**6a**	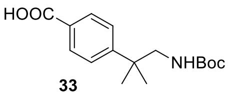 **33**	**6d**	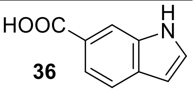 **36**
**6b**	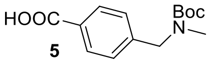 **5**	**6e**	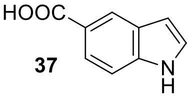 **37**
**6c**	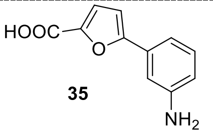 **35**		

1.4.1. Synthesis of ***tert*-butyl (2-(4-((2,3,4-tri-*O*-acetyl β-L-fucopyranosyl)carbamoyl)phenyl)-2-methylpropyl) carbamate 6a**

(2,3,4-tri-*O*-acetyl β-L-fucopyranosyl) azide 2 (0.20 mmol) was coupled to **4-(1-((*tert*-butoxycarbonyl)amino)-2-methylpropan-2-yl)benzoic acid 33** (0.17 mmol) affording **6a** (0.08 mmol, y = 45 %). TLC R_f (nHex/EtOAc: 6/4): 0.10. MS (ESI) calculated for $C_{28}H_{40}N_2O_{10}$ $[M + Na]^+$ *m/z*: 587.26; found: 587.34. ^{1}H NMR (400 MHz, $CDCl_3$): δ = 7.73 (d, J = 8.5 Hz, 2H, *CH Ar*), 7.42 (d, J = 8.4 Hz, 2H, *CH Ar*), 7.03 (d, J_{NH-1} = 9.0 Hz, 1H, *NH*), 5.38 (t, J_{1-NH} = J_{1-2} = 9.0 Hz, 1H, *H-1*), 5.32 (dd, J_{4-3} = 2.9 Hz J_{4-5} = 1.2 Hz, , 1H, *H-4*), 5.27 - 5.18 (mult., 1H, *H-2* + *H-3*), 4.24 (t, J_{NH-CH2} = 6.4 Hz, 1H, *NH-Boc*), 4.01 (dq, J_{5-4} = 1.1 Hz, J_{5-CH3} = 6.4 Hz, 1H, *H-5*), 3.31 (bd, J_{NH-CH2} = 6.4 Hz, 2H, *CH_2*), 2.18, 2.04, 2.01 (s, 3H, *OAc*), 1.39 (bs, 9H, *tBu*), 1.32 (bs, 6H, 2x*CH_3*), 1.26 (d, J_{CH3-5} = 6.4 Hz, 3H, *H-6*). ^{13}C chemical shifts were extrapolated from the HSQC experiment: δ = 127.5, 126.6 (*para CH Ar*), 130.2, 125.3, 125.0, 79.0 (*C1*), 71.1 (*C5*), 70.6 (*C4*), 71.0 - 68.8 (*C3,C2*), 52.0 (*CH_2*), 28.6 (*CH_3 tBu*), 26.5 (2x*CH_3*), 20.9 (*CH_3 OAc*), 16.2 (*C6*).

1.4.2. Synthesis of ***tert*-butyl (4-((2,3,4-tri-*O*-acetyl β-L-fucopyranosyl)carbamoyl)benzyl) (methyl) carbamate 6b**

(2,3,4-tri-*O*-acetyl β-L-fucopyranosyl) azide 2 (0.154 mmol) was coupled to **4-(Boc-aminomethyl)benzoic acid 5** (0.139 mmol) affording **6b** (0.072 mmol, y = 52 %). TLC R_f (nHex/EtOAc: 6/4): 0.10. $[\alpha]_D^{17}$ = 7.23 ($CHCl_3$, c 1). MS (ESI) calculated for $C_{26}H_{36}N_2O_{10}$ [M + Na]+ m/z: 559.23; found: 559.30. ^{1}H NMR (400 MHz, $CDCl_3$): δ = 7.74 (d, J = 8.2 Hz, 2H, *CH Ar*), 7.29 (d, J = 7.6 Hz, 2H, *CH Ar*), 7.04 (d, J_{NH-1} = 8.9 Hz, 1H, *NH*), 5.38 (d, J_{1-2} = 9.0 Hz, 1H, *H-1*), 5.33 (d, J_{4-3} =2.4 Hz, 2H, *H-4*), 5.25 - 5.18 (mult., J_{2-1} = J_{2-3} = 10.3 Hz, 1H, *H-2* + *H-3*), 4.45 (bs, 2H, *CH_2*), 4.02 (q, J_{5-CH3} = 6.5 Hz, 1H, *H-5*), 2.86 - 2.80 (bd, 3H, *N-CH_3*), 2.19, 2.04, 2.02 (s, 3H, *OAc*), 1.49 - 1.44 (bs, 9H, *tBu*), 1.22 (d, J_{CH3-5} = 6.5 Hz, 3H, *CH_3*). ^{13}C NMR (400 MHz, $CDCl_3$): δ = 172.2, 170.6, 170.0 (C=O Ac), 166.9 (NH-C=O), 143.0 (*CH_2-C̲ Ar*), 132.0 (O=*C*-*C̲ Ar*), 127.7 (*CH Ar*), 80.1 (*C tBu*), 79.2 (*C1*), 71.3 (*C3*), 71.0 (*C5*), 70.6 (*C4*), 68.8 (*C2*), 52.7 (*CH_2*), 34.4 (*CH_3*), 28.6 (*CH_3 tBu*), 21.0, 20.8, 20.8 (*CH_3 OAc*), 16.3 (*C6*).

1.4.3.Synthesis of **5-(3-aminophenyl)furan-2-carboxamido-(2,3,4-tri-*O*-acetyl-β-L-fucopyranose) 6c**

(2,3,4-tri-*O*-acetyl β-L-fucopyranosyl) azide 2 (0.082 mmol) was coupled to **5-(3-aminophenyl)furan-2-carboxylic acid 35** (0.064 mmol) affording **6c** (0.015 mmol, y = 23 %). TLC R_f (nHex/EtOAc: 6/4): 0.35. MS (ESI) calculated for $C_{23}H_{26}N_2O_9$ $[M + H]^+$ *m/z*: 475.17; found: 474.75. $[M - H]^-$ *m/z*: 473.16; found: 472.85. ^1H NMR (400 MHz, $CDCl_3$): δ = 7.28 (d, 1H, *NH*), 7.22 (mult., J_{ortho} = 7.9 Hz, 2H, *CH Ar*), 7.15 - 7.12 (mult., 2H, *CH Ar*), 6.69 (mult., J = 3.8 Hz, 2H, *CH hAr*), 5.38 - 5.26 (mult., $J_{2\text{-}1}$ = 10.0 Hz, 3H, *H-1 + H-2 + H-4*), 5.20 (dd, $J_{3\text{-}2}$ = 9.8 Hz, $J_{3\text{-}4}$ = 3.1 Hz, 1H, *H-3*), 4.02 (dq, $J_{5\text{-}CH3}$ = 6.5 Hz, 1H, *H-5*), 2.21, 2.04, 2.02 (s, 3H, *OAc*), 1.23 (d, $J_{CH3\text{-}5}$ = 6.5 Hz, 3H, CH_3). ^{13}C chemical shifts were extrapolated from the HSQC experiment: δ = 129.8 (*C5'*), 117.6 (*C4'*), 115.6 (CH_{furane}), 115.1 (*C6'*), 110.9 (*C2'*), 107.0 (CH_{furane}), 78.3 (*C1*), 71.0 (*C3*), 70.9 (*C5*), 70.4 (*C4*), 68.3 (*C2*), 20.7 (CH_3 *OAc*), 16.0 (*C6*)

1.4.4. Synthesis of ***N*-(2,3,4-tri-*O*-acetyl β-L-fucopyranosyl)-1H-indole-6-carboxamide 6d**

(2,3,4-tri-*O*-acetyl β-L-fucopyranosyl) azide 2 (0.079 mmol) was coupled to **1H-indole-6-carboxylic acid 36** (0.095 mmol) affording **6d** (0.023 mmol, y = 29 %). TLC R_f (nHex/EtOAc: 1/1): 0.27. ^1H NMR (400 MHz, $CDCl_3$): δ = 8.62 (bs, 1H, *NH Ar*), 7.97 (s, 1H, *H-4'*), 7.66 (d, J = 8.4 Hz, 1H, *H-5'*), 7.46 (dd, J = 8.4 Hz, J' = 1.1 Hz, 1H, *H-7'*), 7.36 (t, J'' = 2.7 Hz, 1H, *H-1'*), 7.12 (d, $J_{NH\text{-}1}$ = 9.0 Hz, 1H, *NH-C=O*), 6.59 (m, 1H, *H-2'*), 5.44 (t, $J_{NH\text{-}1}$ = $J_{1\text{-}2}$ = 8.9 Hz, 1H, *H-1*), 5.34 (d, $J_{4\text{-}3}$ = 2.8 Hz, 1H, *H-4*), 5.30 - 5.20 (mult., 2H, *H-2* + *H-3*), 4.04 (dq, $J_{5\text{-}CH3}$ = 6.5 Hz, 1H, *H-5*), 2.19, 2.03, 2.03 (s, 3H, *OAc*), 1.22 (d, $J_{CH3\text{-}5}$ = 6.5 Hz, 3H, CH_3). ^{13}C chemical shifts were extrapolated from the HSQC experiment: δ = 127.0 (*C1'*), 120.6 (*C5'*), 118.2 (*C7'*), 111.3 (*C4'*), 102.7 (*C2'*), 79.0 (*C1*), 71.1 (*C3*), 70.8 (*C5*), 70.5 (*C4*), 68.7 (*C2*), 20.5 (CH_3 *OAc*), 16.2 (*C6*).

1.4.5. Synthesis of ***N*-(2,3,4-tri-*O*-acetyl β-L-fucopyranosyl)-1H-indole-5-carboxamide 6e**

(2,3,4-tri-*O*-acetyl β-L-fucopyranosyl) azide 2 (0.104 mmol) was coupled to **1H-indole-5-carboxylic acid 37** (0.124 mmol) affording **6e** (0.014 mmol, y = 13 %). TLC R_f (nHex/EtOAc: 1/1): 0.16. ^1H NMR (400 MHz, $CDCl_3$): δ = 8.44 (bs, 1H, *NH Ar*), 8.12 (s, 1H, *H-4'*), 7.64 (dd, J = 8.7 Hz, J' = 1.5 Hz, 1H, *H-6'*), 7.41 (d, J = 8.6 Hz, 1H, *H-7'*), 7.28 (t, J'' = 2.8 Hz, 1H, *H-1'*), 7.06 (d, $J_{NH\text{-}1}$ = 9.0 Hz, 1H, *NH-C=O*), 6.65 (m, 1H, *H-2'*), 5.45 (t, $J_{NH\text{-}1}$ = $J_{1\text{-}2}$ = 9.0 Hz, 1H, *H-1*), 5.34 (d, $J_{4\text{-}3}$ = 2.6 Hz, 1H, *H-4*), 5.28 (t, $J_{2\text{-}1}$ = $J_{2\text{-}3}$ = 9.0 Hz, 1H, *H-2*), 5.21 (dd, $J_{3\text{-}2}$ = 10.3 Hz, $J_{3\text{-}4}$ = 3.3 Hz, 1H, *H-3*), 4.03 (dq, $J_{5\text{-}CH3}$ = 6.5 Hz, 1H, *H-5*), 2.20, 2.03, 2.03 (s, 3H, *OAc*), 1.22 (d, $J_{CH3\text{-}5}$ = 6.5 Hz, 3H, CH_3). ^{13}C chemical shifts were extrapolated from the HSQC experiment: δ = 125.3 (*C1'*), 121.2 (*C6'*), 120.8 (*C4'*), 111.0 (*C7'*), 103.8 (*C2'*), 79.1 (*C1*), 71.0 (*C3*), 70.8 (*C5*), 70.4 (*C4*), 68.6 (*C2*), 20.6 (CH_3 *OAc*), 16.1 (*C6*).

1.5. Synthesis of triazoles 10a-b from azide 2

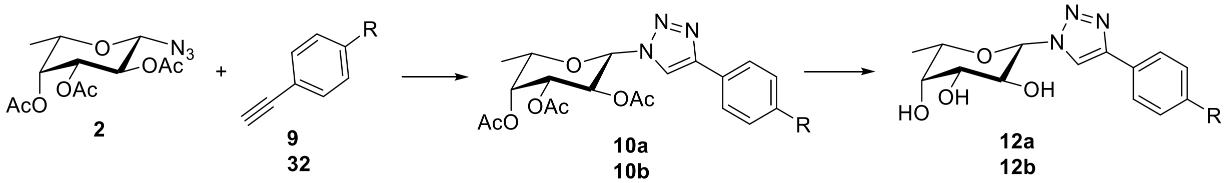

General procedure for **copper-catalysed azide–alkyne cycloaddition (CuAAC)**

Degassed water was used to prepare stock solutions of $CuSO_4 \cdot 5H_2O$ (0.04 M) and sodium ascorbate (0.16 M) under N_2 atmosphere. Alkyne **9** or **32** (1.0 eq, concentration: 0.2 M) was dissolved in degassed THF under N_2 atmosphere and equal parts of the two stock solutions were added to match a THF/H_2O 1:1 ratio ($CuSO_4$: 0.1 eq, sodium ascorbate: 0.4 eq). Lastly, **(2,3,4-tri-*O*-acetyl β-L-fucopyranosyl) azide 2** (1 eq) was added to the mixture and stirred at room temperature overnight. The reaction mixture was concentrated and re-dissolved in H_2O/ CH_2Cl_2 (1:1), then washed with a NH_3/NH_4Cl (1:1) solution to remove copper salts. The organic phase was was dried over Na_2SO_4, filtered and concentrated under reduced pressure. The crude product was used for the following step directly or after purification by flash or automatic chromatography (nHex/EtOAc).

1.5.1. Synthesis of ***tert*-butyl (2-(4-(1-(2,3,4-tri-*O*-acetyl β-L-fucopyranosyl)-1H-1,2,3-triazol-4-yl)phenyl)-2-methylpropyl) carbamate 10a**

(2,3,4-tri-*O*-acetyl β-L-fucopyranosyl) azide 2 (0.16 mmol) was coupled to ***tert*-butyl (2-(4-ethynylphenyl)-2-methylpropyl)carbamate 9** (0.16 mmol) affording **10a** (0.14 mmol, y = 86 %). TLC R_f (nHex/EtOAc: 6/4): 0.22. ^{1}H NMR (400 MHz, $CDCl_3$): δ = 8.03 (s, 1H, *CH hAr*), 7.81 (d, J = 8.4 Hz, 2H, *CH Ar*), 7.41 (d, J = 8.4 Hz, 2H, *CH Ar*), 5.86 (d, J_{1-2} = 9.4 Hz, 1H, *H-1*), 5.60 (dd, J_{2-1} = 9.4 Hz, J_{2-3} = 10.2 Hz, 1H, *H-2*), 5.41 (d, J_{4-3} = 3.4 Hz, J_{4-5} = 1.2 Hz, 1H, *H-4*), 5.26 (dd, J_{3-2} = 10.3 Hz, J_{3-4} = 3.4 Hz, 1H, *H-3*), 4.29 (bt, J = 6.1 Hz, 1H, *NH*), 4.15 (dq, J_{5-4} = 1.2 Hz, J_{5-CH3} = 6.4 Hz, 1H, *H-5*), 3.38 (bd, J = 6.3 Hz, 2H, *CH_2*), 2.26, 2.01, 1.88 (s, 3H, *OAc*), 1.39 (bs, 9H, *tBu*), 1.33 (s, 6H, 2x*CH_3*), 1.28 (d, J_{6-5} = 6.4 Hz, 3H, *H-6*).

1.5.2. Synthesis of ***tert*-butyl (4-(1-(2,3,4-tri-*O*-acetyl β-L-fucopyranosyl)-1H-1,2,3-triazol-4-yl)benzyl) (methyl) carbamate 10b**

(2,3,4-tri-*O*-acetyl β-L-fucopyranosyl) azide 2 (0.14 mmol) was coupled to ***tert*-butyl (4-ethynylbenzyl)(methyl)carbamate 32** (0.14 mmol) affording **10b** (quantitative yield). TLC R_f (nHex/EtOAc: 6/4): 0.27. $[\alpha]_D^{19.6}$ = 51.13 ($CHCl_3$, 1). MS (ESI) calculated for $C_{27}H_{36}N_4O_9$ $[M + Na]^+$ *m/z*: 583.24; found: 583.26. ^{1}H NMR (400 MHz, $CDCl_3$): δ = 8.03 (s, 1H, *CH Ar*), 7.81 (d, J = 8.0 Hz, 2H, *CH Ar*), 7.28 (bs, J = 8.1 Hz, 2H, *CH Ar*), 5.86 (d, J_{1-2} = 9.3 Hz, 1H, *H-1*), 5.59 (t, J_{2-1} = J_{2-3} = 9.7 Hz, 1H, *H-2*), 5.40 (dd, J_{4-3} = 3.2 Hz, 1H, *H-4*), 5.26 (dd, J_{3-2} = 10.2 Hz, J_{3-4} = 3.3 Hz, 1H, *H-3*), 4.43 (bs, 2H, *CH_2*), 3.99 (qd, J_{5-CH3} = 6.4 Hz, 1H, *H-5*), 2.85 - 2.79 (bd, 3H, *N-CH_3*), 2.25, 2.01, 1.88 (s, 3H, *OAc*), 1.47 (bs, 9H, *tBu*), 1.27 (d, J_{CH3-5} = 6.4 Hz, 3H, *CH_3*). ^{13}C NMR (100 MHz, $CDCl_3$): δ = 170.5, 170.0, 169.4 (*C=O*), 148.2 (*C hAr*), 138.6 (*CH_2-<u>C</u> Ar*), 129.2 (*hAr-<u>C</u>* Ar), 128.4 - 127.7 (*CH Ar*), 126.2 (*CH Ar*), 117.8 (*CH hAr*), 86.5 (*C1*), 79.9 (*C tBu*), 72.9 (*C5*), 71.4 (*C3*), 70.0 (*C4*), 68.0 (*C2*), 52.6 (*CH_2*), 34.1 (*CH_3*), 28.6 (*CH_3 tBu*), 20.8, 20.7, 20.4 (*CH_3 OAc*), 16.2 (*C6*).

1.6. Synthesis of alkynes 20a-b by Sonogashira coupling from 3b

1.6.1. Synthesis of ***tert*-butyl (2-(4-(2,3,4-tri-*O*-acetyl β-L-fucopyranosylethynyl)phenyl)-2-methylpropyl)carbamate 20a**

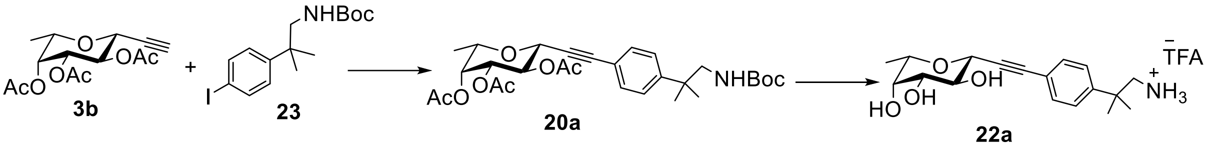

The alkyne **3b** (130 mg, 0.44 mmol, 1 eq) and compound **23** (180 mg, 0.48 mmol, 1.1 eq) were dissolved in DMF ([0.08 M], 5.5 mL) and added to an Argon flushed sealed tube with triphenylphosphine (25.4 mg, 5% mol) and CuI (33.51 mg, 40% mol). TEA (0.080 mL, 0.57 mmol, 1.3 eq) was then added to the mixture and the reaction was stirred at 80 °C until the TLC showed the full consumption of the starting material (3 h). The solvent was removed by using a smart evaporator and the residue was dissolved in ethyl acetate and washed with saturated solution of NH_4Cl, saturated solution of $NaHCO_3$ and brine. The organic layer was dried over Na_2SO_4, filtered and concentred under reduced pressure. The crude was purified by automatic chromatography (Biotage Sfär 10: nHex/EtOAc gradient 0 % to 30 %) affording the pure compound (y = 87%). TLC R_f (nHex/EtOAc: 7/3): 0.27. MS (ESI) calculated for $C_{29}H_{39}NO_9$ $[M + Na]^+$ *m/z*: 568.25; found: 568.24. ^1H NMR (400 MHz, $CDCl_3$): δ = 7.40 (d, *J* = 8.2 Hz, 2H, *CH* Ar), 7.29 (d, *J* = 8.2 Hz, 2H, *CH* Ar), 5.48 (t, *J* = 10.0 Hz, 1H, *H-2*), 5.31 (d, *J* = 3.0 Hz, 1H, *H-4*), 5.07 (dd, *J* = 10.2, *J* = 3.3 Hz, 1H, *H-3*), 4.39 (d, *J* = 9.8 Hz, 1H, *H-1*), 4.28 (bs, 1H, NH), 3.87 (q, *J* = 6.3 Hz, 1H, *H-5*), 3.31 (d, *J* = 6.0 Hz, 2H, CH_2), 2.21 (s, 3H, *OAc*), 2.08 (s, 3H, *OAc*), 2.01 (s, 3H, *OAc*), 1.40 (s, 9H, *tBu*), 1.30 (s, 6H, 2xCH_3), 1.26 (d, *J* = 6.3 Hz, 3H, *H-6*).

1.6.2. Synthesis of ***tert*-butyl (4-(2,3,4-tri-*O*-acetyl β-L-fucopyranosylethynyl)benzyl)(methyl) carbamate 20b,** adapted from Lowary and co-workers [5]

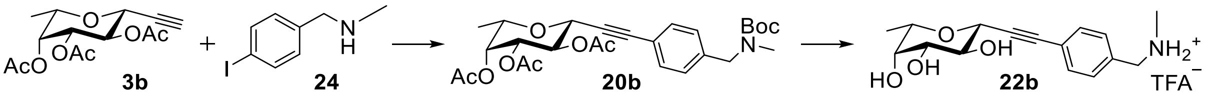

The iodide **24** (0.37 mmol, 1.1 eq) was dissolved in piperidine (concentration: 0.2 M) and added to a flask containing the acetylene **3b** (0.34 mmol , 1 eq), $Pd(PPh_3)_4$ (0.05 eq) and CuI (0.10 eq) under Ar atmosphere. The reaction mixture was heated to 80 °C for 2 h until TLC showed completion, before returning to room temperature. The crude was concentrated and re-dissolved in pyridine (1 - 2 mL) and Ac_2O (1 - 2 mL) and stirred overnight at room temperature to re-acetylate the deprotected positions. The reaction mixture was cooled to 0 °C and treated with ice-cold MeOH while stirring, then diluted with toluene for co-evaporation of pyridine. Pyridine was also removed by diluting the crude with EtOAc and repeatedly washing with a 0.02 M HCl solution. The organic phase was dried over Na_2SO_4 and concentrated. The crude was purified by automatic chromatography (nHex/EtOAc) affording **20b** (y = 85 %). TLC R_f (nHex/EtOAc: 6/4): 0.55. MS (ESI) calculated for $C_{27}H_{35}NO_9$ $[M + Na]^+$ *m/z*: 540.22; found: 540.24. $[\alpha]_D^{17}$ = -14.0 ($CHCl_3$, c 1). ^1H NMR (400 MHz, $CDCl_3$): δ = 7.38 (d, J = 8.3 Hz, 2H, *≡C-C-<u>CH</u> Ar*), 7.14 (d, J = 7.8 Hz, 2H, *CH Ar*), 5.47 (dd, J_{2-1} = J_{2-3} = 10.0 Hz, 1H, *H-2*), 5.30 (dd, J_{4-3} = 3.4 Hz, J_{4-5} = 1.2 Hz, 1H, *H-4*), 5.05 (dd, J_{3-2} = 10.2 Hz, J_{3-4} = 3.4 Hz, 1H, *H-3*), 4.39 (s, 2H, CH_2), 4.37 (d, J_{1-2} = 9.8 Hz, 1H, *H-1*), 3.85 (qd, J_{5-4} = 1.2 Hz, J_{5-CH3} = 6.4 Hz, 1H, *H-5*), 2.80 (bd, 3H, *N-*CH_3), 2.20 (s, 3H, *OAc*), 2.07 (s, 3H, *OAc*), 2.00 (s, 3H, *OAc*), 1.45 (bs, 9H, *tBu*), 1.25 (d, J_{CH3-5} = 6.4 Hz, 3H, CH_3). ^{13}C NMR (400 MHz, $CDCl_3$), some shifts were extrapolated from the HSQC experiment: δ = 170.8, 170.4, 169.6 (*C=O*), 132.3 (*≡C-C-<u>CH</u> Ar*), 127.3 (*CH Ar*), 120.7

(≡*C*-*C̲ Ar*), 86.4 (C1-*C̲*≡), 83.5 (≡*C̲*-*Ar*), 80.0 (*C tBu*), 73.4 (*C5*), 72.1 (*C3*), 70.7 (*C4*), 69.8 (*C1*), 68.8 (*C2*), 52.2 (*CH_2*), 34.2 (*CH_3*), 28.6 (*CH_3 tBu*), 20.9, 20.9, 20.8 (*CH_3 OAc*), 16.6 (*C6*).

1.7. Synthesis of 1-(quinolin-6-yl)-3-(β-L-fucopyranosyl)prop-2-yn-1-ol 21f from 3a

1.7.1. Synthesis of **1-(quinolin-6-yl)-3-(2,3,4-tri-*O*-benzyl β-L-fucopyranosyl)prop-2-yn-1-ol 20f** following the procedure of Dondoni and co-workers [6]

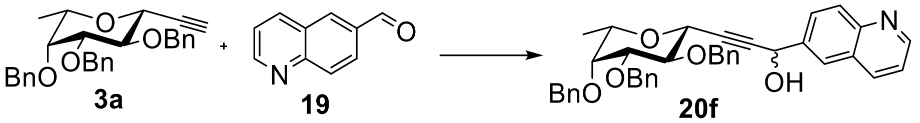

A solution of **(2,3,4-tri-*O*-benzyl β-L-fucopyranosyl) acetylene 3a** (83 mg, 0.19 mmol, 1.2 eq) in dry THF (2.5 mL) was cooled to -20 °C under Ar atmosphere. A freshly made 0.3 M solution of LDA in THF (850 µL, 0.26 mmol, 1.6 eq, prepared from BuLi and DIPA) was added to the solution and left to stir for 10 min while **quinoline-6-carbaldehyde 19** (26 mg, 0.16 mmol, 1 eq) was dissolved in dry THF (1.5 mL), then added to the reaction. The mixture was stirred at -20 °C for 2 h, then at -10 °C for 1h until TLC showed completion. The reaction was quenched with water and extracted with EtOAc. The organic phase was washed with water and dried over Na_2SO_4. The crude product was purified by automatic chromatography (Biotage Sfär 10: nHex/EtOAc gradient from 15% to 100%) affording the diastereomeric mixture **20f** in a 1:1 ratio (72 %), as seen by ^{1}H NMR at 600 MHz (clearly doubled signal for *C̲H̲-OH* and *H-2* at δ = 5.70, 5.69 and 4.02, 3.99 ppm respectively). TLC R_f (CH_2Cl_2/MeOH: 97/3): 0.30. MS (ESI) calculated for $C_{39}H_{37}NO_5$ $[M + Na]^+$ *m/z*: 622.26; found: 622.60. ^{1}H NMR (600 MHz, $CDCl_3$): mixture of **20f** diastereoisomers (ratio 1:1) δ = 8.88 (m, 1H, *H-1'*), 8.06 - 8.00 (mult., $J_{7'-8'}$ = 8.8 Hz, $J_{7'-5'}$ = 5.6 Hz, 2H, *H-3'* + *H-7'*), 7.93 (bs, 1H, *H-5'*), 7.82 (dd, $J_{8'-7'}$ = 8.8 Hz, $J_{8'-5'}$ = 2.0 Hz, 1H, *H-8'*), 7.39 - 7.28 (mult., 11H, *CH Ar* + *H-2'*), 7.19 - 7.12 (mult., 5H, *CH Ar*), 5.70, 5.69 (mult., 1H, *HC-OH*), 5.01 - 4.98 (dd, 1H, *CH_2 Ar*), 4.84 - 4.69 (mult., 5H, *CH_2 Ar*), 4.10 (d, J_{1-2} = 9.6 Hz 1H, *H-1*), 4.02, 3.99 (t, J_{2-1} = J_{2-3} = 9.5 Hz, 1H, *H-2*), 3.63 (m, J_{4-3} = 2.9 Hz, 1H, *H-4*), 3.52 (m, J_{3-2} = 9.4 Hz, J_{3-4} = 2.8 Hz, 1H, *H-3*), 3.48 (m, J_{5-CH3} = 6.5 Hz, 1H, H-5), 3.19 (bs, 1H, *OH*), 1.21 (d, J_{CH3-5} = 6.4 Hz, 3H, *CH_3*). ^{13}C NMR (100 MHz, $CDCl_3$): δ = 149.8 (*C2'*), 148.1, 138.5, 138.45, 138.4, 138.15, 138.1 (*C Ar*) 136.8 (*C4'*), 129.0 (*C7'*), 128.5 (*C8'*), 128.5-127.5 (*CH Ar*), 125.1 (*C5'*), 121.4 (*C3'*), 84.8 (*C≡C*), 84.6 (*C≡C*), 83.8 (*C3*), 78.7 (*C2*), 76.5 (*C4*), 74.8 (*C5*), 75.5, 74.9, 72.9 (*CH_2 OBn*), 70.2 (*C1*), 64.0 (*CH-OH*), 17.2 (*CH_3*).

1.7.2. Synthesis of **1-(quinolin-6-yl)-3-(3,4,5-tris(benzyloxy)-6-methyltetrahydro-2*H*-pyran-2-yl)prop-2-yn-1-yl acetate 21f**

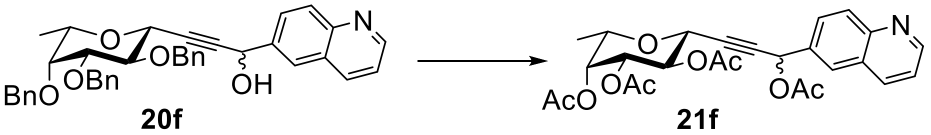

The alcohol **20f** (40 mg, 0.066 mmol, 1 eq) was dissolved in pyridine (0.22 mL, final concentration 0.3 M) and cooled to 0°C (ice bath). The acetic anhydride (11 µL, 0.12 mmol, 1.8 eq) was added to the reaction and the mixture was heated to room temperature for 2 h until TLC revealed completion. The reaction solvents were removed under reduced pressure using methanol as co-evaporating agent to afford the crude acetate diastereomeric mixture (quantitative yield), which was used without purification for the next reaction. TLC Rf (nHex/EtOAc: 5/5): 0.27. MS (ESI) calculated for C41H39NO6 [M + Na]+ m/z: 664.27; found: 664.36 ^{1}H NMR (400 MHz, $CDCl_3$): mixture of diastereoisomers (ratio 45:55) δ = 8.96 (bs, 1H, *H-2'*), 8.14 (mult., 2H, *H-4'* + *H-8'*), 7.97 (d, J = 6.7

Hz, 1H, *H-7'*), 7.86 (d, J = 8.4 Hz, 1H, *H-5'*), 7.45 (dd, J = 4.0 Hz, J = 8.5 Hz, 1H, *H-3'*), 7.43-7.18 (mult., 15H, *CH Ar*), 6.75 (s, 1H *CH-OAc*), 5.04-4.70 (mult., 6H, *CH_2-Ph*), 4.13 (mult., 1H, *H-1*), 4.05, 4.01 (t, $J_{2\text{-}1}$ = $J_{2\text{-}3}$ = 9.5 Hz, 1H, *H-2*), 3.64 (mult., 1H, *H-4*), 3.54 (mult., 1H, *H-3*), 3.50 (q, $J_{5\text{-}CH3}$ = 6.0 Hz, 1H, *H-5*), 2.10, 2.08 (*O=C-CH_3*), 1.22 (d, $J_{CH3\text{-}5}$ = 6.4 Hz, 3H, *CH_3*). ^{13}C NMR (100 MHz, $CDCl_3$): δ = 169.5 (*C=O*), 150.1 (*C2'*), 147.1, 138.4, 138.3, 138.1, 135.5 (*C Ar*), 137.5 (*C4'* or *C8'*), 129.3 (*C4'* or *C8' + C5'*), 128.6-127.4 (*CH Ar*), 127.0 (*C7'*), 121.5 (*C3'*), 85.4 (*C≡C*), 83.8 (*C3*), 81.2 (*C≡C*), 78.6 (*C2*), 76.5 (*C4*), 75.5, 74.8, 72.9 (*CH_2*), 74.9 (*C5*), 70.1 (*C1*), 65.1 (*CH-OAc*), 21.0 (*O=C-CH_3*), 17.2 (*CH_3*).

The crude intermediate (45 mg, 0.07 mmol, 1 eq) was dissolved in Ac_2O (1.40 mL, final concentration = 0.05 M). After 5 min of stirring TMSOTf (110 µL, 0.6 mmol, 9 eq) was added dropwise to the reaction (change of color to brown). The reaction was left stirring for 72 h at room temperature. The mixture was diluted with H_2O and then quenched by adding slowly $NaHCO_3$ saturated aqueous solution. The two phases were separated and the organic layer was washed with $NaHCO_3$ saturated aqueous solution (x3), water (x1) and brine (x1). The organic phase was dried over Na_2SO_4 and concentrated under reduced pressure to afford the crude (45 mg). The crude was purified by automatic chromatography (Biotage Sfär 10 g: CH_2Cl_2/MeOH gradient 0 to 6 % in 10 CV) to afford the diastereoisomers **21f**. (29 mg, y = 69 %). ^{1}H NMR (400 MHz, $CDCl_3$): mixture of **21f** diastereoisomers (ratio 55:45) δ = 8.96 (dd, $J_{2'\text{-}3'}$ = 4.1 Hz, J = 1.4 Hz, 1H, *H-2'*), 8.26 (mult,, 1H, *H-4'*), 8.15 (d, $J_{7'\text{-}8'}$ = 8.5 Hz, 1H, *H-8'*), 7.98 (m, 1H, *H-5'*), 7.82 (dd, *J* = 2.2 Hz, $J_{7'\text{-}8'}$ = 8.5 Hz, 1H, *H-7'*), 7.47 (dd, *J* = 3.8 Hz, J = 8.3 Hz, 1H, *H-3'*), 6.67 (d, 1H *CH-OAc*), 5.45 and 5.43 (t, $J_{2\text{-}1}$ = $J_{2\text{-}3}$ = 9.5 Hz, 1H, *H-2*), 5.30 (d, $J_{3\text{-}4}$ = 3.2 Hz, 1H, *H-4*), 5.03 (mult., 1H, *H-3*), 4.28 (mult., 1H, *H-1*), 3.82 (q, $J_{5\text{-}CH3}$ = 6.0 Hz, 1H, *H-5*), 2.21, 2.16, 2.01, 1.95 (*4xOAc*), 1.24 (d, $J_{CH3\text{-}5}$ = 6.4 Hz, 3H, *CH_3*).^{13}C NMR shifts extrapolated from the HSQC experiment: δ = 151.0 (*C2'*), 136.3 (*C4'*), 130.2 (*C8'*), 128.3 (*C5'*), 126.9 (*C7'*), 122.1 (*C3'*), 73.7 (*C5*), 71.8 (*C3*), 70.4 (*C4*), 68.9 (*C1*), 68.1 (*C2*), 65.0 (*≡C-CH-OAc*), 20.6 (*O=C-CH_3*), 16.1 (*CH_3*).

1.8. Synthesis of 26b by Heck reaction, following the procedure of Kondor and co-workers [13]

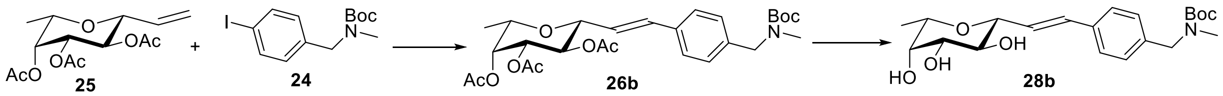

Iodide **24** (32 mg, 0.092 mmol, 1.1 eq) and **(2,3,4-tri-*O*-acetyl β-L-fucopyranosyl) ethene 25** (1 eq) were dissolved in dry DMF (1.7 mL) under Argon atmosphere. $Pd(OAc)_2$ (0.11 eq), KCl (1.1 eq), Bu_4NBr (1.9 eq), K_2CO_3 (1.7 eq) and $AgNO_3$ (0.21 eq) were added to the solution. The reaction mixture was heated to 100 °C for 2.5 h before returning to room temperature. The crude **26b** was purified by automatic chromatography (Biotage Sfär 10: nHex/EtOAc gradient 5 % to 70 %) (35 mg, 0.067 mmol, y = 81 %). TLC R_f (nHex/EtOAc: 7/3): 0.38. MS (ESI) calculated for $C_{27}H_{37}NO_9$ $[M + Na]^+$ m/z: 542.24; found: 542.39. ^{1}H NMR (400 MHz, $CDCl_3$): δ = 7.31 (d, J = 8.0 Hz, 2H, *CH=CH-C-CH Ar*), 7.14 (d, 2H, *CH Ar*), 6.62 (d, J_{trans} = 15.8 Hz, 1H, *C1-CH=CH*), 6.09 (dd, J_{trans} = 15.8 Hz, $J_{CH\text{-}1}$ = 7.8 Hz, 1H, *C1-CH=CH*), 5.31 (d, $J_{4\text{-}3}$ = 3.5 Hz, 1H, *H-4*), 5.22 (dd, $J_{2\text{-}1}$ = 9.8 Hz, $J_{2\text{-}3}$ = 10.0 Hz, 1H, *H-2*), 5.11 (dd, $J_{3\text{-}2}$ = 10.2 Hz, $J_{3\text{-}4}$ = 3.4 Hz, 1H, *H-3*), 4.38 (s, 2H, *CH_2*), 3.97 (dd, $J_{1\text{-}2}$ = $J_{CH\text{-}1}$ = 8.7 Hz, 1H, *H-1*), 3.87 (qd, $J_{5\text{-}4}$ = 0.8 Hz, $J_{5\text{-}CH3}$ = 6.5 Hz, 1H, *H-5*), 2.80 (bd, 3H, *N-CH_3*), 2.19 (s, 3H, *OAc*), 1.99 (s, 3H, *OAc*), 1.93 (s, 3H, *OAc*), 1.47 (bs, 9H, *tBu*), 1.21 (d, $J_{CH3\text{-}5}$ = 6.5 Hz, 3H, *CH_3*). ^{13}C NMR (400 MHz, $CDCl_3$), some shifts were extrapolated from the HSQC experiment: δ = 170.8, 170.4, 169.6 (*C=O*), 138.3 (*CH=CH-C-CH Ar*), 135.2 (*C Ar*), 134.6 (*C1-CH=CH*), 127.6 (*CH=CH-C-CH Ar*), 127.0 (*CH Ar*), 124.8 (*C1-CH=CH*), 80.3 (*C1*), 72.9 (*C5*), 72.3 (*C3*), 71.0 (*C4*), 69.0 (*C2*), 52.5 (*CH_2*), 34.1 (*CH_3*), 28.6 (*CH_3 tBu*), 21.0, 20.9, 20.8 (*CH_3 OAc*), 16.6 (*C6*).

1.9. Removal of protecting groups and synthesis of ligands 8a-e, 12a-b, 28b, 22a-f

Table S3. Panel of β-*N*- and β-*C*-Fucosides.

Fucoside moiety	Fragment moiety	R = Ac R' = Boc	R = H R' = Boc	R = H R' = H
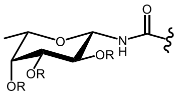	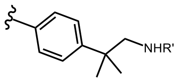	**6a** 45%	**7a** 78%	**8a** quant.
	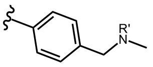	**6b** 52%	**7b** 62%	**8b** quant.
	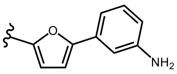	**6c** 23%	-	**8c**[a] 82%
	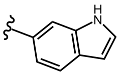	**6d** 29%	-	**8d**[a] 92%
	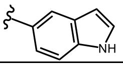	**6e** 13%	-	**8e**[a] 94%
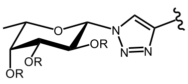	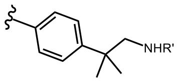	**10a** 86%	**11a** 46%	**12a** quant.
	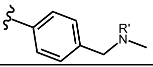	**10b** quant.	**11b** 60%	**12b** quant.
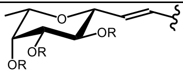	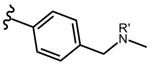	**26b** 81%	**27b** 49%	**28b** quant.
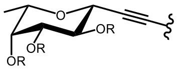	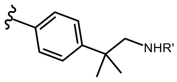	**20a** 94%	**21a** quant.	**22a** quant.
	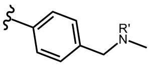	**20b** 85%	**21b** 97%	**22b** quant.
	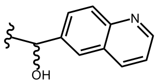	**21f**[b] 72%	-	**22f** 81%[c]
	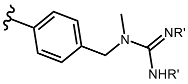	**20g** 43%	**21g** 54%	**22g** quant.

a. Obtained from **6c**-**e**. **b.** R = Bn. **c.** Over two steps (benzyl/acetate exchange and hydrolysis).

1.9.1. General procedure **A** for deacetylation (Zemplén deacetylation)

To a solution of **acetylated compound** (1.0 eq) in dry MeOH (conc.: 0.2 M) a 0.1 M solution of NaOMe in MeOH was added (final conc.: 0.01 M). The reaction mixture was stirred at room temperature for 1 - 3 h until TLC showed completion, before being neutralized with Amberlite® IR_{120} H^+ resin, filtered, and concentrated. The crude was used for the following step directly or after purification by automatic chromatography (CH_2Cl_2/MeOH) or automatic reverse phase chromatography (H_2O/MeOH).

1.9.2. General procedure **B** for deacetylation (deacetylation with NH_2Me,)

To the **acetylated compound** (1.0 eq) dissolved in EtOH (concentration: 0.05 M) was added a 8M solution of NH_2Me in EtOH (final concentration: 4 M). The reaction mixture stirred at room temperature for 2 - 3 h until TLC showed completion, before being concentrated or lyophilized to remove all by-products. The compound was used directly without further purification.

1.9.3 General procedure **C** for Boc-removal

The **Boc-protected compound** (1.0 eq) was dissolved in dry CH_2Cl_2 (concentration: 0.01M) under N_2 atmosphere. The solution was cooled to 0 °C and TFA was added (ratio to CH_2Cl_2 1:9). The reaction

mixture stirred while returning to room temperature for 0.5 - 1 h until TLC showed completion, before being diluted with toluene or MeOH and concentrated. The crude was used directly or after purification by automatic reverse phase chromatography (H_2O/CH_3CN).

1.9.1.1. Synthesis of ***tert*-butyl (2-(4-((β-L-fucopyranosyl)carbamoyl)phenyl)-2-methylpropyl) carbamate 7a**

***tert*-butyl (2-(4-((2,3,4-tri-*O*-acetyl β-L-fucopyranosyl)carbamoyl)phenyl)-2-methylpropyl) carbamate 6a** (0.076 mmol) was deacetylated according to general procedure A affording **7a** (0.059 mmol, y = 78 %). TLC R_f (CH_2Cl_2/MeOH: 95/5): 0.13. MS (ESI) calculated for $C_{22}H_{34}N_2O_7$ $[M + Na]^+$ *m/z*: 461.23; found: 461.28. ^1H NMR (400 MHz, MeOD): δ = 7.86 (d, J = 8.4 Hz, 2H, *CH Ar*), 7.50 (d, J = 8.4 Hz, 2H, *CH Ar*), 5.06 (d, J_{1-2} = 9.0 Hz, 1H, *H-1*), 3.78 (dq, J_{5-4} = 1.1 Hz, J_{5-CH3} = 6.5 Hz, 1H, *H-5*), 3.69 (mult., J_{2-1} = 9.2 Hz, J_{2-3} = 9.4 Hz, J_{4-3} = 3.4 Hz, J_{4-5} = 1.1 Hz, 2H, *H-2* + *H-4*), 3.58 (dd, J_{3-2} = 9.5 Hz, J_{3-4} = 3.3 Hz, 1H, *H-3*), 3.26 (bs, 2H, *CH_2*), 1.38 (bs, 9H, *tBu*), 1.32 (bs, 6H, 2x*CH_3*), 1.26 (d, J_{CH3-5} = 6.5 Hz, 3H, *H-6*). ^{13}C chemical shifts were extrapolated from the HSQC experiment: δ = 127.1, 125.9 (*CH Ar*), 80.5 (*C1*), 74.5 (*C3*), 72.3 (*C5*), 71.5 (*C4*), 69.4 (*C2*), 51.5 (*CH_2*), 27.2 (*CH_3 tBu*), 25. (2x*CH_3*), 15.3 (*C6*).

1.9.1.2. Synthesis of ***Tert*-butyl (4-((β-L-fucopyranosyl)carbamoyl)benzyl) (methyl) carbamate 7b**

***Tert*-butyl (4-((2,3,4-tri-*O*-acetyl β-L-fucopyranosyl)carbamoyl)benzyl) (methyl) carbamate 6b** (0.086 mmol) was deacetylated according to general procedure A affording **7b** (0.054 mmol, y = 62 %). TLC R_f (CH_2Cl_2/MeOH: 95/5): 0.14. MS (ESI) calculated for $C_{20}H_{30}N_2O_7$ $[M + Na]^+$ *m/z*: 433.19; found: 433.32. $[\alpha]_D^{17.1}$ = -7.33 (MeOH, c 1). ^1H NMR (400 MHz, MeOD): δ = 7.89 (d, J = 8.3 Hz, 2H, *CH Ar*), 7.34 (d, J = 8.6 Hz, 2H, *CH Ar*), 5.07 (d, J_{1-2} = 9.0 Hz, 1H, *H-1*), 4.50 (bs, 2H, *CH_2*), 3.78 (qd, J_{5-4} = 1.1 Hz, J_{5-CH3} = 6.5 Hz, 1H, *H-5*), 3.69 (mult., J_{2-1} = J_{2-3} = 9.3 Hz, J_{4-3} = 3.4 Hz, J_{4-5} = 1.1 Hz, 2H, *H-2* + *H-4*), 3.58 (dd, J_{3-2} = 9.6 Hz, J_{3-4} = 3.3 Hz, 1H, *H-3*), 2.86 (bd, 3H, *N-CH_3*), 1.50, 1.48 (bs, 9H, *tBu*), 1.26 (d, J_{CH3-5} = 6.5 Hz, 3H, *CH_3*). ^{13}C chemical shifts were extrapolated from the HSQC experiment: δ = 128.7, 127.9 (*CH Ar*), 81.7 (*C1*), 75.7 (*C3*), 73.5 (*C5*), 72.9 (*C4*), 70.6 (*C2*), 52.6 (*CH_2*), 34.3 (*CH_3*), 28.3 (*CH_3 tBu*), 16.5 (*C6*).

1.9.1.3. Synthesis of ***N*-(β-L-fucopyranosyl)-1*H*-indole-6-carboxamide 8d**

***N*-(2,3,4-tri-*O*-acetyl β-L-fucopyranosyl)-1*H*-indole-6-carboxamide 6d** (0.046 mmol) was deacetylated according to general procedure A affording **8d** (0.042 mmol, y = 92 %). TLC R_f (CH_2Cl_2/Acetone: 1/1): 0.05. $[\alpha]_D^{24.3}$ = -20.0 (MeOH, c 0.5). MS (ESI) calculated for $C_{15}H_{18}N_2O_5$ $[M + Na]^+$ *m/z*: 329.11; found: 329.09. HRMS (ESI^+-TOF) *m/z*: calculated for $C_{15}H_{18}N_2O_5$ $[M + H]^+$: 307.1289, found: 307.1286. ^1H NMR (400 MHz, MeOD): δ = 8.02 (s, 1H, *H-4'*), 7.64-7.56 (mult., 2H, *H-5'*+ *H-7'*), 7.41 (d, $J_{3'-2'}$ = 3.1 Hz, 1H, *H-1'*), 6.51 (dd, $J_{2'-3'}$ = 3.1 Hz, $J_{2'-NH}$ = 0.8 Hz, 1H, *H-2'*), 5.11 (d, J_{1-2} = 9.0 Hz, 1H, *H-1*), 3.79 (m, 1H, *H-5*), 3.76-3.67 (mult., 2H, *H-2* + *H-4*), 3.59 (dd, J_{3-2} = 9.5 Hz, J_{3-4} = 3.3 Hz, 1H, *H-3*), 1.27 (d, J_{CH3-5} = 6.5 Hz, 3H, *CH_3*). ^{13}C NMR (100 MHz, MeOD): δ = 172.2 (*C=O*), 136.9, 132.5, 127.9 (*C hAr*), 128.9 (*C1'*), 120.9, 119.3 (*C5'* + *C7'*), 112.7 (*C4'*), 102.6 (*C2'*), 82.2 (*C1*), 76.0 (*C3*), 73.7 (*C5*), 73.3 (*C4*), 71.0 (*C2*), 17.0 (*C6*).

1.9.1.4. Synthesis of ***tert*-butyl (2-(4-(1-(β-L-fucopyranosyl)-1*H*-1,2,3-triazol-4-yl)phenyl)-2-methylpropyl) carbamate 11a**

***Tert*-butyl (2-(4-(1-(2,3,4-tri-*O*-acetyl β-L-fucopyranosyl)-1*H*-1,2,3-triazol-4-yl)phenyl)-2-methylpropyl) carbamate 10a** (0.157 mmol) was deacetylated according to general procedure A affording **11a** (0.063 mmol, y = 46 %). TLC R_f (CH_2Cl_2/MeOH: 95/5): 0.20. MS (ESI) calculated for

$C_{23}H_{34}N_4O_6$ [M + Na]$^+$ *m/z*: 485.24; found: 485.58. ^{1}H NMR (400 MHz, MeOD): δ = 8.52 (s, 1H, *CH hAr*), 7.78 (d, J = 8.4 Hz, 2H, *CH Ar*), 7.47 (d, J = 8.5 Hz, 2H, *CH Ar*), 5.58 (d, J_{1-2} = 9.2 Hz, 1H, *H-1*), 4.14 (dd, J_{2-1} = 9.3 Hz, J_{2-3} = 9.4 Hz, 1H, *H-2*), 3.99 (dq, J_{5-4} = 1.2 Hz, J_{5-6} = 6.5 Hz, 1H, *H-5*), 3.78 (d, J_{4-3} = 3.3 Hz, 1H, *H-4*), 3.72 (dd, J_{3-2} = 9.5 Hz, J_{3-4} = 3.4 Hz, 1H, *H-3*), 3.26 (bs, 2H, *CH_2*), 1.37 (bs, 9H, *tBu*), 1.32 (mult., 9H, J_{6-5} = 6.4 Hz, 2x*CH_3* + 3x*H-6*). ^{13}C chemical shifts were extrapolated from the HSQC experiment: δ = 129.6, 127.1, 124.3, 124.0 (*meta CH Ar*), 127.4, 126.2 (*para CH Ar*), 120.2 (*meta CH hAr*), 120.0 (*para CH hAr*), 89.9 (*C1*), 75.1 (*C3*), 74.9 (*C5*), 72.6 (*C4*), 70.9 (*C2*), 52.5 (*CH_2*), 28.3 (*CH_3 tBu*), 26.3 (2x*CH_3*), 16.4 (*C6*).

1.9.1.5. Synthesis of ***tert*-butyl (4-(1-(β-L-fucopyranosyl)-1*H*-1,2,3-triazol-4-yl)benzyl) (methyl) carbamate 11b**

***Tert*-butyl (4-(1-(2,3,4-tri-*O*-acetyl β-L-fucopyranosyl)-1*H*-1,2,3-triazol-4-yl)benzyl) (methyl) carbamate 10b** (0.143 mmol) was deacetylated according to general procedure A affording **11b** (0.086 mmol, y = 60 %). TLC R_f (CH_2Cl_2/MeOH: 85/15): 0.48. MS (ESI) calculated for $C_{21}H_{30}N_4O_6$ [M + Na]$^+$ *m/z*: 457.21; found: 457.22. $[\alpha]_D^{17}$ = 6.44 (MeOH, c 1). ^{1}H NMR (400 MHz, MeOD): δ = 8.55 (s, 1H, *CH hAr*), 7.83 (d, J = 8.1 Hz, 2H, *CH Ar*), 7.33 (d, J = 8.1 Hz, 2H, *CH Ar*), 5.57 (d, J_{1-2} = 9.2 Hz, 1H, *H-1*), 4.47 (bs, 2H, *CH_2*), 4.13 (dd, J_{2-1} = J_{2-3} = 9.3 Hz, 1H, *H-2*), 3.99 (qd, J_{5-4} = 1.1 Hz, J_{5-CH3} = 6.5 Hz, 1H, *H-5*), 3.78 (dd, J_{4-3} = 3.4 Hz, J_{4-5} = 1.1 Hz, 1H, *H-4*), 3.72 (dd, J_{3-2} = 9.5 Hz, J_{3-4} = 3.3 Hz, 1H, *H-3*), 2.86 (bd, 3H, *N-CH_3*), 1.48 (bs, 9H, *tBu*), 1.32 (d, J_{CH3-5} = 6.5 Hz, 3H, *CH_3*). ^{13}C NMR (100 MHz, MeOD): δ = 148.7 (*C=O tBu*), 130.8 (*C hAr*), 129.0, 127.0 (*CH Ar*), 120.7 (*CH hAr*), 90.4 (*C1*), 75.5, 75.4 (*C3, C5*), 73.0 (*C4*), 71.3 (*C2*), 52.7 (*CH_2*), 34.5 (*CH_3*), 28.7 (*CH_3 tBu*), 16.8 (*C6*).

1.9.1.6. Synthesis of ***tert*-butyl (2-(4-(β-L-fucopyranosylethynyl)phenyl)-2-methylpropyl)carbamate 21a**

***Tert*-butyl (2-(4-(2,3,4-tri-O-acetyl-β-L-fucopyranosylethynyl)phenyl)-2-methylpropyl) carbamate 20a** (0.051 mmol) was deacetylated according to general procedure A affording **21a** (quantitative yield). TLC R_f (nHex/EtOAc: 1/1 and CH_2Cl_2/MeOH: 9/1): 0.03 and 0.40. ^{1}H NMR (400 MHz, MeOD): δ = 7.43 (d, *J* = 8.4 Hz, 2H, *CH* Ar), 7.38 (d, *J* = 8.4 Hz, 2H, *CH* Ar), 4.09 (d, *J* = 9.6 Hz, 1H, *H-1*), 3.77 – 3.64 (m, 3H, *H-2* + *H-4* + *H-5*), 3.48 (dd, *J* = 9.4, *J* = 3.2 Hz, 1H, *H-3*), 3.23 (s, 2H, *CH_2*), 1.40 (s, 9H, *tBu*), 1.29 – 1.23 (m, 9H, *CH_3* + *H-6*). ^{13}C NMR (100 MHz, MeOD): δ = 158.7 (*C=O tBu*), 149.3 (*$C(CH_3)_2$-<u>C</u> Ar*), 132.7 (*para CH Ar*), 130.8, 130.4, 129.4, 127.3 (*para CH Ar*), 121.7 (*para ≡C-<u>C</u> Ar*), 87.4 (*C1-<u>C</u>≡*), 86.1 (*≡<u>C</u>-Ar*), 80.0 (*C tBu*), 76.1 (*C3* + *C5*), 73.4 (*C4*), 73.0 (*C1*), 72.3 (*C2*), 52.8 (*CH_2*), 40.6 (*<u>C</u>$(CH_3)_2$*), 28.8 (*CH_3 tBu*), 26.7 (2x*CH_3*), 17.2 (*C6*).

1.9.1.7. Synthesis of ***tert*-butyl (4-(β-L-fucopyranosylethynyl)benzyl) (methyl) carbamate 21b**

***Tert*-butyl (4-(2,3,4-tri-*O*-acetyl β-L-fucopyranosylethynyl)benzyl) (methyl) carbamate 20b** (0.097 mmol) was deacetylated according to general procedure A affording **21b** (0.093 mmol, y = 97 %). TLC R_f (nHex/EtOAc: 1/1 and CH_2Cl_2/MeOH: 9/1): 0.03 and 0.39. MS (ESI) calculated for $C_{21}H_{29}NO_6$ [M + Na]$^+$ *m/z*: 414.19; found: 414.25. $[\alpha]_D^{17}$ = 0.5 (MeOH, c 1). ^{1}H NMR (400 MHz, MeOD): δ = 7.45 (d, J = 8.2 Hz, 2H, *CH Ar*), 7.21 (d, J = 8.0 Hz, 2H, *CH Ar*), 4.43 (bs, 2H, *CH_2*), 4.07 (d, J_{1-2} = 9.6 Hz, 1H, *H-1*), 3.69 (mult., 3H, *H-2* + *H-4* + *H-5*), 3.47 (dd, J_{3-2} = 9.5 Hz, J_{3-4} = 3.3 Hz, 1H, *H-3*), 2.83 (bd, 3H, *N-CH_3*), 1.49 (bs, 9H, *tBu*), 1.28 (d, J_{CH3-5} = 6.5 Hz, 3H, *CH_3*). ^{13}C NMR (100 MHz, MeOD): δ = 157.8 (*C=O tBu*), 139.9 (*CH_2-<u>C</u> Ar*), 133.0, 131.7, 129.7 (*meta CH Ar*), 131.4, 128.5 (*para CH Ar*), 124.4 (*meta ≡C-<u>C</u> Ar*), 123.0 (*para ≡C-<u>C</u> Ar*), 87.9 (*meta C1-<u>C</u>≡*), 87.8 (*para C1-<u>C</u>≡*), 85.8 (*≡<u>C</u>-Ar*), 81.4 (*C tBu*), 76.1, 76.0 (*C3* + *C5*), 73.3 (*C2*), 72.8 (*C1*), 72.1 (*C4*), 53.2, 52.4 (*CH_2*), 34.6 (*CH_3*), 28.7 (*CH_3 tBu*), 17.1 (*C6*).

1.9.1.8. Synthesis of **1-(quinolin-6-yl)-3-(β-L-fucopyranosyl)prop-2-yn-1-ol 22f**

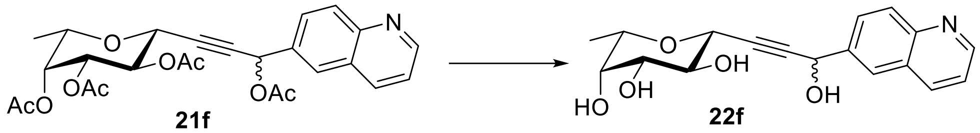

To a solution of **1-(quinolin-6-yl)-3-(2,3,4-tri-*O*-acetyl β-L-fucopyranosyl)prop-2-yn-1-ol 21f** (24 mg, 0.05 mmol) was deacetylated according to the general procedure A affording **22f** (15 mg, 0.046 mmol, y = 81 %). TLC R_f (CH_2Cl_2/MeOH: 9/1): 0.17. MS (ESI) calculated for $C_{18}H_{19}NO_5$ $[M + H]^+$ m/z: 330.13; found: 330.40. EI-HRMS calculated for $C_{18}H_{19}NO_5$ $[M]^{\cdot+}$ m/z: 329.1263; found 329.4100. ^{1}H NMR (400 MHz, $CDCl_3$): mixture of **22f** diastereoisomers (ratio 45:55) δ = 9.12 (d, $J_{2'-3'}$ = 4.1 Hz, 1H, *H-2'*), 9.01 (d, $J_{3'-4'}$ = 8.0 Hz, 1H, *H-4'*), 8.42 (s, 1H, *H-5'*), 8.29 (d, $J_{7'-8'}$ = 9.3 Hz, 1H, *H-8'*), 8.20 (d, $J_{7'-8'}$ = 9.3 Hz, 1H, *H-7'*), 7.97 (dd, $J_{2'-3'}$ = 4.1 Hz, $J_{3'-4'}$ = 8.3 Hz, 1H, *H-3'*), 5.78 (s, 1H *<u>CH</u>-OH*), 3.99 (d, J_{1-2} = 9.5 Hz, 1H, *H-1*), 3.66 (mult., 3H, *H-2* + *H-4* + *H-5*), 3.45 (dd, J_{2-3} = 9.0 Hz, J_{3-4} = 3.0 Hz, 1H, *H-3*), 1.26 (d, J_{CH3-5} = 6.4 Hz, 3H, *CH_3*). ^{13}C NMR (100Mhz, CD_3OD): δ = 150.0 (*C2'*), 147.0, 140.0, 128.2 (*C Ar*), 137.3 (*C4'*), 129.0 (*C7'*), 127.9 (*C8'*), 125.3 (*C5'*), 121.4 (*C3'*), 84.7 (*C≡C*), 83.6 (*C≡C*), 74.6 (*C4* or *C5*), 74.5 (*C3*), 71.9 (*C4* or *C5*), 71.0 (*C1*), 70.75, 70.72 (2x*C2)* 63.2 (*≡C-<u>CH</u>-OH*), 15.6 (*CH_3*).

1.9.1.9. Synthesis of ***tert*-butyl (*E*)-(4-(β-L-fucopyranosylvinyl)benzyl) (methyl) carbamate 27b**

***Tert*-butyl (*E*)-(4-(2,3,4-tri-*O*-acetyl β-L-fucopyranosylvinyl)benzyl) (methyl) carbamate 26b** (0.067 mmol) was deacetylated according to general procedure A affording **27b** (0.033 mmol, y = 49 %). TLC R_f (CH_2Cl_2/MeOH: 95/5): 0.18. MS (ESI) calculated for $C_{21}H_{31}NO_6$ $[M + Na]^+$ *m/z*: 416.20; found: 416.40. ^{1}H NMR (400 MHz, MeOD): δ = 7.41 (d, J = 8.0 Hz, 2H, *Ar*), 7.18 (d, J = 8.1 Hz, 2H, *Ar*), 6.70 (d, J_{trans} = 16.0 Hz, 1H, *=<u>CH</u>-Ar*), 6.32 (dd, J_{trans} = 16.0 Hz, J_{CH-1} = 6.4 Hz, 1H, *C1-<u>CH</u>=*), 4.41 (bs, 2H, *CH_2*), 3.71 (mult., J_{5-4} = 1.3, Hz, J_{5-CH3} = 6.4 Hz, 3H, *H-1* + *H-3* + *H-5*), 3.50 (mult, J_{2-1}= 9.6 Hz, 2H, *H-2* + *H-4*), 2.82 (bd, 3H, *N-CH_3*), 1.47 (bs, 9H, *tBu*), 1.28 (d, J_{CH3-5} = 6.5 Hz, 3H, *CH_3*). ^{13}C NMR (100 MHz, MeOD): δ = 138.8 - 138.5, 137.6, (*C Ar*), 133.3 (*<u>CH</u>-Ar*), 128.7 - 128.6 (*CH Ar*), 128.4 (*C1-<u>CH</u>=*), 127.8 (*CH Ar*), 82.1 (*C5*), 81.3 (*C tBu*), 76.4 (*C2*), 75.5 (*C3*), 73.6 (*C1*), 72.3 (*C4*), 53.3, 52.4 (*CH_2*), 34.4 (*CH_3*), 28.7 (*CH_3 tBu*), 17.2 (*C6*).

1.9.2.1. Synthesis of **5-(3-aminophenyl)furan-2-carboxamido-(β-L-fucopyranose) 8c**

5-(3-aminophenyl)furan-2-carboxamido-(2,3,4-tri-*O*-acetyl-β-L-fucopyranose) 6c (0.017 mmol) was deacetylated according to general procedure B affording **8c** (0.014 mmol, y = 82 %). TLC R_f (nHex/EtOAc: 2/8): 0.05. MS (ESI) calculated for $C_{17}H_{20}N_2O_6$ $[M + Na]^+$ *m/z*: 371.12; found: 371.15. HRMS (ESI$^+$-TOF) *m/z*: calculated for $C_{17}H_{20}N_2O_6$ $[M + Na]^+$: 371.1219, found: 371.1212. ^{1}H NMR (400 MHz, DMSO-d_6): δ = 8.68 (d, J_{NH-1} = 9.1 Hz, 1H, *NH*), 7.28 (d, J = 3.6 Hz, 1H, *CH hAr*), 7.11 - 7.05 (mult., 3H, *H-2'* + *H-5'* + *H-6'*), 6.88 (d, J = 3.6 Hz, 1H, *CH hAr*), 6.56 (dt, J_{ortho} = 7.3 Hz, J_{meta} = 2.0 Hz, 1H, *H-4'*), 5.20 (s, 1H, *NH_2*), 4.88 (t, J_{NH-1} = J_{1-2}= 9.1 Hz, 1H, *H-1*), 4.75 (mult., 2H, *OH-2* + *OH-3*), 4.48 (d, J = 4.0 Hz, 1H, *OH-4*), 3.67 - 3.56 (mult., 2H, *H-2* + *H-5*), 3.48 (t, J_{3-4}= 3.5 Hz, 1H, *H-4*), 3.39 (m, 1H, *H-3*), 1.11 (d, J_{CH3-5} = 6.5 Hz, 3H, *CH_3*). ^{13}C NMR (100 MHz, DMSO-d_6): δ = 157.9 (*C=O*), 155.8 (*C_{furane}-Ar*), 149.0 (*C-NH_2*), 146.3 (*C_{furane}-C=O*), 129.9 (*<u>C</u>-C_{furane}*), 129.3 (*C5'*), 116.2 (*CH_{furane}*), 114.4 (*C4'*), 112.4, 109.3 (*C2'* + *C6'*), 106.7 (*CH_{furane}*), 79.9 (*C1*), 74.3 (*C3*), 71.6 (*C5*), 71.2 (*C4*), 69.0 (*C2*), 16.8 (*C6*).

1.9.2.2. Synthesis of ***N*-(β-L-fucopyranosyl)-1*H*-indole-5-carboxamide 8e**

***N*-(2,3,4-tri-*O*-acetyl β-L-fucopyranosyl)-1*H*-indole-5-carboxamide 6e** (0.014 mmol) was deacetylated according to general procedure B affording **8e** (0.013 mmol, y = 94 %). TLC R_f (CH_2Cl_2/Acetone: 1/1): 0.05. HRMS (ESI$^+$-TOF) *m/z*: calculated for $C_{15}H_{18}N_2O_5$ [M + H]$^+$: 307.1289, found: 307.1285. ^{1}H NMR (400 MHz, MeOD): δ = 8.22 (d, J = 1.8 Hz, 1H, *H-4'*), 7.70 (dd, J' = 8.6 Hz, J = 1.8 Hz, 1H, *H-6'*), 7.44 (dd, J' = 8.6 Hz, J'' = 1.0 Hz, 1H, *H-7'*), 7.33 (d, J''' = 3.2 Hz, 1H, *H-1'*), 6.57 (dd, J''' = 3.2 Hz, J'' = 1.0 Hz, 1H, *H-2'*), 5.10 (d, J_{1-2} = 9.1 Hz, 1H, *H-1*), 3.80 (dq, J_{5-4} = 1.0 Hz, J_{5-CH3} = 6.5 Hz, 1H, *H-5*), 3.77-3.69 (mult., J_{2-3} = 9.3 Hz, J_{4-3} = 3.4 Hz, J_{4-5} = 1.0 Hz, 2H, *H-2 + H-4*), 3.59 (dd, J_{3-2} = 9.5 Hz, J_{3-4} = 3.4 Hz, 1H, *H-3*), 1.27 (d, J_{CH3-5} = 6.5 Hz, 3H, *CH_3*). ^{13}C chemical shifts were extrapolated from the HSQC experiment: δ = 125.5 (*C1'*), 120.5 (*C4' + C6'*), 110.5 (*C7'*), 102.0 (*C2'*), 80.7 (*C1*), 74.6 (*C3*), 72.2 (*C5*), 71.5 (*C4*), 69.6 (*C2*), 15.5 (*C6*).

1.9.3.1. Synthesis of **4-(1-amino-2-methylpropan-2-yl)-*N*-(β-L-fucopyranosyl) benzamide 8a**

***Tert*-butyl (2-(4-((β-L-fucopyranosyl)carbamoyl)phenyl)-2-methylpropyl) carbamate 7a** (0.059 mmol) was deprotected according to general procedure C affording **8a** as a TFA salt (quantitative yield). TLC R_f (CH_2Cl_2/MeOH: 85/15): 0.12. EI-HRMS calculated for $C_{17}H_{26}N_2O_5$ [M]$^{\cdot+}$: 338.1842, found 338.1827. ^{1}H NMR (400 MHz, MeOD): δ = 7.94 (d, J = 8.7 Hz, 2H, *CH Ar*), 7.57 (d, J = 8.7 Hz, 2H, *CH Ar*), 5.07 (d, J_{1-2} = 9.1 Hz, 1H, *H-1*), 3.78 (dq, J_{5-4} = 1.1 Hz, J_{5-CH3} = 6.5 Hz, 1H, *H-5*), 3.70 (mult., J_{2-1} = 9.1 Hz, J_{2-3} = 9.4 Hz, J_{4-3} = 3.3 Hz, J_{4-5} = 1.1 Hz, 2H, *H-2 + H-4*), 3.58 (dd, J_{3-2} = 9.5 Hz, J_{3-4} = 3.3 Hz, 1H, *H-3*), 3.24 (bs, 2H, *CH_2*), 1.47 (bs, 6H, 2x*CH_3*), 1.27 (d, J_{CH3-5} = 6.5 Hz, 3H, *H-6*). ^{13}C NMR (100 MHz, MeOD): δ = 170.4 (*C=O*), 149.6 (*$C(CH_3)_2$-<u>C</u> Ar*), 134.1 (*C Ar*), 129.3 (*CH Ar*), 127.3 (*CH Ar*), 82.0 (*C1*), 76.0 (*C3*), 73.8 (*C5*), 73.2 (*C4*), 70.9 (*C2*), 51.4 (*CH_2*), 38.6 (*<u>C</u>$(CH_3)_2$*), 26.7 (2x*CH_3*), 16.9 (*C6*).

1.9.3.2. Synthesis of ***N*-(β-L-fucopyranosyl)-4-((methylamino)methyl) benzamide 8b**

***Tert*-butyl (4-((β-L-fucopyranosyl)carbamoyl)benzyl) (methyl) carbamate 7b** (0.054 mmol) was deprotected according to general procedure C affording **8b** as a TFA salt (quantitative yield). TLC R_f (CH_2Cl_2/MeOH: 85/15): 0.13. $[\alpha]_D^{17}$ = -5.1 (MeOH, c 1). EI-HRMS calculated for $C_{15}H_{22}N_2O_5$ [M]$^{\cdot+}$: 310.1529, found: 310.1514. ^{1}H NMR (400 MHz, MeOD): δ = 7.99 (d, J = 8.5 Hz, 2H, *CH Ar*), 7.59 (d, J = 8.5 Hz, 2H, *CH Ar*), 5.08 (d, J_{1-2} = 9.1 Hz, 1H, *H-1*), 4.26 (bs, 2H, *CH_2*), 3.79 (qd, J_{5-4} = 1.1 Hz, J_{5-CH3} = 6.5 Hz, 1H, *H-5*), 3.69 (mult., J_{2-1} = J_{2-3} = 9.3 Hz, J_{4-3} = 3.4 Hz, J_{4-5} = 1.1 Hz, 2H, *H-2 + H-4*), 3.58 (dd, J_{3-2} = 9.5 Hz, J_{3-4} = 3.4 Hz, 1H, *H-3*), 2.75 (bd, 3H, *N-CH_3*), 1.27 (d, J_{CH3-5} = 6.5 Hz, 3H, *CH_3*). ^{13}C NMR (100 MHz, MeOD): δ = 170.0 (*C=O*), 136.6, 133.3 (*C Ar*), 130.9, 129.6 (*CH Ar*), 82.0 (*H1*), 76.0 (*C3*), 73.9 (*C5*), 73.2 (*C4*), 70.9 (*C2*), 53.0 (*CH_2*), 34.3 (*CH_3*), 16.5 (*C6*).

1.9.3.3 Synthesis of **2-(4-(1-(β-L-fucopyranosyl)-1*H*-1,2,3-triazol-4-yl)phenyl)-2-methylpropan-1-amine 12a**

***Tert*-butyl (2-(4-(1-(β-L-fucopyranosyl)-1*H*-1,2,3-triazol-4-yl)phenyl)-2-methylpropyl) carbamate 11a** (0.063 mmol) was deprotected according to general procedure C affording **12a** as a TFA salt (quantitative yield). TLC R_f (CH_2Cl_2/MeOH: 85/15): 0.25. EI-HRMS calculated for $C_{18}H_{26}N_4O_4$ [M]$^{\cdot+}$: 362.1954, found 362.1948. ^{1}H NMR (400 MHz, MeOD): δ = 8.57 (s, 1H, *CH hAr*), 7.87 (d, *J* = 8.4 Hz, 2H, *CH Ar*), 7.55 (d, *J* = 8.5 Hz, 2H, *CH Ar*), 5.59 (d, J_{1-2} = 9.2 Hz, 1H, *H-1*), 4.15 (t, J_{2-1} = J_{2-3} = 9.3 Hz, 1H, *H-2*), 3.99 (dq, J_{5-4} = 1.0 Hz, J_{5-6} = 6.5 Hz, 1H, *H-5*), 3.79 (d, J_{4-3} = 3.2 Hz, J_{4-5} = 1.0 Hz, 1H, *H-4*), 3.73 (dd, J_{3-2} = 9.5 Hz, J_{3-4} = 3.3 Hz, 1H, *H-3*), 3.23 (bs, 2H, *CH_2*), 1.47 (bs, 6H, *2xCH_3*), 1.32 (d, J_{6-5} = 6.5 Hz, 6H, *H-6*). ^{13}C NMR (100 MHz, MeOD): δ = 148.4 (*C hAr*),

145.6 (*C(CH₃)₂-C Ar*), 130.5 (*hAr-C Ar*), 127.8 (*CH Ar*), 127.3 (*CH Ar*), 120.9 (*CH hAr*), 90.3 (*C1*), 75.4, 75.4 (*C3* + *C5*), 73.0 (*C4*), 71.3 (*C2*), 51.5 (*CH_2*), 38.4 (*C(CH₃)₂*), 26.8 (2x*CH_3*), 16.8 (*C6*).

1.9.3.4. Synthesis of **1-(4-(1-(β-L-fucopyranosyl)-1*H*-1,2,3-triazol-4-yl)phenyl)-*N*-methylmethanamine 12b**

***Tert*-butyl (4-(1-(2,3,4-tri-*O*-acetyl β-L-fucopyranosyl)-1*H*-1,2,3-triazol-4-yl)benzyl) (methyl) carbamate 11b** (0.143 mmol) was deprotected according to general procedure C affording **12b** as a TFA salt (quantitative yield). TLC R_f (CH_2Cl_2/MeOH: 75/25): 0.35. $[\alpha]_D^{17.6}$ = 5.5 (MeOH, c 1). EI-HRMS calculated for $C_{16}H_{22}N_4O_4$ $[M]^{\cdot+}$: 334.1641, found: 334.1643. ^{1}H NMR (400 MHz, MeOD): δ = 8.63 (s, 1H, *CH hAr*), 7.93 (d, *J* = 8.4 Hz, 2H, *CH Ar*), 7.56 (d, *J* = 8.4 Hz, 2H, *CH Ar*), 5.60 (d, $J_{1\text{-}2}$ = 9.2 Hz, 1H, *H-1*), 4.22 (bs, 2H, *CH_2*), 4.15 (t, $J_{2\text{-}1}$ = $J_{2\text{-}3}$ = 9.3 Hz, 1H, *H-2*), 4.00 (qd, $J_{5\text{-}4}$ = 1.1 Hz, $J_{5\text{-}CH3}$ = 6.5 Hz, 1H, *H-5*), 3.80 (dd, $J_{4\text{-}3}$ = 3.4 Hz, $J_{4\text{-}5}$ = 1.1 Hz, 1H, *H-4*), 3.74 (dd, $J_{3\text{-}2}$ = 9.5 Hz, $J_{3\text{-}4}$ = 3.3 Hz, 1H, *H-3*), 2.75 (bs, 3H, *N-CH_3*), 1.32 (d, $J_{CH3\text{-}5}$ = 6.5 Hz, 3H, *CH_3*). ^{13}C NMR (100 MHz, MeOD): δ = 148.0 (*C hAr*), 133.0, 132.5 (C Ar), 131.6, 127.4 (*CH Ar*), 121.4 (*CH hAr*), 90.3 (*H1*), 75.4, 75.4 (*C3, C5*), 73.0 (*C4*), 71.3 (*C2*), 53.2 (*CH_2*), 33.1 (*CH_3*), 16.8 (*C6*).

1.9.3.5. Synthesis of **(2-(4-(β-L-fucopyranosylethynyl)phenyl)-2-methylpropan-1-amine 22a**

***Tert*-butyl (2-(4-(β-L-fucopyranosylethynyl)phenyl)-2-methylpropyl)carbamate 21a** (0.068 mmol) was deprotected according to general procedure C affording **22a** as a TFA salt (quantitative yield). TLC R_f (CH_2Cl_2/MeOH: 85/15): 0.30. MS (ESI) calculated for $C_{18}H_{25}NO_4$ $[M + H]^+$ *m/z*: 320.19; found: 320.12. HRMS (ESI^+-TOF) *m/z*: calculated for $C_{18}H_{25}NO_4$ $[M + H]^+$: 320.1856, found: 320.1852. ^{1}H NMR (400 MHz, MeOD): δ = ^{1}H NMR (400 MHz, MeOD) δ 7.40 (d, *J* = 8.2 Hz, 2H, *CH Ar*), 7.33 (d, *J* = 8.3 Hz, 2H, *CH Ar*), 3.98 (d, *J* = 9.6 Hz, 1H, *H-1*), 3.60 – 3.56 (m, 3H, , *H-2* + *H-4* + *H-5*), 3.37 (dd, *J* = 9.4, *J* = 3.0 Hz, 1H, *H-3*), 3.09 (s, 2H, *CH_2*), 1.33 (s, 6H, *2xCH_3*), 1.18 (d, *J* = 6.4 Hz, 3H, *H-6*). ^{13}C NMR (100 MHz, MeOD): δ = δ = 146.1 (*C(CH₃)₂-C Ar*), 133.3 (*CH Ar*), 131.4, 130.6, 130.1, 127.3 (*CH Ar*), 122.9 (*≡C-C Ar*), 88.1 (*C1-C≡*), 85.5 (*≡C-Ar*), 76.1, 76.0 (*C3* + *C5*), 73.3 (*C4*), 72.8 (*C1*), 72.2 (*C2*), 51.4 (*CH_2*), 38.5 (*C(CH₃)₂*), 26.7 (2x*CH_3*), 17.1 (*C6*).

1.9.3.6. Synthesis of **1-(4-(β-L-fucopyranosylethynyl)phenyl)-*N*-methylmethanamine 22b**

***Tert*-butyl (4-(β-L-fucopyranosylethynyl)benzyl) (methyl) carbamate 21b** (0.092 mmol) was deprotected according to general procedure C affording **22b** as a TFA salt (quantitative yield). The version of **22b** that was a regioisomeric mixture was separated by HPLC: Gradient from 5 to 80 % of (CH_3CN/H_2O: 9/1; 0.1% TFA) in (H_2O; 0.1% TFA), with peaks coming out at 15 %. The *de novo* synthesis of the fragment circumvented the need for this separation during re-synthesis. TLC R_f (CH_2Cl_2/MeOH: 85/15): 0.20. $[\alpha]_D^{17}$ = 0.4 (MeOH, c 1). MS (ESI) calculated for $C_{16}H_{21}NO_4$ $[M + H]^+$ *m/z*: 292.15; found: 292.09. HRMS (ESI^+-TOF) *m/z*: calculated for $C_{16}H_{21}NO_4$ $[M + H]^+$: 292.1543, found: 292.1543. ^{1}H NMR (400 MHz, MeOD): δ = 7.56 (d, J = 8.3 Hz, 2H, *CH Ar*), 7.45 (d, J = 8.3 Hz, 2H, *CH Ar*), 4.18 (bs, 2H, *CH_2*), 4.09 (d, $J_{1\text{-}2}$ = 9.7 Hz, 1H, *H-1*), 3.69 (mult., $J_{2\text{-}1}$ = $J_{2\text{-}3}$ = 9.7 Hz, 3H, *H-2* + *H-4* + *H-5*), 3.47 (dd, $J_{3\text{-}2}$ = 9.5 Hz, $J_{3\text{-}4}$ = 3.3 Hz, 1H, *H-3*), 2.72 (bs, 3H, *N-CH_3*), 1.28 (d, $J_{CH3\text{-}5}$ = 6.5 Hz, 3H, *CH_3*). ^{13}C NMR (100 MHz, MeOD): δ = 133.5, 130.9 (*CH Ar*), 132.7 (*CH₂-C Ar*), 125.6 (*≡C-C Ar*), 89.4 (*C1-C≡*), 84.9 (*≡C-Ar*), 76.1, 76.0 (*C3* + *C5*), 73.3 (*C2*), 72.8 (*C1*), 72.1 (*C4*), 53.2 (*CH_2*), 33.2 (*CH_3*), 17.1 (*C6*).

1.9.3.7. Synthesis of **1-(4-(β-L-fucopyranosylvinyl)phenyl)-*N*-methylmethanamine 28b**

***Tert*-butyl (*E*)-(4-(β-L-fucopyranosylvinyl)benzyl) (methyl) carbamate 27b** (0.033 mmol) was deprotected according to general procedure C affording **28b** as a TFA salt (quantitative yield). TLC R_f

(CH_2Cl_2/MeOH: 85/15): 0.14. MS (ESI) calculated for $C_{16}H_{23}NO_4$ $[M + H]^+$ m/z: 294.17; found: 294.12. $[\alpha]_D^{16.1}$ = 7.3 (MeOH, c 1). HRMS (ESI$^+$-TOF) calculated for $C_{16}H_{23}NO_4$ $[M + H]^+$ m/z: 294.1705, found: 294.1700. ^{1}H NMR (400 MHz, MeOD): δ = 7.53 (d, J = 8.2 Hz, 2H, *CH Ar*), 7.42 (d, J = 8.2 Hz, 2H, *CH Ar*), 6.74 (d, J_{trans} = 16.0 Hz, 1H, *=CH-Ar*), 6.42 (dd, J_{trans} = 16.0 Hz, $J_{CH\text{-}1}$ = 6.2 Hz, 1H, *C1-CH=*), 4.16 (bs, 2H, *CH₂*), 3.77 - 3.67 (mult., $J_{5\text{-}4}$ = 1.0 Hz, $J_{5\text{-}CH3}$ = 6.5 Hz, 3H, *H-1 + H-3 + H-5*), 3.54 - 3.47 (mult., $J_{2\text{-}1}$ = 9.4 Hz, 2H, *H-2 + H-4*), 2.71 (bs, 3H, *N-CH₃*), 1.29 (d, $J_{CH3\text{-}5}$ = 6.5 Hz, 3H, *CH₃*). ^{13}C NMR (100 MHz, MeOD): δ = 140.0 (*C Ar*), 132.4 (*=CH-Ar*), 131.5 (*C Ar*), 131.1 (*CH Ar*), 130.1 (*C1-CH=*), 128.3 (*CH Ar*), 81.7 (*C5*), 76.4 (*C2*), 75.6 (*C3*), 73.6 (*C1*), 72.3 (*C4*), 53.3 (*CH₂*), 33.0 (*CH₃*), 17.2 (*C6*).

1.10. Syntesis of the guanidine derivative 22g

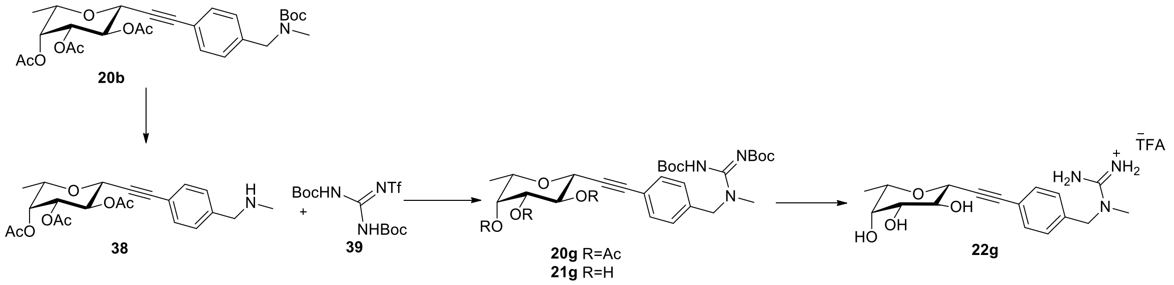

1.10.1. Synthesis of **1-(4-(2,3,4-tri-*O*-acetyl β-L-fucopyranosylethynyl)phenyl)-*N*-methylmethanamine 38**

***Tert*-butyl (4-(2,3,4-tri-*O*-acetyl β-L-fucopyranosylethynyl)benzyl)(methyl)carbamate 20b** was deprotected according to general procedure C affording **38** as a TFA salt (y = 85%). TLC Rf (CH_2Cl_2/MeOH: 95/5): 0.36. MS (ESI) calculated for $C_{22}H_{27}NO_7$ $[M + H]^+$ m/z: 418.19; found: 418.41; [M + Na]+ m/z: 440.17; found: 440.43.^{1}H NMR (400 MHz, $CDCl_3$) δ = 7.40 (d, *J* = 8.0 Hz, 2H, *CH Ar*), 7.27 (d, *J* = 7.6 Hz, 2H, *CH Ar*), 5.49 (dd, *J* = 10.0 Hz, *J* = 3.5 Hz, 1H, *H-2*), 5.31 (s, 2H, *CH₂*), 5.07 (dd, *J* = 10.2, *J* = 3.4 Hz, 1H, *H-3*), 4.39 (d, *J* = 9.8 Hz, 1H, *H-1*), 3.87 (q, *J* = 6.5 Hz, 1H, *H-5*), 2.21 (s, 3H, *OAc*), 2.08 (s, 3H, *OAc*), 2.02 (s, 3H, *OAc*), 1.25 (d, *J* = 5.6 Hz, 3H, *CH₃*). ^{13}C NMR (100 MHz, $CDCl_3$): δ = 170.8, 170.4, 169.6 (C=O), 132.3 (CH Ar), 128.3 (CH Ar), 120.7 (≡C-C Ar), 86.4 (C1-C≡), 83.5 (≡C-Ar), 73.3 (C5), 71.9 (C3), 70.5 (C4), 69.8 (C1), 68.8 (C2), 54.9 (CH_2), 35.0 (CH_3), 20.9, 20.9, 20.8 (CH_3 OAc), 16.6 (C6).

1.10.2. Synthesis of ***N', N''*-bis(*tert*-butoxycarbonyl) 1-(4-(2,3,4-tri-*O*-acetyl β-L-fucopyranosylethynyl)benzyl)-*N*-methylguanidine 20g,** following the procedure of Goodman and co-workers[14]

The Goodman's reagent **39** (47 mg, 0.120 mmol, 1.6 eq) was dissolved in dry CH_2Cl_2 (150 µL) under N_2 atmosphere. TEA (52 µL, 0.370 mmol, 5 eq) was added to the reaction and the whole was left stirring for 5 min. In the meantime, the amine **38** (31 mg, 0.074 mmol, 1 eq) was dissolved in dry CH_2Cl_2 (150 µL) and added to the mixture (final c = 0.25 M) and the whole was left stirring at 42 °C for 6 d. After this time the reaction mixture was diluted with CH_2Cl_2 and the organic phase was washed with 2M $NaHSO_3$ aqueous solution, then with saturated $NaHCO_3$ aqueous solution and in the end with brine. The organic phase was dried over Na_2SO_4 and concentrated under reduced pressure to afford the crude, which was purified by automatic chromatography (Biotage Sfär 10 g: nHex/EtOAc gradient 5 to 80 % in 8 CV) to afford the final product **20g** (y = 43%). TLC R_f (CH_2Cl_2/MeOH: 97/3): 0.81. MS (ESI) calculated for $C_{33}H_{45}N_3O_{11}$ $[M + Na]^+$ m/z: 682.30; found: 682.57.^{1}H NMR (400 MHz, $CDCl_3$) δ = 7.42 (d, *J* = 8.1 Hz, 2H, *CH Ar*), 7.24 (d, *J* = 8.0 Hz, 2H, *CH Ar*), 5.50 (dd, *J* = 10.0 Hz, *J* = 3.5 Hz, 1H, *H-2*), 5.32 (m, 1H, *H-4*), 5.08 (dd, *J* = 10.2, *J* = 3.4 Hz, 1H, *H-3*), 4.73 (s, 2H, *CH₂*), 4.39 (d, *J* = 9.8 Hz, 1H, *H-1*), 3.88 (q, *J* = 6.5 Hz, 1H, *H-5*), 2.91 (s, 3H, *N-CH₃*), 2.22 (s, 3H, *OAc*), 2.09 (s, 3H, *OAc*), 2.03 (s, 3H, *OAc*), 1.52 (s, 18H, *tBu*), 1.27 (d, *J* = 5.6 Hz, 3H, *CH₃*). ^{13}C

NMR (100 MHz, $CDCl_3$): δ =170.8, 170.4, 169.6 (C=O), 132.3 (CH Ar), 128.3 (CH Ar), 86.4 (C1-C≡), 83.5 (≡C-Ar), 73.3 (C5), 71.9 (C3), 70.5 (C4), 69.8 (C1), 68.8 (C2), 54.9 (CH_2), 35.0 (CH_3), 28.8 (C-<u>CH3</u>), 20.9, 20.9, 20.8 (CH_3 OAc), 16.6 (C6).

1.10.3. Synthesis of ***N',N''*-bis(*tert*-butoxycarbonyl)-1-(4-(β-L-fucopyranosylethynyl)benzyl-*N*-methylguanidine 21g**

***N', N''*-bis(*tert*-butoxycarbonyl) 1-(4-(2,3,4-tri-*O*-acetyl β-L-fucopyranosylethynyl)benzyl)-*N*-methylguanidine 20g** (0.076 mmol) was deacetylated according to general procedure A affording the crude, which was purified by automatic chromatography (Biotage Sfär 5g: CH_2Cl_2/MeOH gradient 0 to 25 % in 15 CV) to afford the final product **21g** (y = 54%). TLC R_f (CH_2Cl_2/MeOH: 9/1): 0.46. MS (ESI) calculated for $C_{27}H_{39}N_3O_8$ $[M + Na]^+$ m/z: 556.26; found: 556.55. ^1H NMR (400 MHz, MeOD) δ = 7.35 (d, J = 8.2 Hz, 2H, *CH Ar*), 7.17 (d, J = 8.1 Hz, 2H, *CH Ar*), 4.56 (bs, 2H, *CH_2*), 3.97 (d, J = 9.6 Hz, 1H, *H-1*), 3.63-3.54 (m, 3H, *H-2* + *H-4* + *H-5*), 3.36 (dd, J = 9.4, J = 3.3 Hz, 1H, *H-3*), 2.82 (bd, 3H, *N-CH_3*), 1.37 (s, 18H, *tBu*), 1.18 (d, J = 6.5 Hz, 3H, *CH_3*).^{13}C chemical shifts were extrapolated from the HSQC experiment: δ = 131.6, 127.2 (CH Ar), 75.0 (C3), 74.6 and 71.0 (C4 and C5), 71.4 (C1), 70.7 (C2), 53.0 (CH_2), 35.3 (CH_3), 27.1 (CH_3 tBu), 15.1 (C6).

1.10.4. Synthesis of **1-(4-(β-L-fucopyranosylethynyl)benzyl)-*N*-methylguanidine 22g**

The Boc-protected compound **21g** (0.02 mmol, 1.0 eq) was deprotected according to general procedure C, affording **22g** as a TFA salt (quantitative yield). TLC Rf (CH_2Cl_2/MeOH: 85/15): 0.19. MS (ESI) calculated for $C_{17}H_{23}N_3O_4$ $[M + H]^+$ m/z: 334.18; found: 334.5. HRMS (ESI^+-TOF) calculated for $C_{17}H_{23}N_3O_4$ $[M + H]^+$ m/z: 334.1767, found: 334.1764. $[\alpha]_D^{24}$ = 2.2 (MeOH). ^1H NMR (400 MHz, MeOD): δ = 7.52 (d, J = 8.2 Hz, 2H, *CH Ar*), 7.24 (d, J = 8.0 Hz, 2H, *CH Ar*), 4.63 (s, 2H, *CH_2*), 4.08 (d, J_{1-2} = 9.8 Hz, 1H, *H-1*), 3.75-3.63 (mult, 3H, *H-2, H-4, H-5*), 3.47 (dd, J_{3-2} = 10.6 Hz, J_{3-4} = 3.4 Hz, 1H, *H-3*), 3.03 (s, 3H, *N-CH_3*), 1.29 (d, J_{CH3-5} = 6.4 Hz, 3H, *CH_3*). ^{13}C NMR (100 MHz, MeOD):δ = 157.7 (*C guanidine*), 135.4 (*C Ar*), 132.3 (*CH Ar*), 128.3 (*CH Ar*), 122.5 (*C Ar*), 86.4 (*C1-<u>C</u>≡*), 83.5 (*≡<u>C</u>-Ar*), 74.80 (*C2*), 70.7 (*C5*), 71.9 (*C3*), 71.8 (*C4*), 71.4 (*C1*), 53.4 (*CH_2*), 36.1 (*CH_3*), 15.7 (*C6*).

2. Ligand design

2.1. Ligand structure and selection

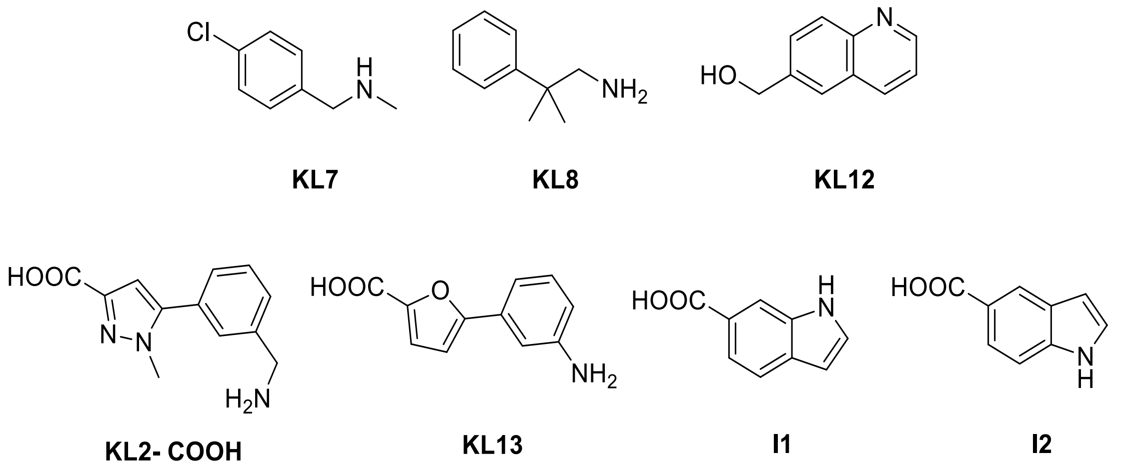

Figure S1. Fragments employed in the design of the bifunctional ligands for the BC2L-C N-terminal domain. The fragment numbers derive from Lal and co-workers[15]

Glycomimetic bifunctional ligands were designed starting from some of the fragments previously validated from the virtual screening.[15] In particular, the first generation ligands were designed around **KL7** and **KL8** (**Figure S1**), which are among the best fragments for the vicinal ligandable site in the two water docking model described in the previous work,[15] and around **KL12**, which was predicted to bind in the same site in the one water docking model.[15]

The fragments **KL13, I1** and **I2** used for the synthesis of amides **8c-e** were identified from a similarity search based on Tanimoto coefficient (>95%) in the PubChem database, using as the query structure fragment **KL2**[15] endowed with a COOH group (**KL2-COOH** in **Figure S1**), or from an additional search performed around a fused heterobicyclic scaffold revealed by the first expansion.

2.2. Virtual screening of fragments and glycomimetic ligands

All the docking calculations were performed using the Schrödinger Suite through Maestro (version 2018-1) graphical interface.[16] The docking of fragments was performed using the protocol described previously.[15] The model used to perform docking studies of the glycomimetic ligands is described below.

2.3. Protein preparation

Atomic coordinates of crystal structure of BC2L-C-nt with MeSe-α-L-Fuc (PDB code 2WQ4) were taken from the Protein Data Bank.[17] The asymmetric unit involves three peptide chains with three identical carbohydrate ligands (MeSe-α-L-Fuc) around a 3-fold pseudo axis of symmetry. The sugar at the three binding sites displays identical binding pose, therefore only one binding site (located between chains A and C) was used for docking calculations. The structural water molecules HOH2195 (W1) and HOH2194 (W2) were retained in the binding site region. The hydrogen atoms were added and pKa was calculated for protein residues using the PROPKA method [18–20] at pH 7.4. The HIE protonation state was also assigned to histidine (His116) residue. Thereafter, the protein-

ligand complex was minimized by applying convergence of heavy atoms to RMSD of 0.3 Å using the OPLS3 force field.[21]

2.4. Ligand preparation

The glycomimetic ligands were prepared for docking using the LigPrep tool.[22] The protonation states were generated at pH 7±2.

2.5. Receptor grid for docking study

The docking grid was prepared without fucose while retaining the two water molecules (W1 and W2) mentioned above. The centroid of fucoside was located in the active site between chain A and chain C in order to define a cubic grid box with dimensions 32×32×32 Å.

2.6. Ligand docking

The selenium atom in crystal structure (MeSe-α-L-Fuc) was replaced by oxygen and the metylfucoside was redocked at the sugar binding site. The protocol reproduced the co-crystallized pose (RMSD 0.1 Å), hence validating the docking protocol using Glide (version 7.8).[23] The glycomimetic ligands designed using the best fragments from virtual screening were studied using XP and SP approaches in Glide.[23] The docking results for the molecules with alkyne, amide, *E*-alkene and a 1,4-triazole linkers indicate that the sugar and the non-sugar part of the glycomimetic ligands establish interactions with the key residues already identified by the docking studies of the fragments.[15] The main interactions between the ligands and the protein involve π-π stacking with Tyr58 and the salt bridge or H-bond interactions between ammonium or aniline group and Asp70 (O) in the site X. Moreover, the amide linker forms additional H-bonding interactions with the structurally conserved water molecule W2. Thus, the expected binding pose with all the key interactions were maintained in the docking studies which was again confirmed by experimental studies using X-ray crystallography (**Figure S2 and S3**).

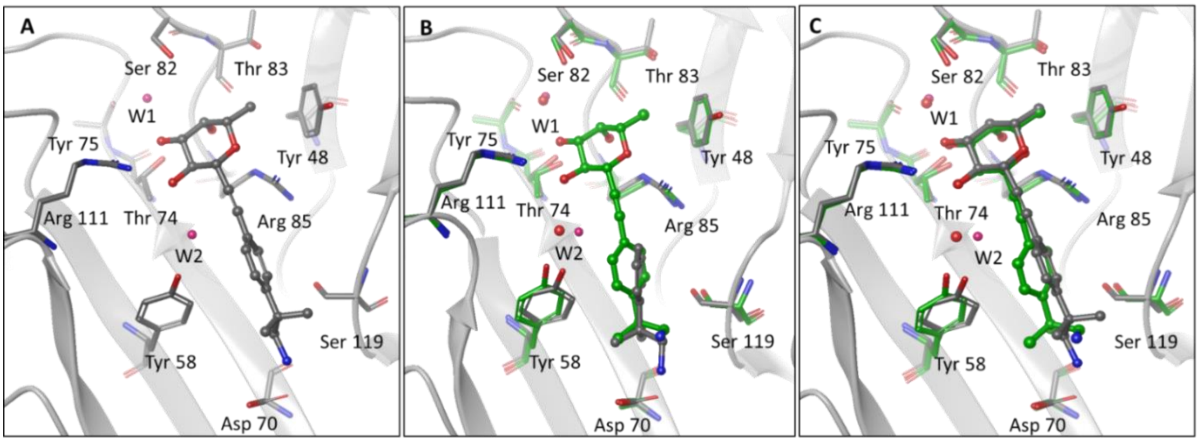

Figure S2. Comparison of crystal structures and docking predictions for ligand **22a.** (A) Docked pose of ligand **22a** in BC2L-C-Nter. (B) Superimposition of the crystal structure of the **22a** complex (green) with docking pose of fragment **KL8** (grey).[15](C) Superimposition of the crystal structure (green) and the docked pose (grey) of the **22a** complex. Water molecules are depicted as red (crystal) or pink (docking model) spheres.

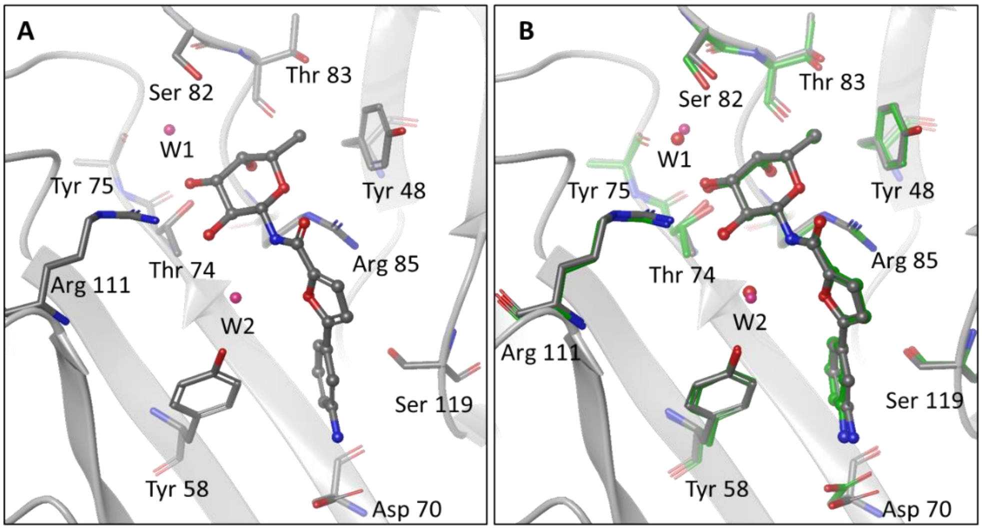

Figure S3. Comparison of crystal structures and docking predictions for ligand **8c**. (A) Docked pose of ligand **8c** in BC2L-C-Nter. (B). Superimposition of crystal structures (green) with docking pose (grey). Water molecules are depicted as red (crystal) or pink (docking model) spheres.

3. Ligand evaluation materials and methods

3.1. Isothermal Titration Calorimetry

All experiments were performed at 25 °C with an ITC200 isothermal titration calorimeter (Microcal-Malvern Panalytical, Orsay, France). The protein rBC2L-CN2 and its ligands were dissolved in a buffer composed of 20 mM Tris HCl pH 7.0 and 100 mM NaCl. The 200 µL sample cell containing rBC2L-CN (concentrations ranging from 200 to 400 µM) was subjected to injections of ligand solution: 20 to 39 injections of 1 µL or 70 injections of 0.5 µL (5 to 50 mM, chosen depending on the ligand) at intervals of 100, 120 or 200s while stirring at 850 rpm. Control experiments were performed by repeating the same protocol, but injecting the ligand into buffer solution. The supplied software Origin 7 or MicroCal PEAQ-ITC was used to fit the experimental data to a theoretical titration curve allowing the determination of affinity (i.e., association constant, K_a), binding enthalpy (ΔH), and stoichiometry (n). Values for free energy change (ΔG) and entropy contributions ($T\Delta S$) were derived from the equation $\Delta G = \Delta H - T\Delta S = - RT \ln K_a$ (with T = 298.15 K and R = 8.314 $J.mol^{-1}.K^{-1}$. For experiments in ligand excess, the stoichiometry was fixed to 1.

3.2. Surface plasmon Resonance

Experiments were performed on a BIACORE X100 instrument (GE Healthcare) at 25 °C in running buffer 10 mM HEPES pH 7.4, 150 mM NaCl and 0.05% Tween 20, adjusted to include 8% DMSO when indicated. rBC2L-CN2 was immobilized onto CM5 chips (BIACORE) following the amine coupling procedure:

- Activation of the chip by three injections of a NHS/EDC mixture at 10 µL/min for 540s, until a minimum of 300 RU was observed on both channels.
- Injection of rBC2L-CN2 (0.5 mg/mL) dissolved in 10 mM sodium acetate pH 4.5 onto channel 2 (contact time of 540s, flow rate of 10 µL/min), until a minimum of 7000 RU was observed for rBC2L-CN2.
- Inactivation of both channels by injecting a 1M ethanolamine (pH 8.5) solution at 5 µL/min for 1080s, achieving over 400 and 7000 RU for channel 1 and 2, respectively.

The analytes were dissolved in the running buffer at increasing concentrations (range: 3.28 − 3500 µM) and subjected to multi-cycle affinity studies (300s association, 300s dissociation, flow rate 5 µL/min). Injections of compounds at increasing concentrations onto the immobilized rBC2L-CN2 were followed by regeneration of the surface: 10 mM fucose in running buffer, then running buffer at 5 µL/min (100s and 150s, respectively) after each analyte association/dissociation. For the higher concentrations, regeneration was secured by performing one or more runs replacing analyte by running buffer. Duplicates were performed for all ligands. Binding affinity (K_D) was measured after subtracting the channel 1 reference (no immobilized protein) and subtracting of a blank injection (running buffer - zero analyte concentration). Data evaluation and curve fitting was performed using the provided BIACORE X100 evaluation software (version 2.0). The protein-coated chip was stored at 4 °C in running buffer and was functional up to 8 weeks after fabrication, as proven by experimentation.

3.3. Differential Scanning Calorimetry

Experiments were performed on a Microcal PEAQ-DSC instrument (Malvern Panalytical, Orsay, France). A buffer composed of 20 mM Tris HCl pH 7.0 and 100 mM NaCl was used to dilute the

protein rBC2L-CN2 and its ligands to concentrations 14.3 μM and 143 μM, respectively. Samples of 250 μL were loaded, while the reference cell was filled with the matching buffer (aforementioned buffer, ligands when relevant). Each sample was subjected to a gradient of temperature from 20 to 130 °C, at a scan rate of 200 °C/hr, followed by a second similar gradient, generating a reference thermogram. The data was acquired on 'Low' feedback mode. The supplied software MicroCal PEAQ-DSC Software 1.53 was used to fit the experimental data. To obtain the final thermograms, each experiment had its reference thermogram subtracted, the 'Progress' baseline fitting method was used. The profile obtained was fitted with a 'NonTwoState' model, accounting for two thermal events. Each experiment was performed in duplicates and their averages were calculated by the software.

3.4. Saturation Transfer Difference - NMR

^{1}H STD-NMR spectra were acquired at 283 K on a Bruker AVANCE 600 MHz spectrometer. The protein and ligand were dissolved in phosphate buffer (Na_2HPO_4, KH_2PO_4) 20mM pH 7.4, 100 mM NaCl and 5% D_2O in a 3 mm NMR tube (160 μl). Ligand/protein ratios were adjusted to 1000:1 in molar concentration. Water suppression was achieved by using the WATERGATE 3-9-19 pulse sequence. The on-resonance irradiation of the protein was kept at -0.05 ppm and 10 ppm. Off-resonance irradiation was applied at 200 ppm, where no protein signals were visible. Selective pre-saturation of the protein was achieved by a train of Gauss shaped pulses of 49 ms length each. The experiments were acquired with a saturation time of 2.94 s

In **Figure S4** the blank STD experiment for **22a** acquired in absence of protein is shown. This excludes that direct irradiation effects may be responsible for the results shown in Figure 3.

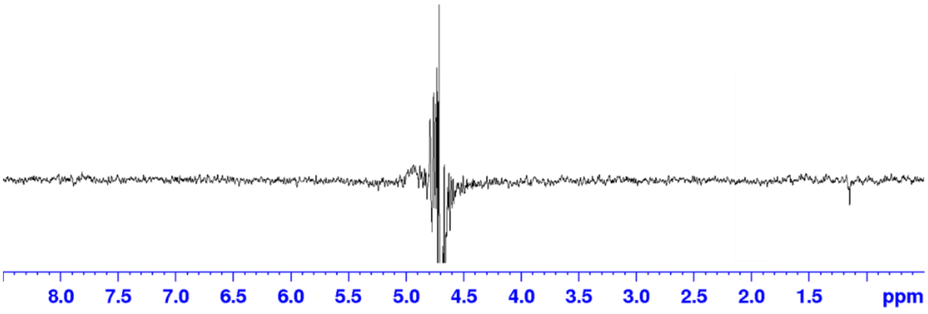

Figure S4. Blank STD experiment for **22a**, acquired in the absence of protein

.

3.5. Crystallization, Data Collection, and Structure Determination

Crystals of rBC2L-CN2 were obtained by 2 μl hanging drops and vapor diffusion using 1.2-1.3 M trisodium citrate at pH 7.0 at 19°C and the protein at 5 mg/ml in 20 mM Tris-HCl pH 7.0 and 100 mM NaCl, as previously described.[24] Cocrystals with H-type 1 tetrasaccharide were soaked overnight with 1.25 mM of compound **22a** (stock at 50 mM in protein buffer). Apo crystals were soaked for 5 h with 2 mM of compound **8c** (stock at 50 mM in 100 % DMSO). The crystals were then cryoprotected using 2.5 M sodium malonate at pH 5.0 and flash-cooled in liquid nitrogen. Data for BC2L-C-Nt in complex with compounds **22a** and **8c** were collected at: at the synchrotron SOLEIL, Saint Aubin, France on beamline Proxima 2 using an Eiger-9M detector (Dectris, Baden, Switzerland) and on beamline Proxima 1 using an Eiger-16M detector, respectively (see statistics in Table S4).

The data were processed using XDS and XDSME and then programs of the CCP4 suite were used.[25–27] The coordinates of protomer A of PDB-ID 2WQ4 were used as a search model to solve all new structures of rBC2L-CN2 by molecular replacement using PHASER.[28] Refinement was performed by multiple iterations of restrained maximum likelihood refinement and REFMAC 5.8 and manual rebuilding in Coot.[29,30] 5% of the observations was set aside for cross-validation analysis. Hydrogen atoms were added in their riding positions during refinement. A library for the synthetic molecules was created in the Coot ligand builder. The final model was validated using the wwPDB validation server, https://validate-rcsb-1.wwpdb.org/ and the carbohydrate conformations were checked using Privateer.[31] The coordinates were deposited in the Protein Data Bank (PDB) under codes 7OLU and 7OLW for structures in complex with **22a** and **8c**, respectively.

Table S4. Data collection and refinement statistics. Values in parentheses for highest-resolution shell.

Data Collection	BC2L-C-N_t/22a	BC2L-C-N_t/8c
Beamline	Proxima 2 (Soleil)	Proxima 1 (Soleil)
Wavelength	0.98011	0.97857
Space group	$P6_3$	$P6_3$
Unit cell dimensions (Å)	a = b = 44.0, c = 94.1	a = b = 42.9, c = 94.9
Resolution (Å)	47.07-1.79 (1.83-1.79)	47.44-1.32 (1.34-1.32)
Nb/nb unique reflections	196,154 / 9,696	470,817 / 23,283
R_{merge}	0.039 (0.228)	0.054 (0.480)
R_{meas}	0.040 (0.244)	0.057 (0.504)
Mean I/σI	49.1 (11.1)	30.6 (6.7)
Completeness (%)	99.70 (95.4)	100.0 (100.0)
Redundancy	20.2 (17.2)	20.2 (20.1)
$CC_{1/2}$	1.000 (0.990)	1.000 (0.959)
Refinement		
Resolution (Å)	38.07-1.79	37.18-1.32
Nb/nb free. reflections	9,670 / 454	23,246 / 1,163
R_{work}/R_{free}	14.0 / 19.4	10. 3/ 12.9
Rmsd bond lengths (Å)	0.016	0.012
Rmsd bond angles (°)	1.82	1.59
Rmsd chiral ($Å^3$)	0.090	0.082
No. atoms/Bfac ($Å^2$):		
Protein	978 / 27.3	1,055 / 14.8
Ligand	23 / 30.0	25 / 13.2
Waters	100 / 34.6	146 / 26.7
Ramachandran<br>Allowed / Favored / Outliers (%)	100 / 97.7 / 0	100 /97.4 / 0
PDB ID	7OLU	(7OLW

4. References

(1) Palomo, C.; Aizpurua, J. M.; Balentová, E.; Azcune, I.; Santos, J. I.; Jiménez-Barbero, J.; Cañada, J.; Miranda, J. I. "Click" Saccharide/β-Lactam Hybrids for Lectin Inhibition. *Org. Lett.* **2008**, *10* (11), 2227–2230. https://doi.org/10.1021/ol8006259.

(2) Nishi, Y.; Tanimoto, T. Preparation and Characterization of Branched β-Cyclodextrins Having α-L-Fucopyranose and a Study of Their Functions. *Biosci. Biotechnol. Biochem.* **2009**, *73* (3), 562–569. https://doi.org/10.1271/bbb.80609.

(3) Fusaro, M. B.; Chagnault, V.; Josse, S.; Postel, D. Metal-Free Oxidative Lactonization of Carbohydrates Using Molecular Iodine. *Tetrahedron* **2013**, *69* (29), 5880–5883. https://doi.org/10.1016/j.tet.2013.05.021.

(4) Frédéric, C. J. M.; Tikad, A.; Fu, J.; Pan, W.; Zheng, R. B.; Koizumi, A.; Xue, X.; Lowary, T. L.; Vincent, S. P. Synthesis of Unprecedented Sulfonylated Phosphono-Exo-Glycals Designed as Inhibitors of the Three Mycobacterial Galactofuranose Processing Enzymes. *Chem. - A Eur. J.* **2016**, *22* (44), 15913–15920. https://doi.org/10.1002/chem.201603161.

(5) Lowary, T.; Meldal, M.; Helmboldt, A.; Vasella, A.; Bock, K. Novel Type of Rigid C-Linked Glycosylacetylene-Phenylalanine Building Blocks for Combinatorial Synthesis of C-Linked Glycopeptides. *J. Org. Chem.* **1998**, *63* (26), 9657–9668. https://doi.org/10.1021/jo980517h.

(6) Dondoni, A.; Mariotti, G.; Marra, A. Synthesis of α- and β-Glycosyl Asparagine Ethylene Isosteres (C-Glycosyl Asparagines) via Sugar Acetylenes and Garner Aldehyde Coupling. *J. Org. Chem.* **2002**, *67* (13), 4475–4486. https://doi.org/10.1021/jo020054m.

(7) Alzeer, J.; Vasella, A. Oligosaccharide Analogues of Polysaccharides. Part 2. Regioselective Deprotection of Monosaccharide-derived Monomers and Dimers. *Helv. Chim. Acta* **1995**, *78* (1), 177–193. https://doi.org/10.1002/hlca.19950780117.

(8) Rouzier, F.; Sillé, R.; Nourry, A.; Tessier, A.; Pipelier, M.; Guillarme, S. Practical Gram-Scale Synthesis of Either α- Or β-Anomer of C -Vinyl Glycosides. *Synth.* **2019**, *51* (12), 2484–2488. https://doi.org/10.1055/s-0037-1611800.

(9) Cinelli, M. A.; Reidl, C. T.; Li, H.; Chreifi, G.; Poulos, T. L.; Silverman, R. B. First Contact: 7-Phenyl-2-Aminoquinolines, Potent and Selective Neuronal Nitric Oxide Synthase Inhibitors That Target an Isoform-Specific Aspartate. *J. Med. Chem.* **2020**, *63* (9), 4528–4554. https://doi.org/10.1021/acs.jmedchem.9b01573.

(10) Decréau, R. A.; Collman, J. P.; Yang, Y.; Yan, Y.; Devaraj, N. K. Syntheses of Hemoprotein Models That Can Be Covalently Attached onto Electrode Surfaces by Click Chemistry. *J. Org. Chem.* **2007**, *72* (8), 2794–2802. https://doi.org/10.1021/jo062349w.

(11) Le Baccon-Solliera, P.; Malkia, Y.; Mayea, M.; Ali, L. M. A.; Lichona, L.; Pierre, C.; Vincent, L.-A.; Nicolas, M. Imidazopyridine-Fused [1,3]Diazepinones: Modulations of Positions 2 to 4 and Their Impacts on the Anti-Melanoma Activity. *J. Enzyme Inhib. Med. Chem.* **2020**, *35* (1), 935–949.

(12) Bianchi, A.; Russo, A.; Bernardi, A. Neo-Glycoconjugates: Stereoselective Synthesis of α-Glycosyl Amides via Staudinger Ligation Reactions. *Tetrahedron Asymmetry* **2005**, *16* (2), 381–386. https://doi.org/10.1016/j.tetasy.2004.11.055.

(13) Kondor, Z.; Herczeg, M.; Borbás, A.; Patonay, T.; Kónya, K. Application of Carbohydrates with Methylene or Vinyl Groups in Heck-Mizoroki Cross-Coupling Reactions with O-Heterocycles. *Synlett* **2016**, *27* (19), 2709–2715. https://doi.org/10.1055/s-0036-1588591.

(14) Feichtinger, K.; Zapf, C.; Sings, H. L.; Goodman, M. Diprotected Triflylguanidines: A New Class of Guanidinylation Reagents. *J. Org. Chem.* **1998**, *63* (12), 3804–3805.

(15) Lal, K.; Bermeo, R.; Cramer, J.; Vasile, F.; Ernst, B.; Imberty, A.; Bernardi, A.; Varrot, A.;

Belvisi, L. Prediction and Validation of a Druggable Site on Virulence Factor of Drug Resistant Burkholderia Cenocepacia**. *Chem. - A Eur. J.* **2021**, *27* (40), 10341–10348. https://doi.org/10.1002/chem.202100252.

(16) Schrödinger Release 2018-1: Maestro, LLC, New York, NY, 2018.

(17) Berman, H. M.; Westbrook, J.; Feng, Z.; Gilliland, G.; Bhat, T. N.; Weissig, H.; Shindyalov, I. N.; E. Bourne, P. The Protein Data Bank. *Nucleic Acids Res.* **2000**, *28* (1), 235–242. https://doi.org/10.1038/s41577-020-00473-z.

(18) Olsson, M. H. M.; SØndergaard, C. R.; Rostkowski, M.; Jensen, J. H. PROPKA3: Consistent Treatment of Internal and Surface Residues in Empirical p K a Predictions. *J. Chem. Theory Comput.* **2011**, *7* (2), 525–537. https://doi.org/10.1021/ct100578z.

(19) Li, H.; Robertson, A. D.; Jensen, J. H. Very Fast Empirical Prediction and Rationalization of Protein PK a Values. *Proteins Struct. Funct. Genet.* **2005**, *61* (4), 704–721. https://doi.org/10.1002/prot.20660.

(20) Bas, D. C.; Rogers, D. M.; Jensen, J. H. Very Fast Prediction and Rationalization of PKa Values for Protein-Ligand Complexes. *Proteins Struct. Funct. Genet.* **2008**, *73* (3), 765–783. https://doi.org/10.1002/prot.22102.

(21) Harder, E.; Damm, W.; Maple, J.; Wu, C.; Reboul, M.; Xiang, J. Y.; Wang, L.; Lupyan, D.; Dahlgren, M. K.; Knight, J. L.; Kaus, J. W.; Cerutti, D. S.; Krilov, G.; Jorgensen, W. L.; Abel, R.; Friesner, R. A. OPLS3: A Force Field Providing Broad Coverage of Drug-like Small Molecules and Proteins. *J. Chem. Theory Comput.* **2016**, *12* (1), 281–296. https://doi.org/10.1021/acs.jctc.5b00864.

(22) Schrödinger Release 2018-1: LigPrep, LLC, New York, NY 2018.

(23) Halgren, T. A.; Murphy, R. B.; Friesner, R. A.; Beard, H. S.; Frye, L. L.; Pollard, W. T.; Banks, J. L. Glide: A New Approach for Rapid, Accurate Docking and Scoring. 2. Enrichment Factors in Database Screening. *J. Med. Chem.* **2004**, *47* (7), 1750–1759. https://doi.org/10.1021/jm030644s.

(24) Bermeo, R.; Bernardi, A.; Varrot, A. BC2L-C N-Terminal Lectin Domain Complexed with Histo Blood Group Oligosaccharides Provides New Structural Information. *Molecules* **2020**, *25* (2). https://doi.org/10.3390/molecules25020248.

(25) Legrand, P. XDSME: XDS Made Easier. GitHub Repos. 2017.

(26) Kabsch, W. Xds. Acta Crystallogr D Biol Crystallogr 2010, 66 (Pt 2), 125-32.

(27) Winn, M. D.; Ballard, C. C.; Cowtan, K. D.; Dodson, E. J.; Emsley, P.; Evans, P. R.; Keegan, R. M.; Krissinel, E. B.; Leslie, A. G. W.; McCoy, A.; McNicholas, S. J.; Murshudov, G. N.; Pannu, N. S.; Potterton, E. A.; Powell, H. R.; Read, R. J.; Vagin, A.; Wilson, K. S. Overview of the CCP4 Suite and Current Developments. *Acta Crystallogr. Sect. D Biol. Crystallogr.* **2011**, *67* (4), 235–242. https://doi.org/10.1107/S0907444910045749.

(28) McCoy, A. J. Solving Structures of Protein Complexes by Molecular Replacement with Phaser. *Acta Crystallogr. Sect. D Biol. Crystallogr.* **2006**, *63* (1), 32–41. https://doi.org/10.1107/S0907444906045975.

(29) Emsley, P.; Lohkamp, B.; Scott, W. G.; Cowtan, K. Features and Development of Coot. *Acta Crystallogr. Sect. D Biol. Crystallogr.* **2010**, *66* (4), 486–501. https://doi.org/10.1107/S0907444910007493.

(30) Murshudov, G. N.; Skubák, P.; Lebedev, A. A.; Pannu, N. S.; Steiner, R. A.; Nicholls, R. A.; Winn, M. D.; Long, F.; Vagin, A. A. REFMAC5 for the Refinement of Macromolecular Crystal Structures. *Acta Crystallogr. Sect. D Biol. Crystallogr.* **2011**, *67* (4), 355–367. https://doi.org/10.1107/S0907444911001314.

(31) Agirre, J.; Iglesias-Fernández, J.; Rovira, C.; Davies, G. J.; Wilson, K. S.; Cowtan, K. D.

Privateer: Software for the Conformational Validation of Carbohydrate Structures. *Nat. Struct. Mol. Biol.* **2015**, *22* (11), 833–834. https://doi.org/10.1038/nsmb.3115.

(2,3,4-tri-*O*-benzyl-β-L-fucopyranosyl) acetylene 3a

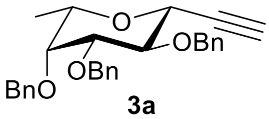

3a

^{1}H NMR (400 MHz, $CDCl_3$)

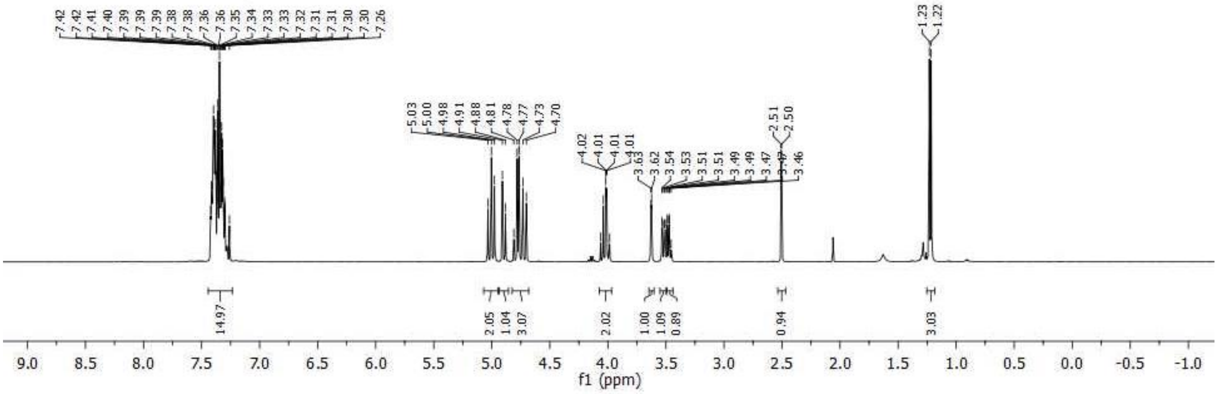

HSQC (400 MHz, $CDCl_3$)

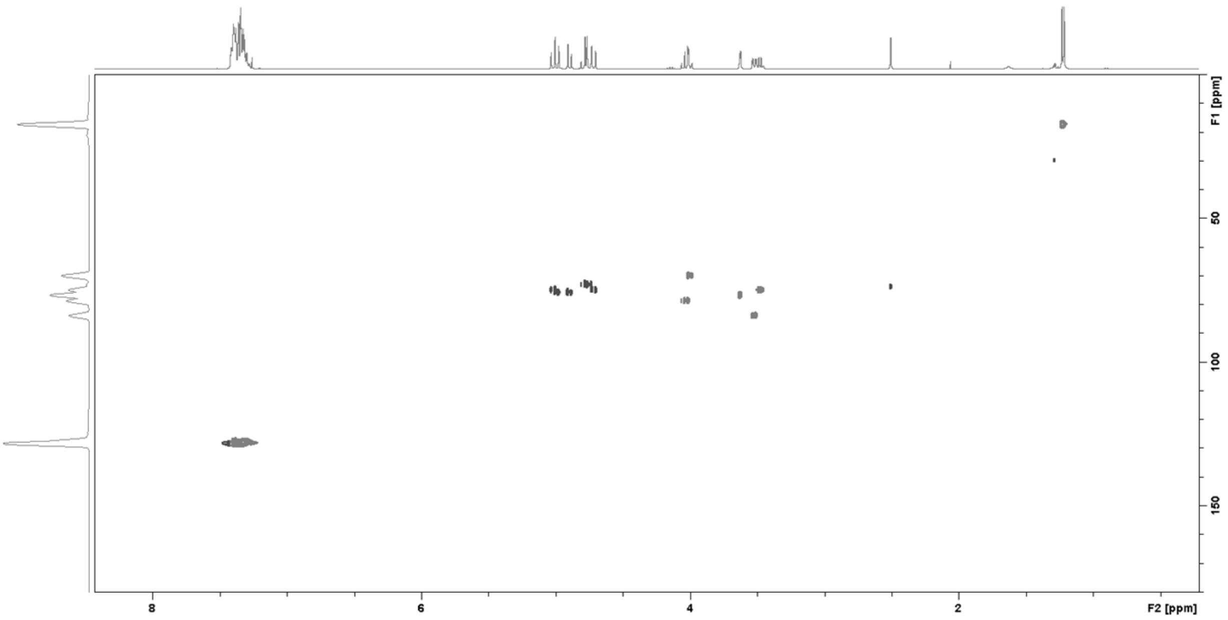

(2,3,4-tri-*O*-acetyl-β-L-fucopyranosyl) acetylene 3b

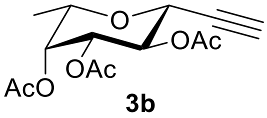

^{1}H NMR (400 MHz, CDCl$_3$)

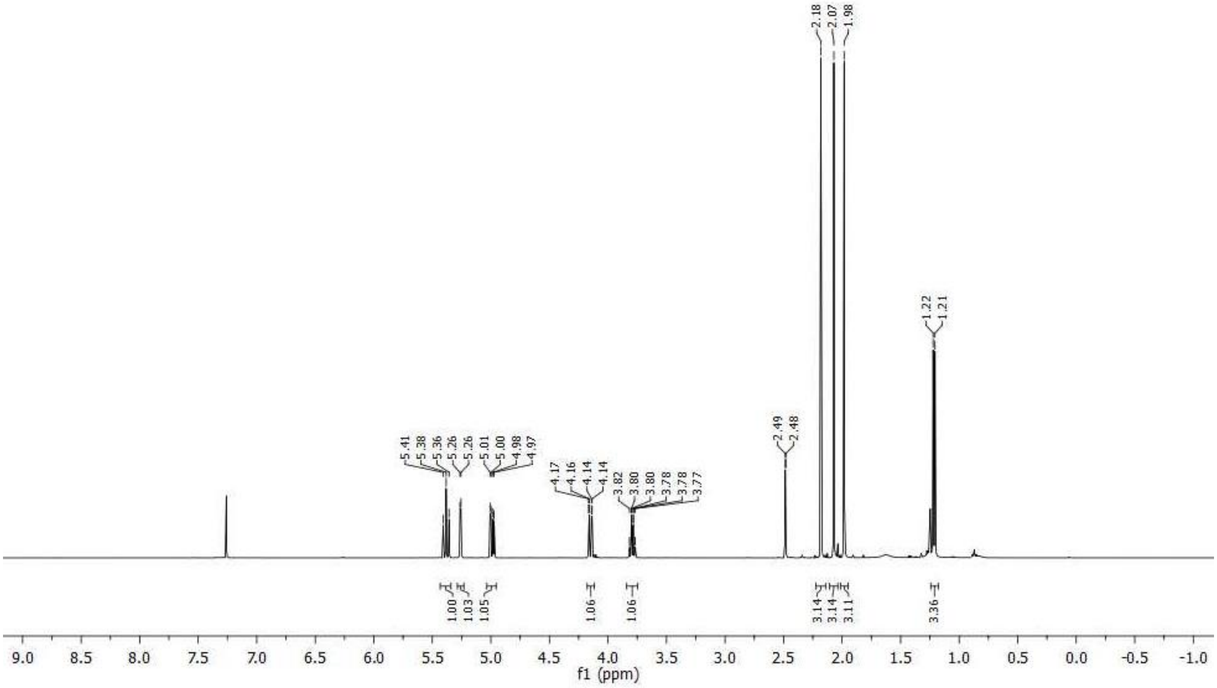

HSQC (400 MHz, CDCl$_3$)

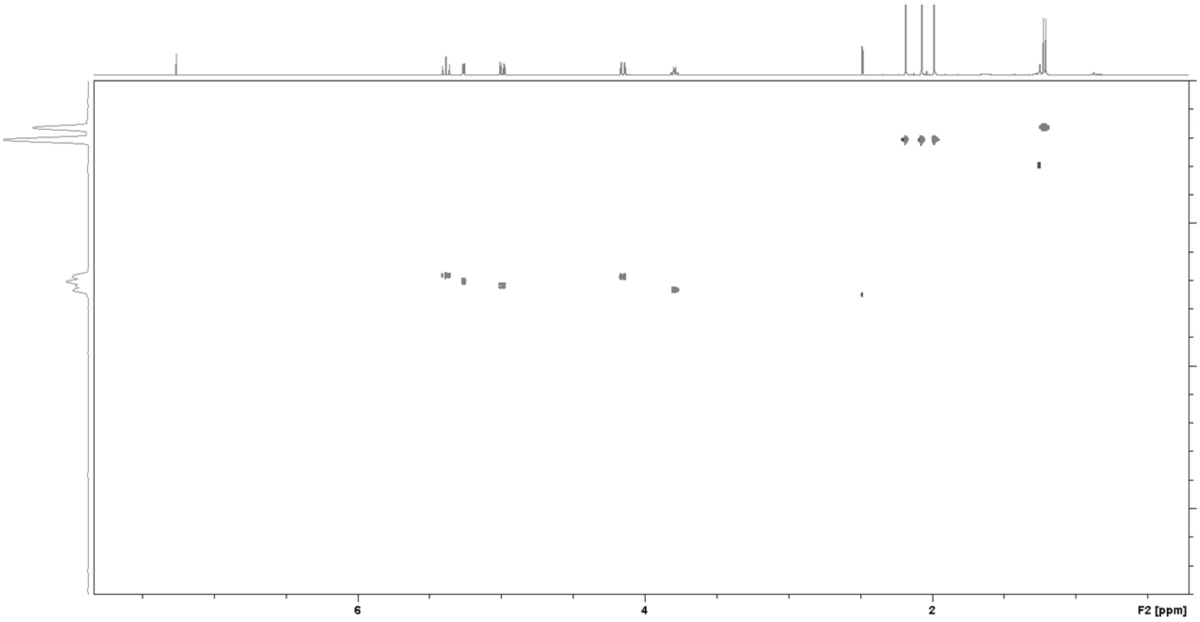

4-(1-amino-2-methylpropan-2-yl)-*N*-(β-L-fucopyranosyl) benzamide 8a

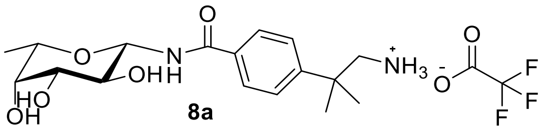

^{1}H NMR (400 MHz, CD_3OD)

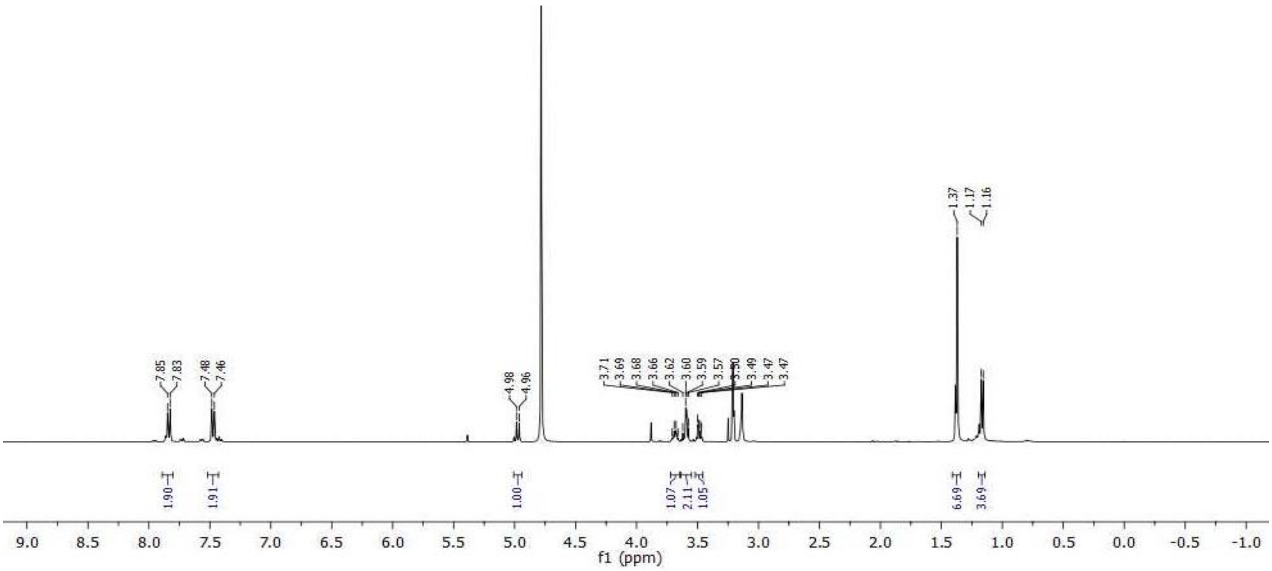

^{13}C NMR (100 MHz, CD_3OD)

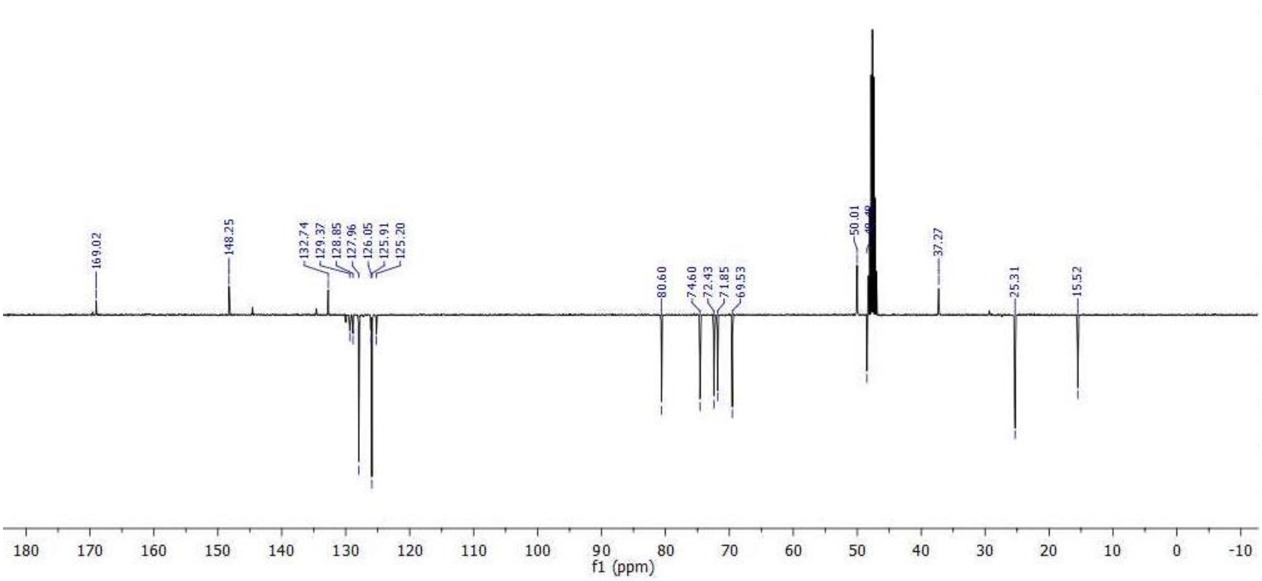

***N*-(β-L-fucopyranosyl)-4-((methylamino)methyl) benzamide 8b**

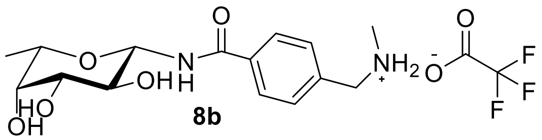

8b

^{1}H NMR (400 MHz, CD_3OD)

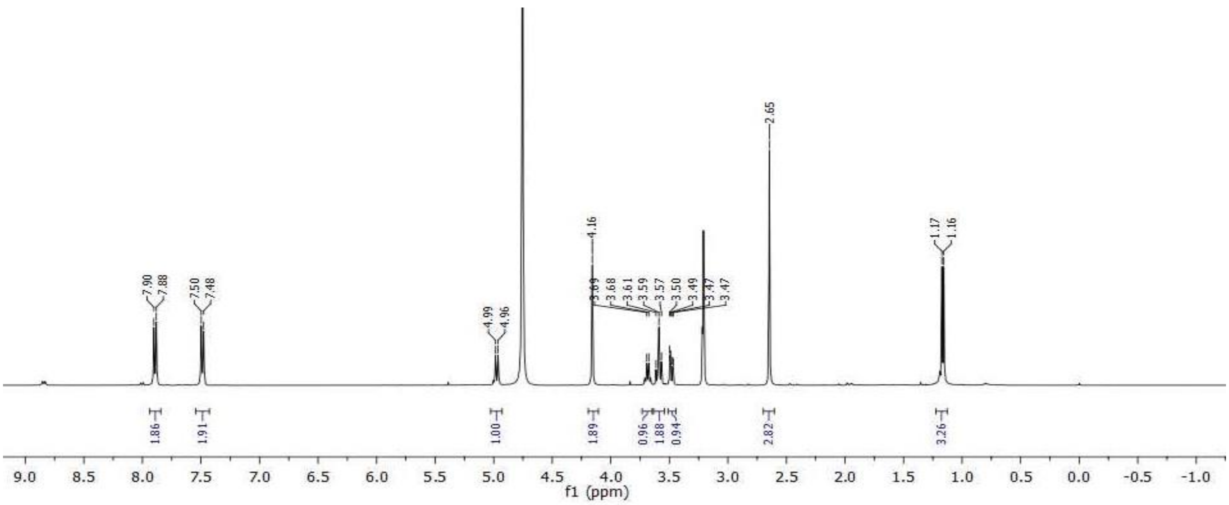

^{13}C NMR (100 MHz, CD_3OD)

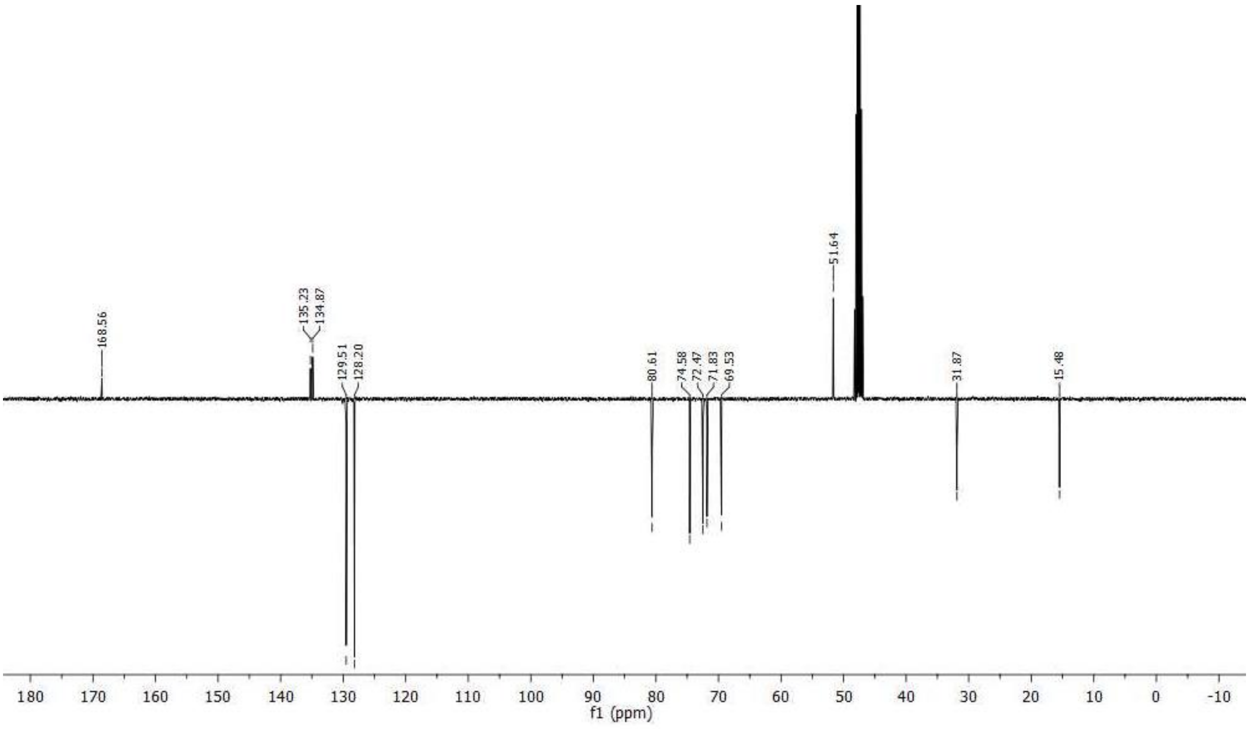

5-(3-Aminophenyl)furan-2-carboxamido-(β-L-fucopyranose) 8c

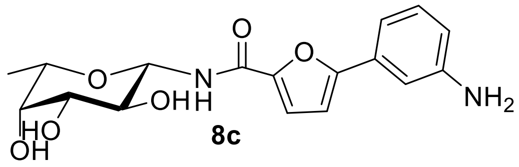

^{1}H NMR (400 MHz, DMSO-*d*6)

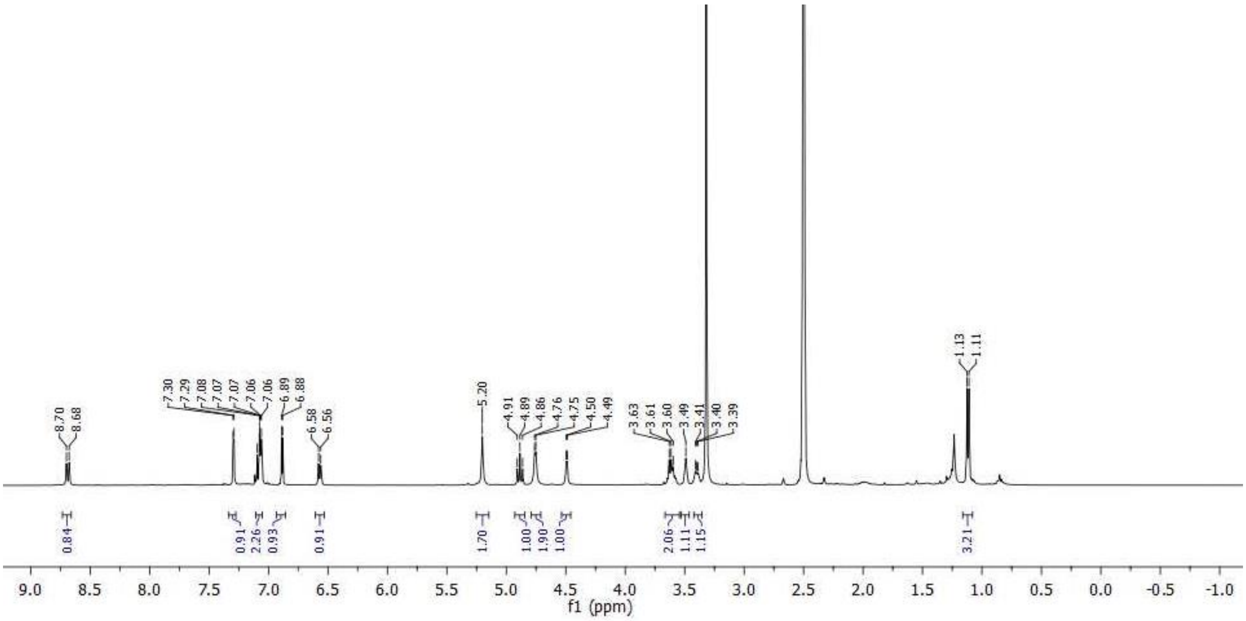

^{13}C NMR (100 MHz, DMSO-*d*6)

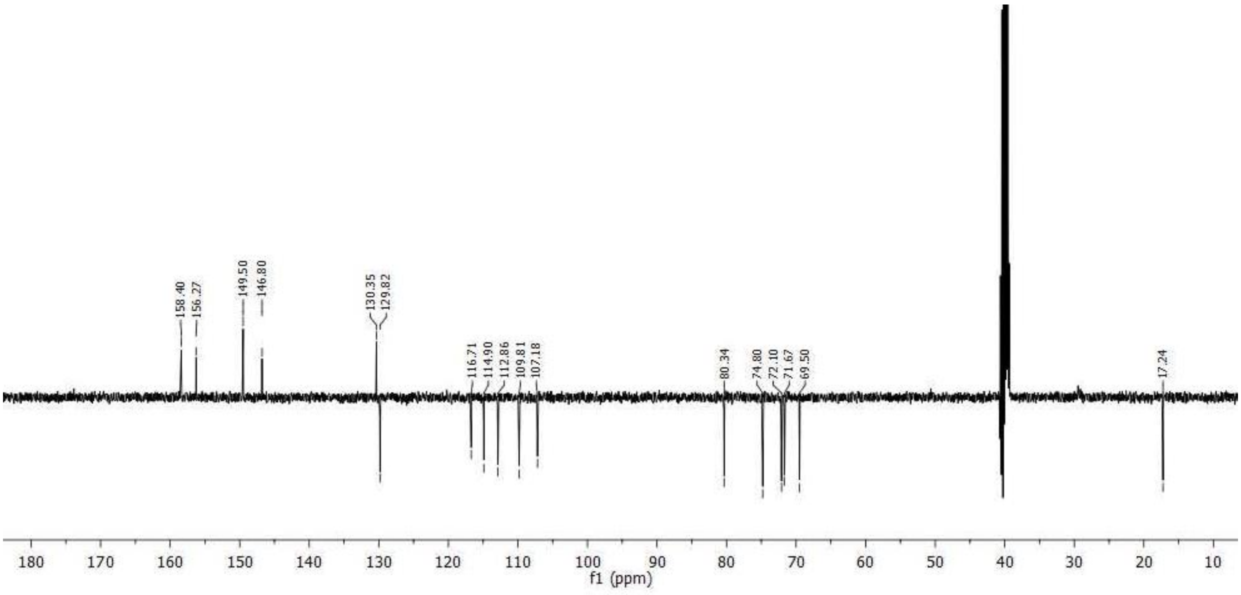

***N*-(β-L-fucopyranosyl)-1H-indole-6-carboxamide 8d**

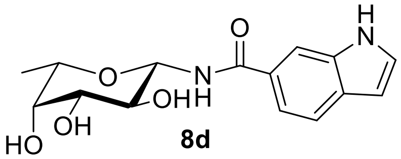

8d

^{1}H NMR (400 MHz, CD₃OD)

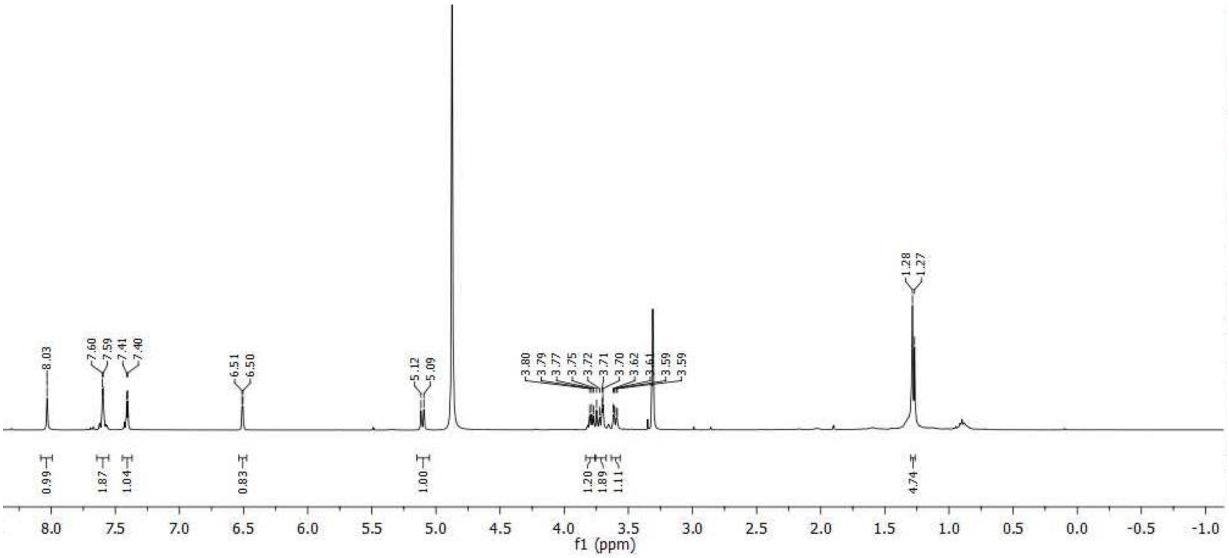

^{13}C NMR (100 MHz, CD₃OD)

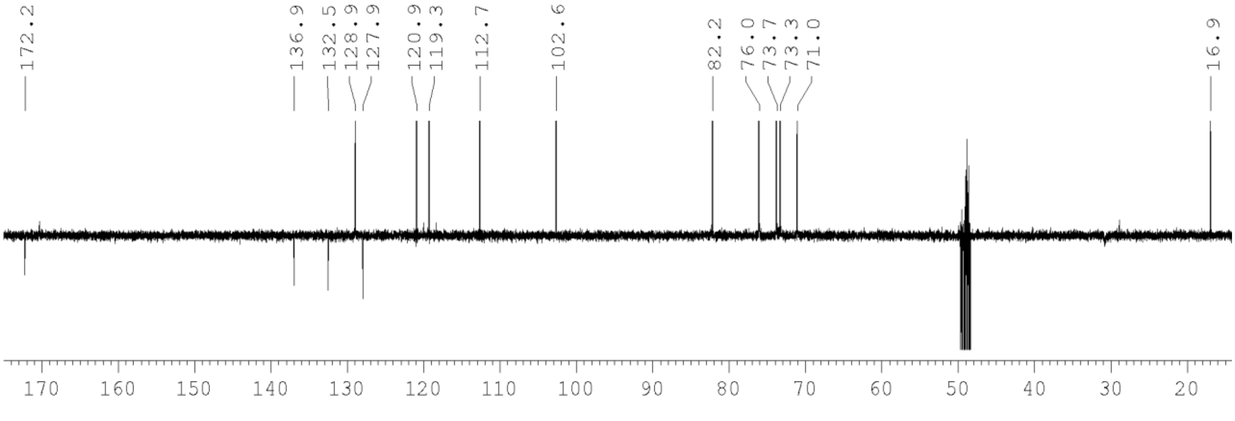

***N*-(β-L-fucopyranosyl)-1H-indole-5-carboxamide 8e**

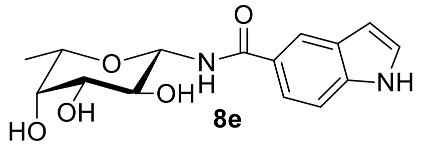

^{1}H NMR (400 MHz, CD_3OD)

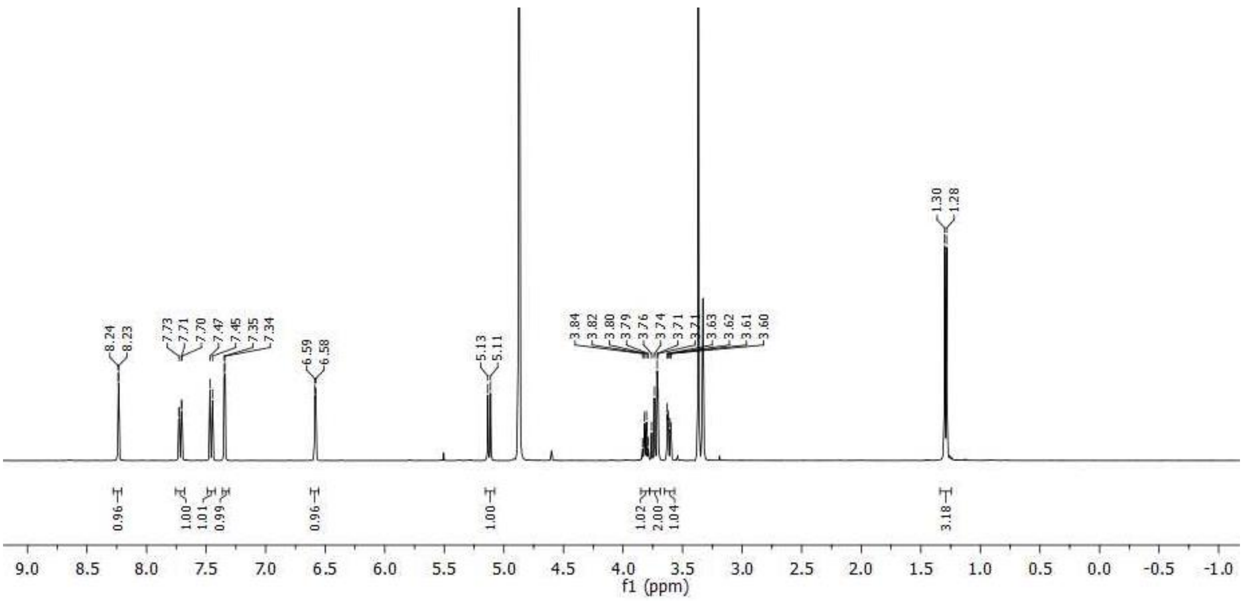

HSQC NMR (400 MHz, CD_3OD)

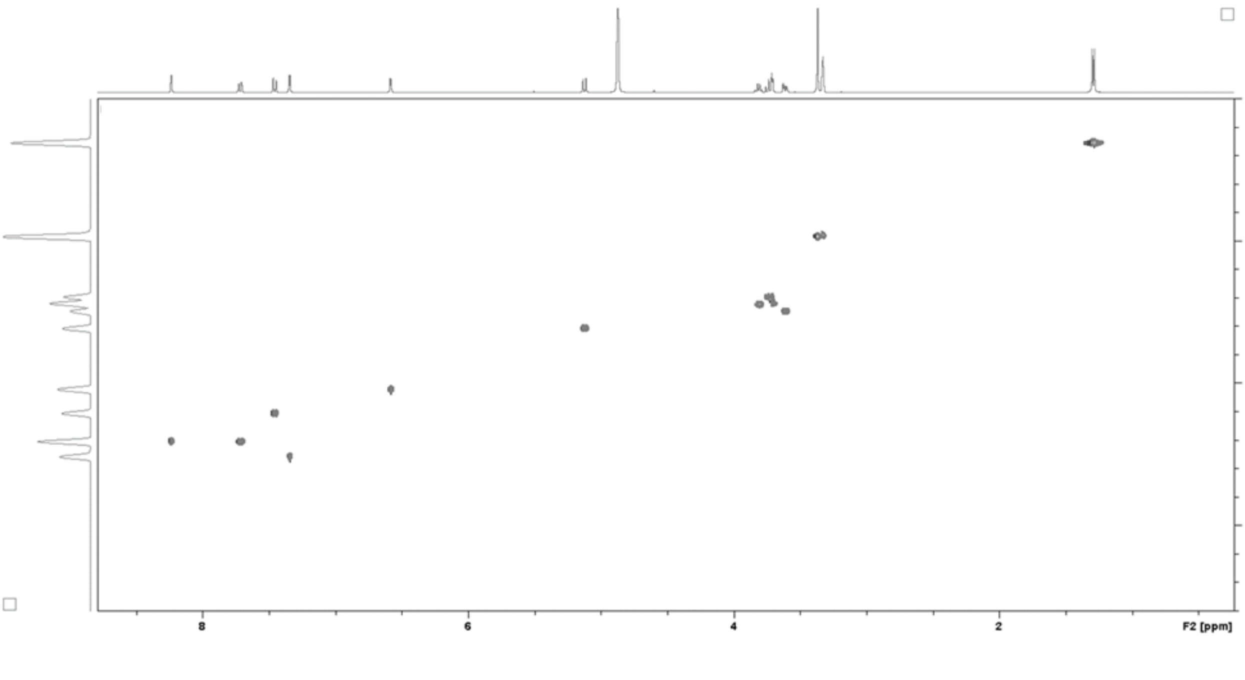

2-(4-(1-(β-L-fucopyranosyl)-1H-1,2,3-triazol-4-yl)phenyl)-2-methylpropan-1-amine 12a

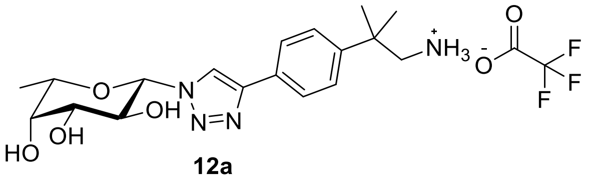

12a

^{1}H NMR (400 MHz, CD_3OD)

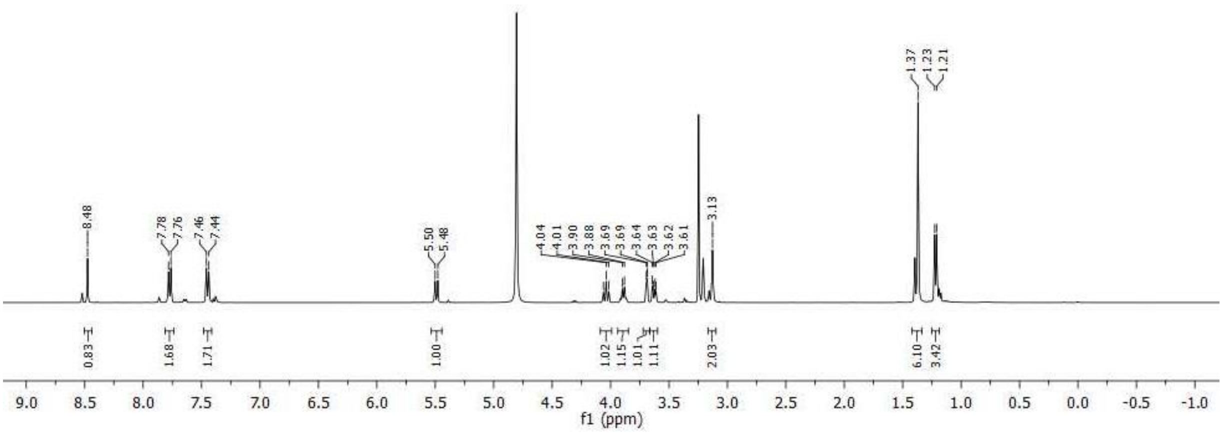

^{13}C NMR (100 MHz, CD_3OD)

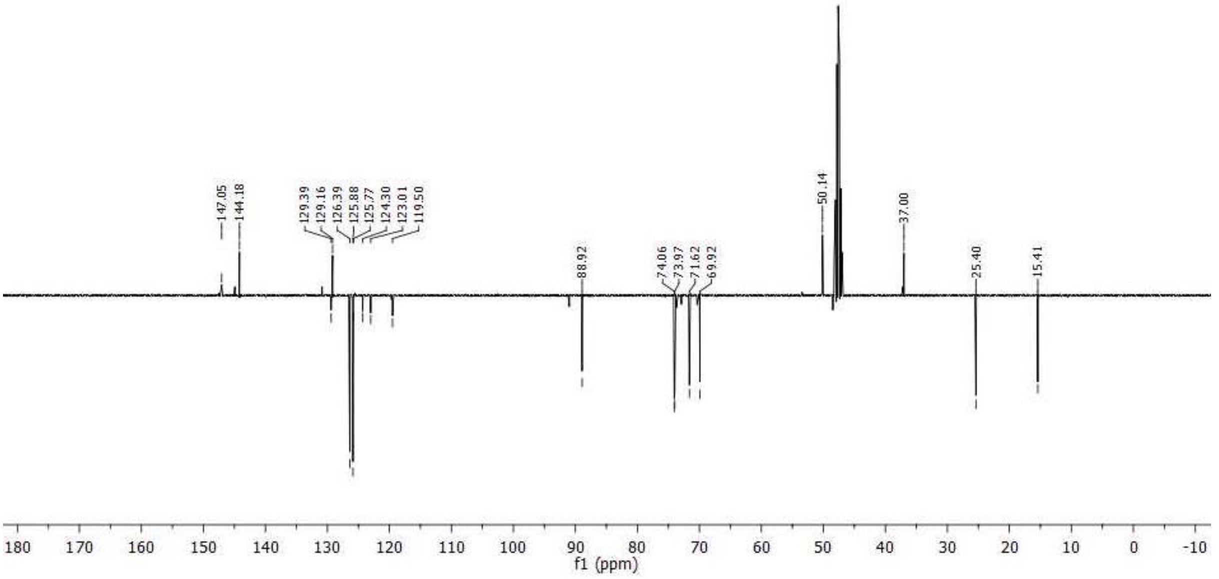

1-(4-(1-(β-L-fucopyranosyl)-1H-1,2,3-triazol-4-yl)phenyl)-N-methylmethanamine 12b

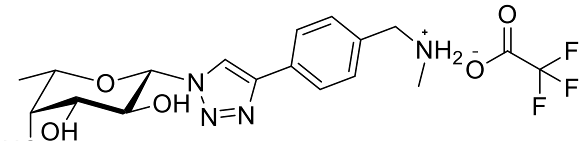

12b

^{1}H NMR (400 MHz, CD_3OD)

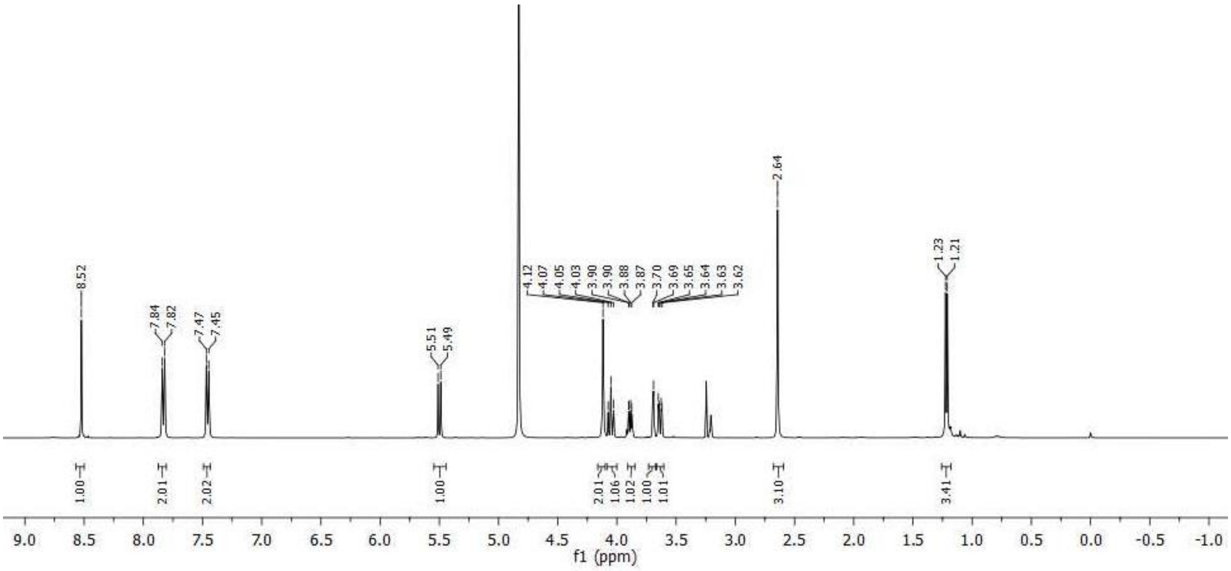

^{13}C NMR (100 MHz, CD_3OD)

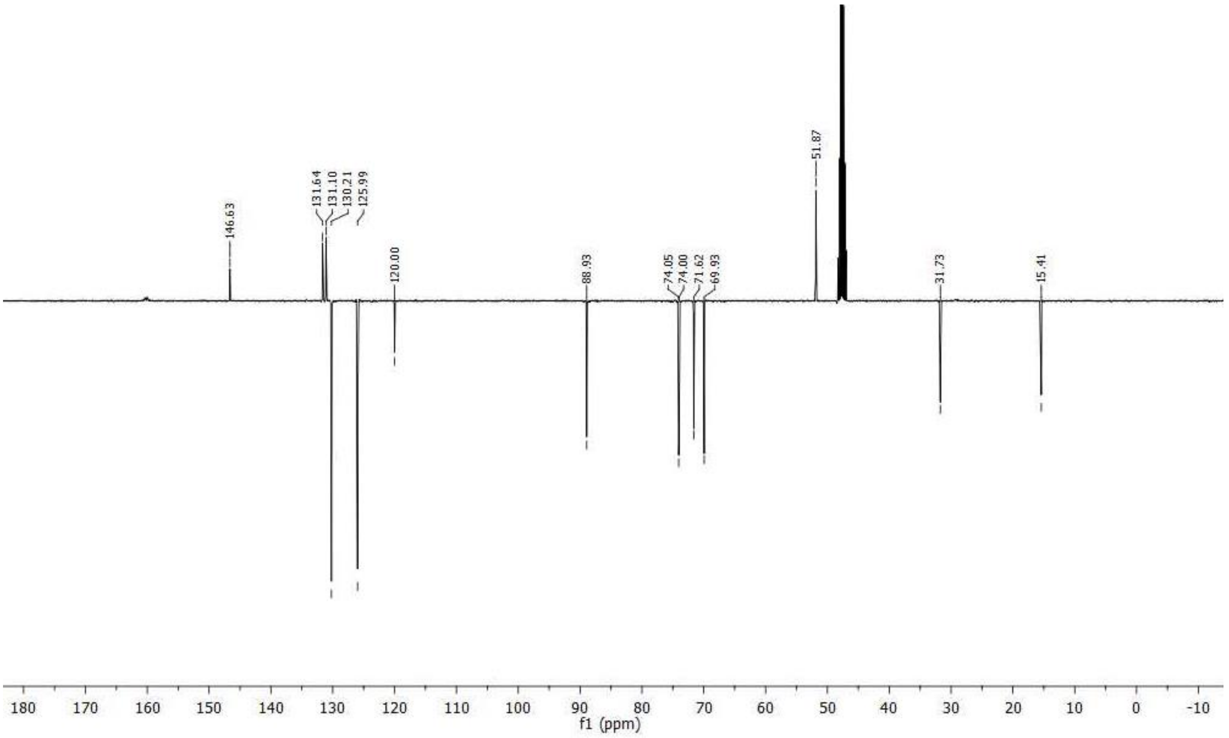

(2-(4-(β-L-fucopyranosylethynyl)phenyl)-2-methylpropan-1-amine 22a

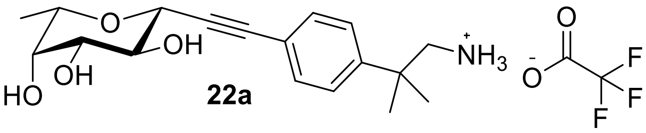

^{1}H NMR (400 MHz, CD_3OD)

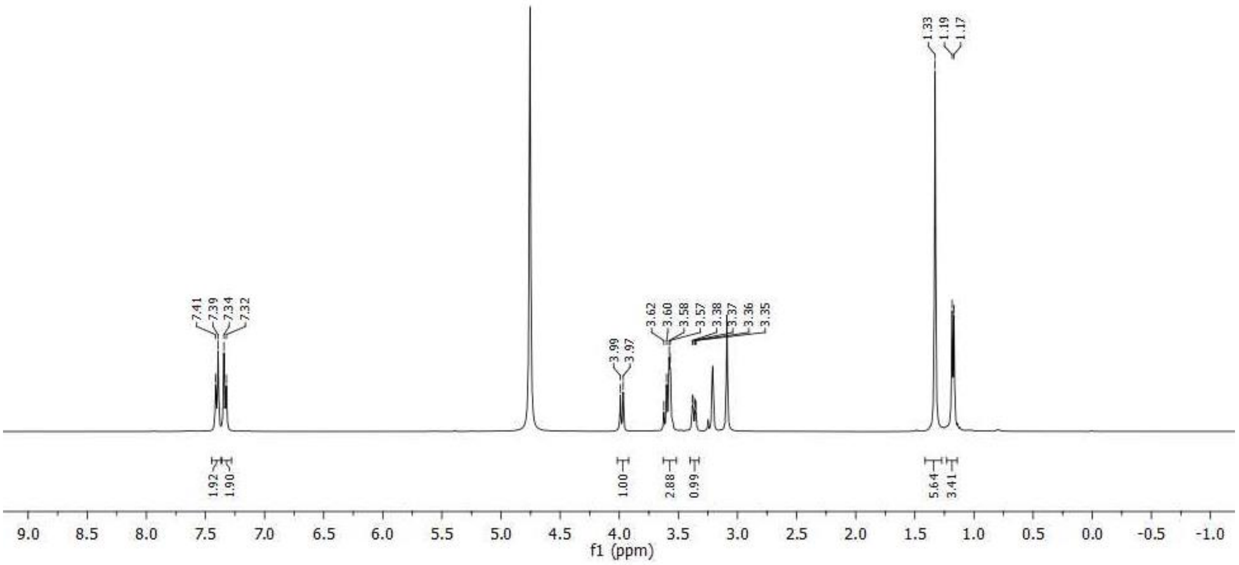

^{13}C NMR (100 MHz, CD_3OD)

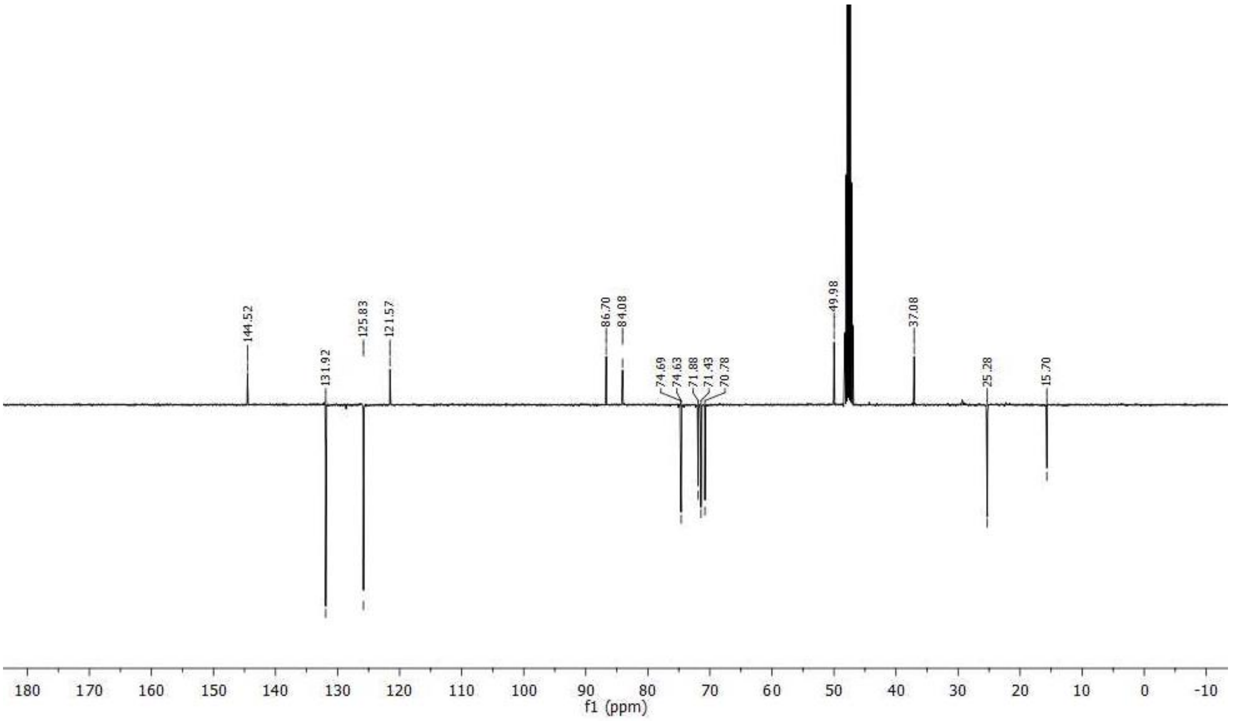

1-(4-(β-L-fucopyranosylethynyl)phenyl)-*N*-methylmethanamine 22b

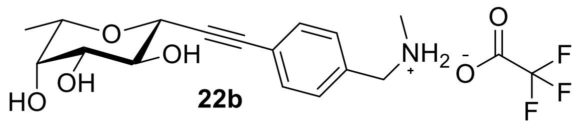

^{1}H NMR (400 MHz, CD_3OD)

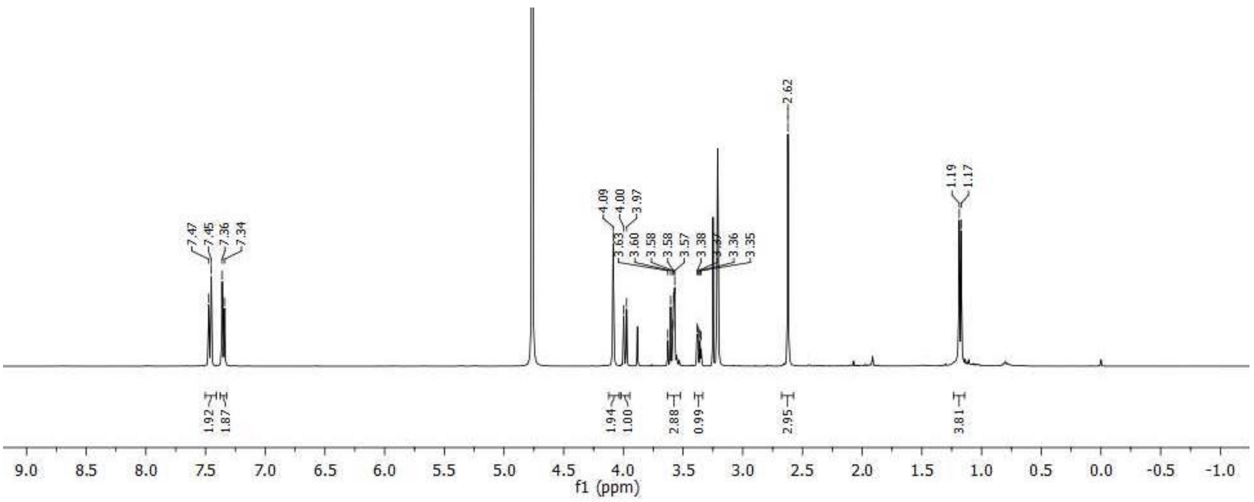

^{13}C NMR (100 MHz, CD_3OD)

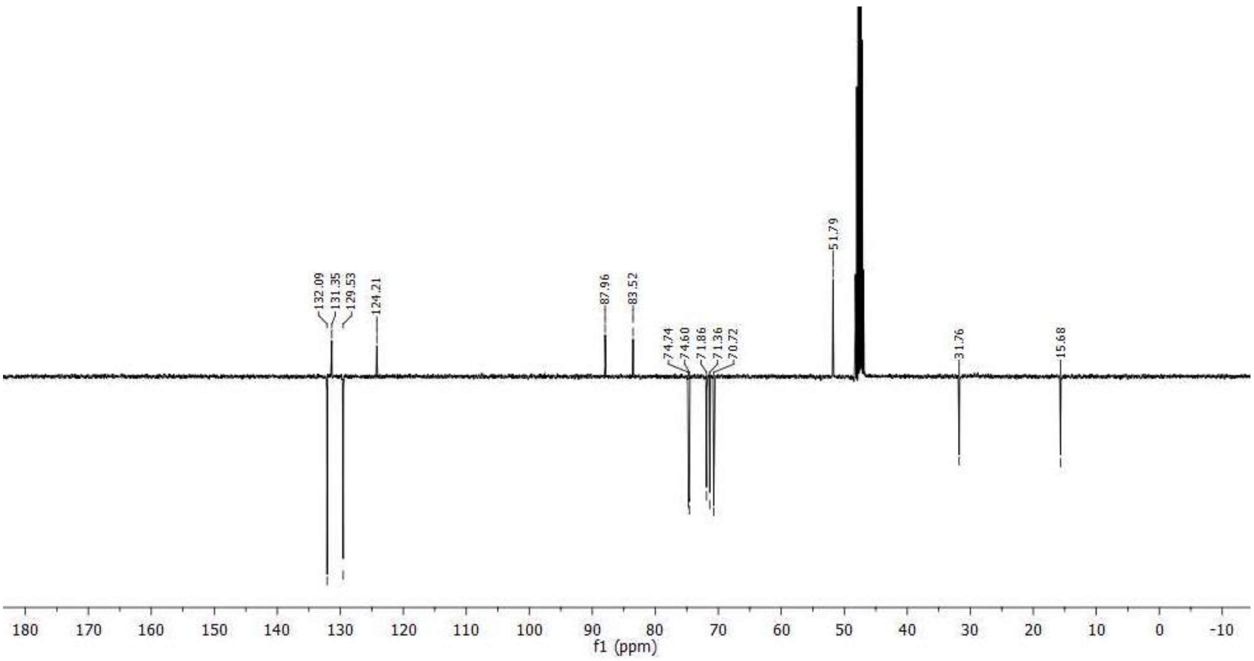

1-(4-(2,3,4-tri-*O*-acetyl β-L-fucopyranosylethynyl)phenyl)-*N*-methylmethanamine 38

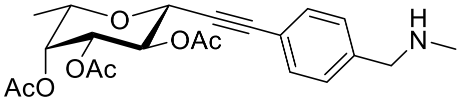

38

^{1}H NMR (400 MHz, $CDCl_3$)

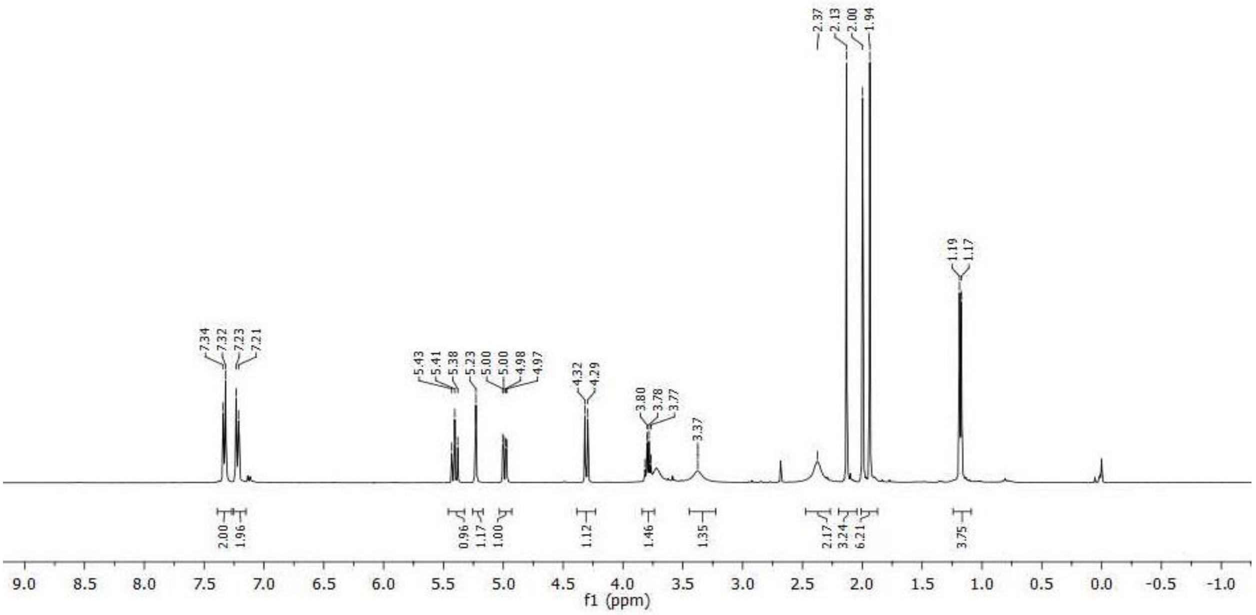

^{13}C NMR (100 MHz, $CDCl_3$)

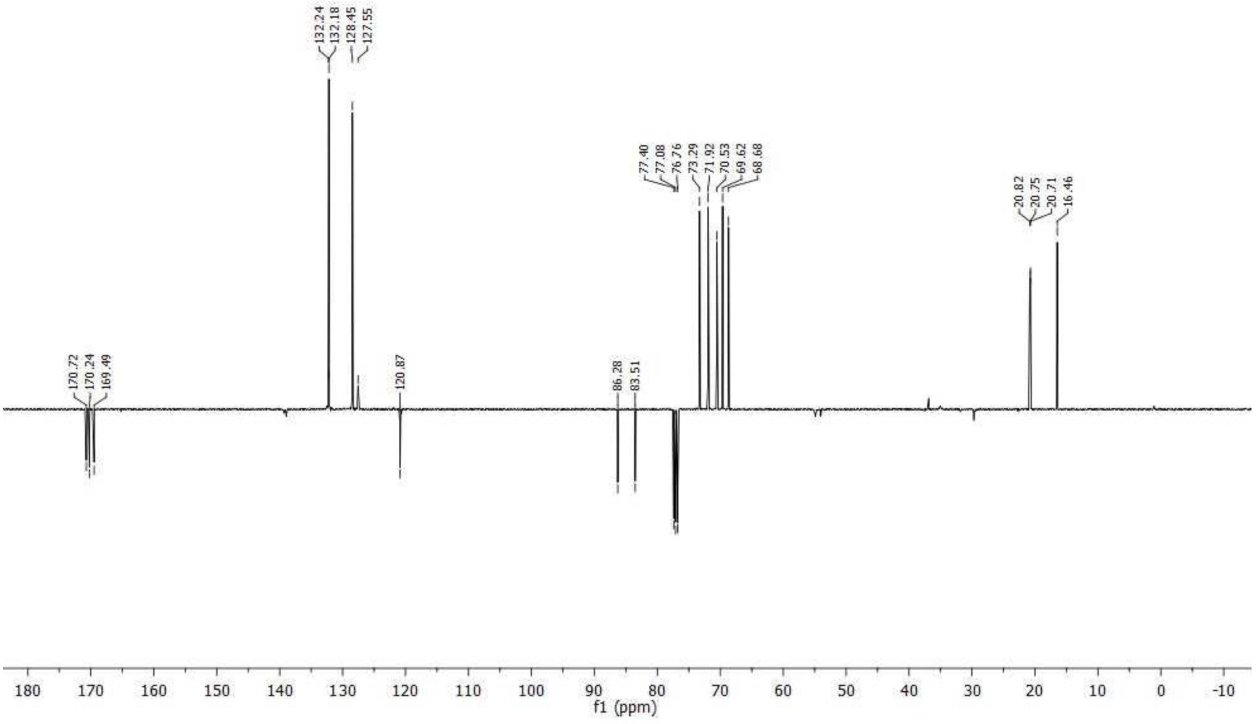

***N’, N’’*-bis(*tert*-butoxycarbonyl) 1-(4-(2,3,4-tri-*O*-acetyl β-L-fucopyranosylethynyl)benzyl)-*N*-methylguanidine 20g**

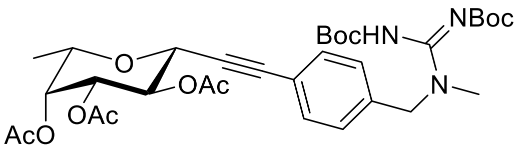

20g

^{1}H NMR (400 MHz, CDCl$_3$)

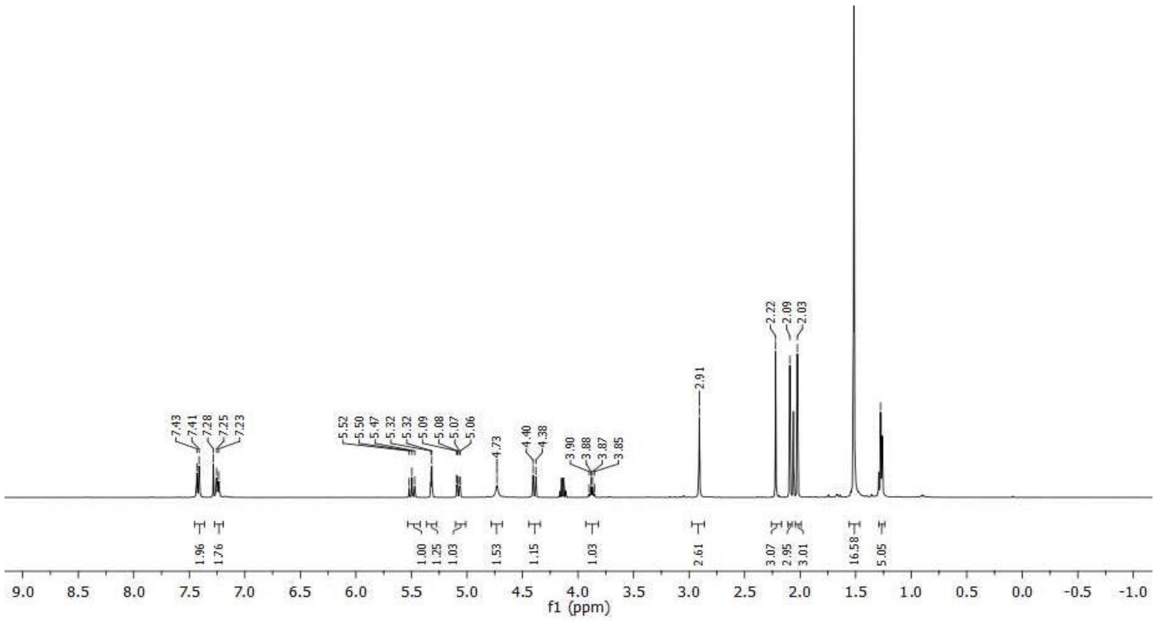

^{13}C NMR (100 MHz, CDCl$_3$)

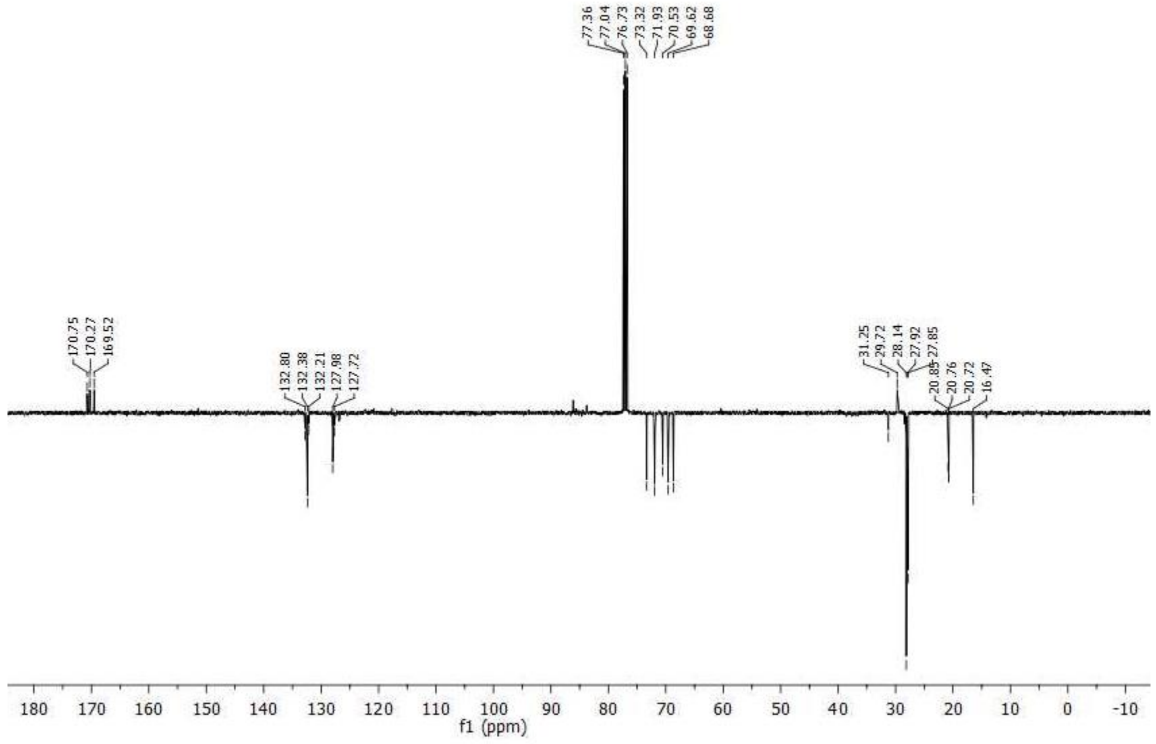

***N',N''*-bis(*tert*-butoxycarbonyl)-1-(4-(β-L-fucopyranosylethynyl)benzyl-*N*-methylguanidine 21g**

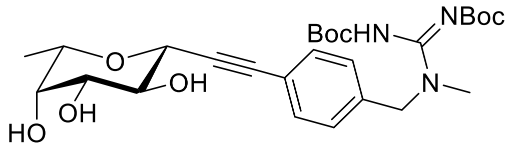

21g

^{1}H NMR (400 MHz, CD_3OD)

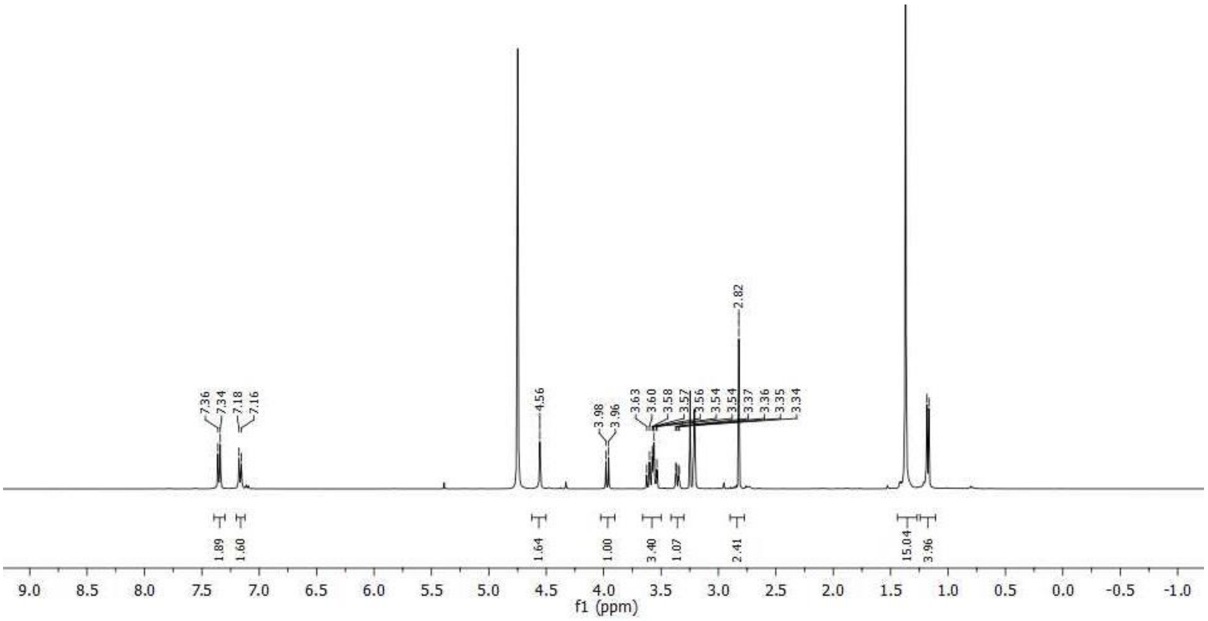

HSQC (400 MHz, CD_3OD)

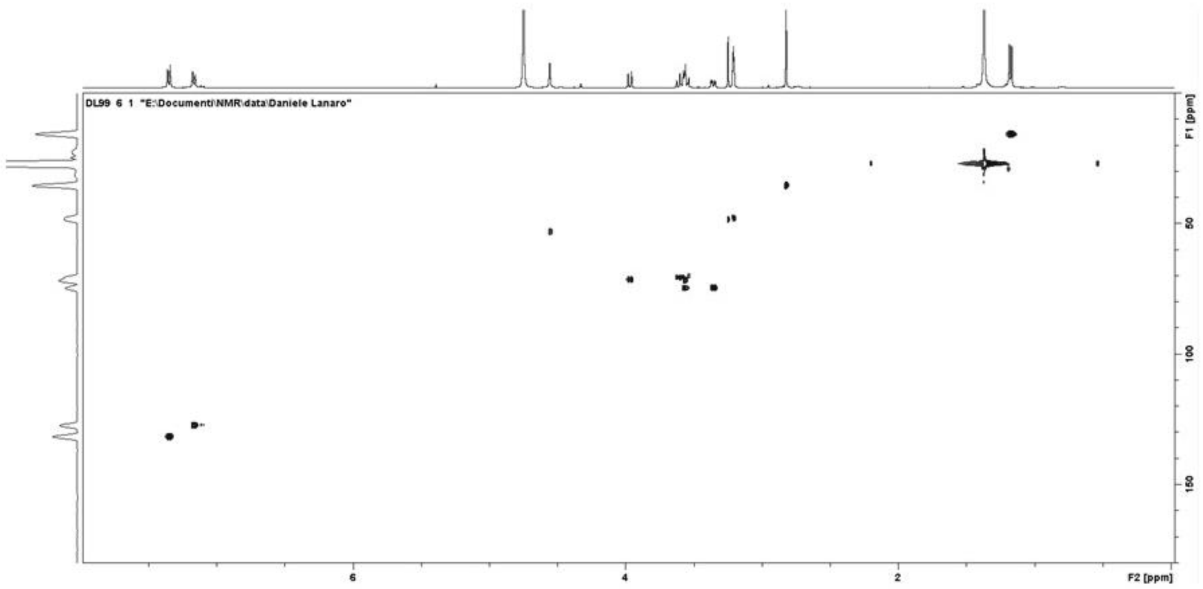

1-(4-(β-L-fucopyranosylethynyl)benzyl)-*N*-methylguanidine 22g

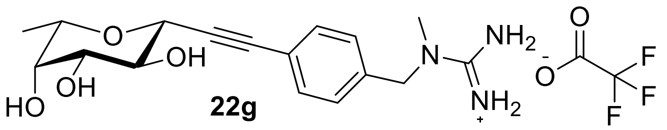

22g

^{1}H NMR (400 MHz, CD_3OD)

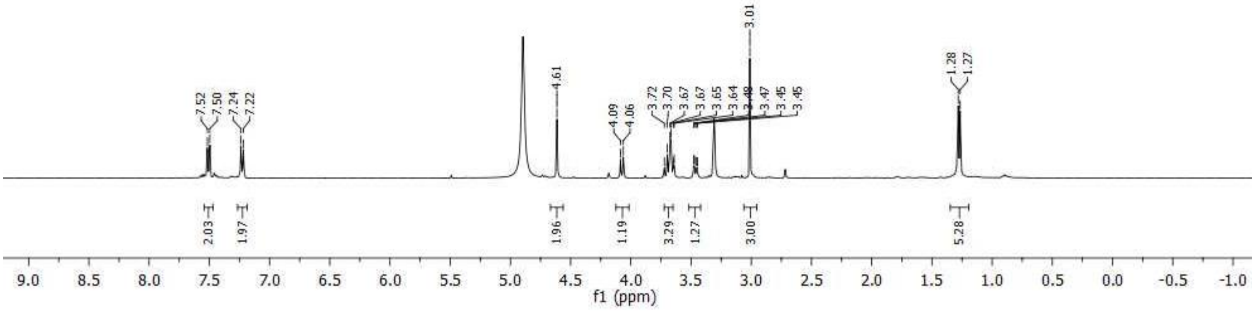

^{13}C NMR (100 MHz, CD_3OD)

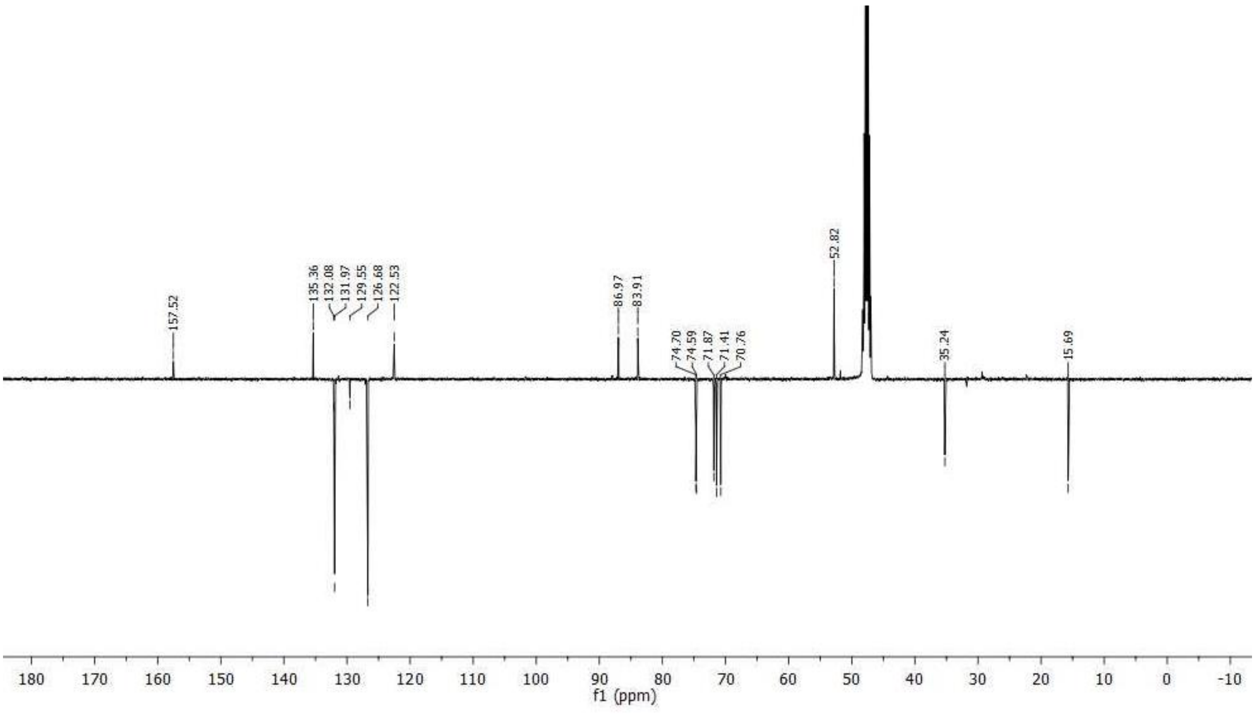

1-(Quinolin-6-yl)-3-(β-L-fucopyranosyl)prop-2-yn-1-ol 22f

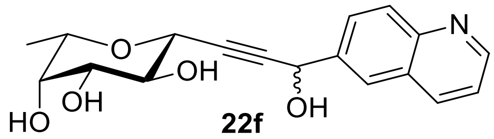

^{1}H NMR (400 MHz, CD_3OD)

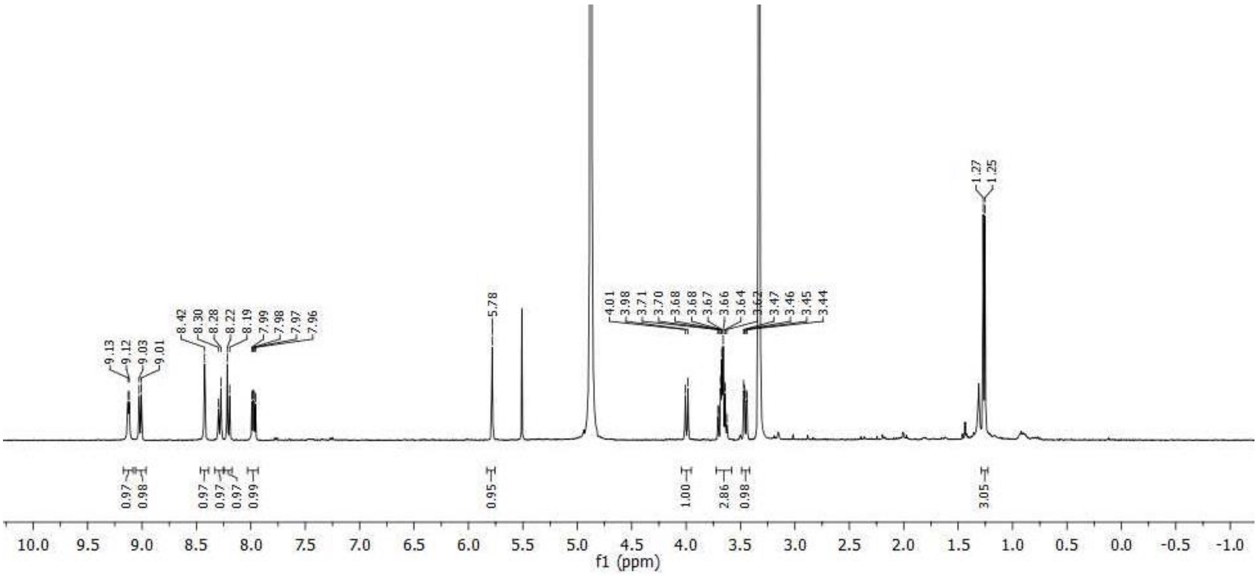

^{13}C NMR (100 MHz, CD_3OD)

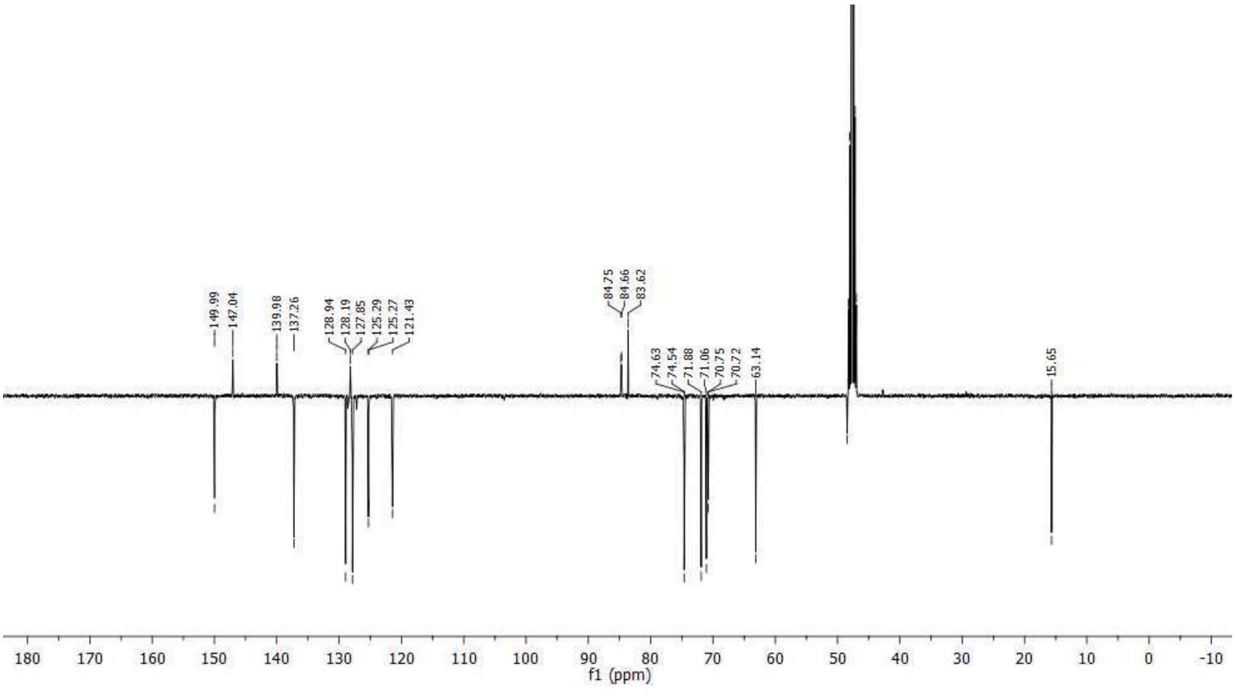

1-(4-(β-L-fucopyranosylvinyl)phenyl)-*N*-methylmethanamine 28b

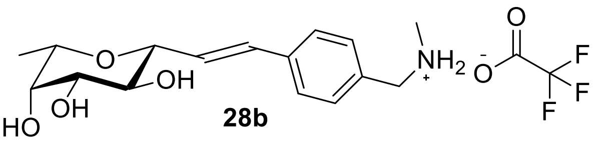

^{1}H NMR (400 MHz, CD_3OD)

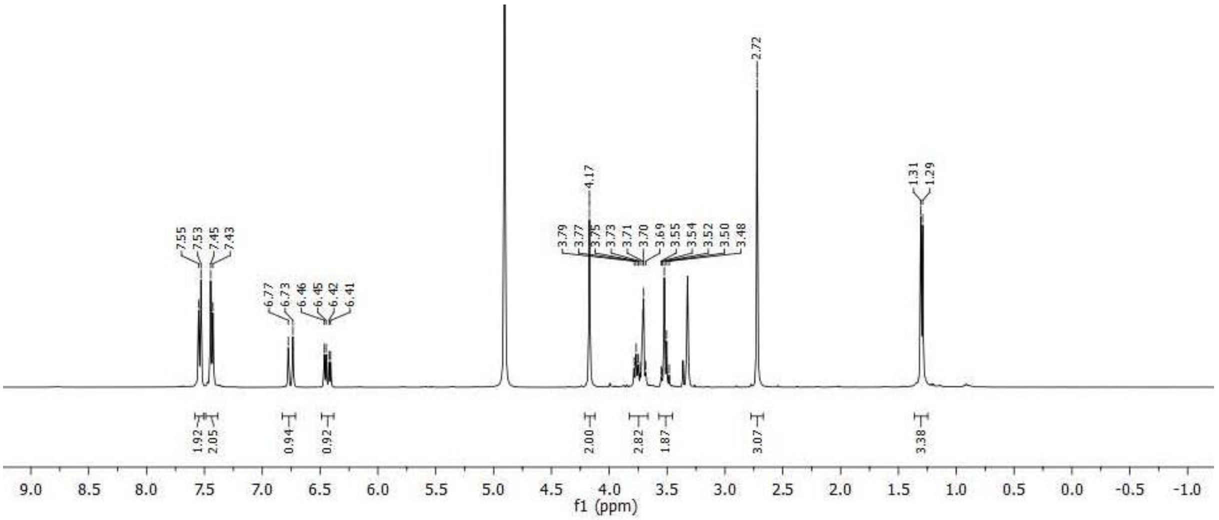

^{13}C NMR (100 MHz, CD_3OD)

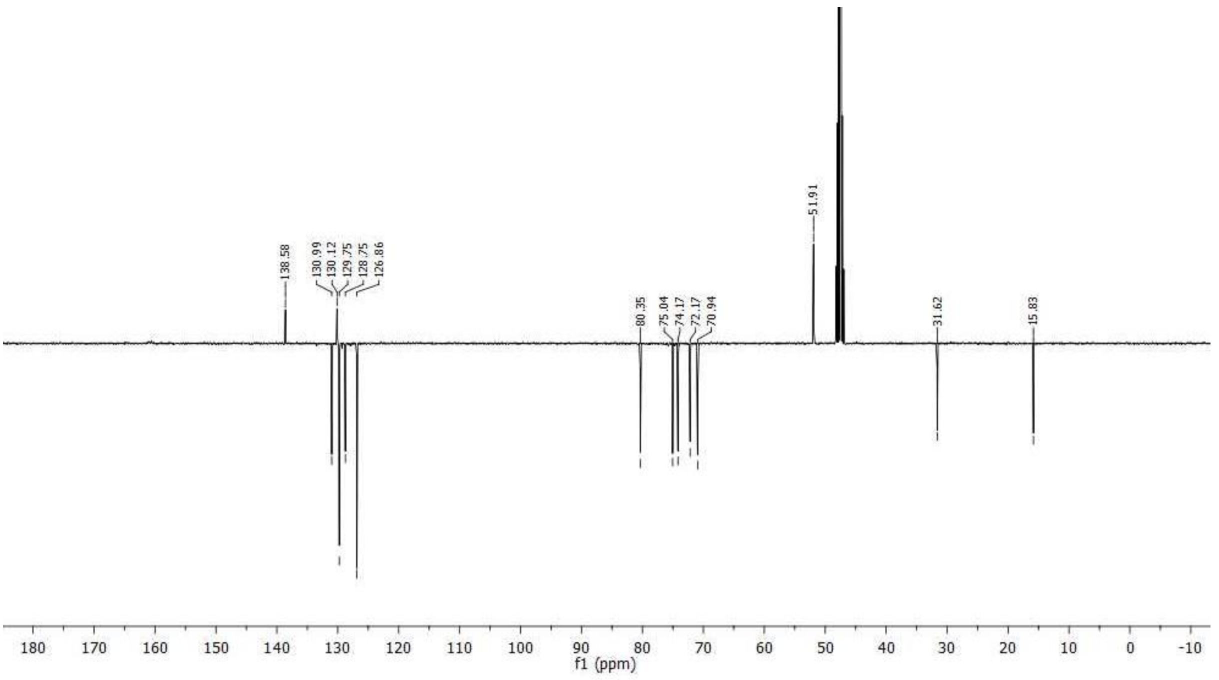

Supplement: Supplementary file 1 — cb2c00532_si_001.pdf [file cb2c00532_si_001.pdf]
